# Late-Stage Metastatic Melanoma Emerges through a Diversity of Evolutionary Pathways

**DOI:** 10.1158/2159-8290.CD-22-1427

**Published:** 2023-03-28

**Authors:** Lavinia Spain, Alexander Coulton, Irene Lobon, Andrew Rowan, Desiree Schnidrig, Scott T.C. Shepherd, Benjamin Shum, Fiona Byrne, Maria Goicoechea, Elisa Piperni, Lewis Au, Kim Edmonds, Eleanor Carlyle, Nikki Hunter, Alexandra Renn, Christina Messiou, Peta Hughes, Jaime Nobbs, Floris Foijer, Hilda van den Bos, Rene Wardenaar, Diana C.J. Spierings, Charlotte Spencer, Andreas M. Schmitt, Zayd Tippu, Karla Lingard, Lauren Grostate, Kema Peat, Kayleigh Kelly, Sarah Sarker, Sarah Vaughan, Mary Mangwende, Lauren Terry, Denise Kelly, Jennifer Biano, Aida Murra, Justine Korteweg, Charlotte Lewis, Molly O'Flaherty, Anne-Laure Cattin, Max Emmerich, Camille L. Gerard, Husayn Ahmed Pallikonda, Joanna Lynch, Robert Mason, Aljosja Rogiers, Hang Xu, Ariana Huebner, Nicholas McGranahan, Maise Al Bakir, Jun Murai, Cristina Naceur-Lombardelli, Elaine Borg, Miriam Mitchison, David A. Moore, Mary Falzon, Ian Proctor, Gordon W.H. Stamp, Emma L. Nye, Kate Young, Andrew J.S. Furness, Lisa Pickering, Ruby Stewart, Ula Mahadeva, Anna Green, James Larkin, Kevin Litchfield, Charles Swanton, Mariam Jamal-Hanjani, for the PEACE Consortium, Samra Turajlic

**Affiliations:** 1 Cancer Dynamics Laboratory, The Francis Crick Institute, London, United Kingdom.; 2 Skin and Renal Unit, Royal Marsden NHS Foundation Trust, London, United Kingdom.; 3 Department of Medical Oncology, Peter MacCallum Cancer ­Centre, Melbourne, Australia.; 4 Tumour Immunogenomics and Immunosurveillance (TIGI) Lab, UCL Cancer Institute, London, United Kingdom.; 5 Cancer Evolution and Genome Instability Laboratory, The Francis Crick Institute, London, United Kingdom.; 6 Sir Peter MacCallum Department of Oncology, The University of Melbourne, Victoria, Australia.; 7 The Royal Marsden Hospital, London, United Kingdom.; 8 The Institute of Cancer Research, Kensington and Chelsea, United Kingdom.; 9 European Research Institute for the Biology of Ageing, University of Groningen, University Medical Centre Groningen, Groningen, the Netherlands.; 10 St. John's Institute of Dermatology, Guy's and St Thomas’ Hospital NHS Foundation Trust, London, United Kingdom.; 11 Precision Oncology Center, Oncology Department, Lausanne University Hospital, Lausanne, Switzerland.; 12 Gold Coast University Hospital, Queensland, Australia.; 13 The Francis Crick Institute, London, United Kingdom.; 14 Cancer Genome Evolution Research Group, Cancer Research UK Lung Cancer Centre of Excellence, UCL Cancer Institute, London, United Kingdom.; 15 Cancer Research UK Lung Cancer Centre of Excellence, UCL Cancer Institute, London, United Kingdom.; 16 Drug Discovery Technology Laboratories, Ono Pharmaceutical Co., Ltd. Osaka, Japan.; 17 University College London Hospital, London, United Kingdom.; 18 Guy's and St Thomas’ NHS Foundation Trust, London, United Kingdom.; 19 Cancer Metastasis Laboratory, University College London Cancer Institute, London, United Kingdom.; 20 Department of Medical Oncology, University College London Hospitals, London, United Kingdom.

## Abstract

Understanding the evolutionary pathways to metastasis and resistance to immune-checkpoint inhibitors (ICI) in melanoma is critical for improving outcomes. Here, we present the most comprehensive intrapatient metastatic melanoma dataset assembled to date as part of the Posthumous Evaluation of Advanced Cancer Environment (PEACE) research autopsy program, including 222 exome sequencing, 493 panel-sequenced, 161 RNA sequencing, and 22 single-cell whole-genome sequencing samples from 14 ICI-treated patients. We observed frequent whole-genome doubling and widespread loss of heterozygosity, often involving antigen-presentation machinery. We found *KIT* extrachromosomal DNA may have contributed to the lack of response to KIT inhibitors of a KIT-driven melanoma. At the lesion-level, *MYC* amplifications were enriched in ICI nonresponders. Single-cell sequencing revealed polyclonal seeding of metastases originating from clones with different ploidy in one patient. Finally, we observed that brain metastases that diverged early in molecular evolution emerge late in disease. Overall, our study illustrates the diverse evolutionary landscape of advanced melanoma.

**Significance:**

Despite treatment advances, melanoma remains a deadly disease at stage IV. Through research autopsy and dense sampling of metastases combined with extensive multiomic profiling, our study elucidates the many mechanisms that melanomas use to evade treatment and the immune system, whether through mutations, widespread copy-number alterations, or extrachromosomal DNA.

*See related commentary by Shain, p. 1294.*

*This article is highlighted in the In This Issue feature, p. 1275*

## Teaser

Metastatic melanoma evolution was illustrated through research autopsy and extensive multiomic profiling and revealed the diverse routes to treatment resistance, including extensive copy-number alterations, mutations in key drivers, and extrachromosomal DNA.

## INTRODUCTION

In the last decade, treatment options for advanced melanoma have improved significantly (1, 2). Most notable has been the development of immune-checkpoint inhibitor (ICI) therapy, with reported 5-year overall survival of 52% in patients receiving combination PD-1/CTLA4 blockade (3). Nonetheless, a large proportion of metastatic melanomas remain refractory to systemic therapy, highlighting the need to understand therapy failure. Studies focusing on cohort size rather than multitumor profiling provide a snapshot of the landscape of disease (4–6) but cannot adequately inform potential evolutionary trajectories in the progression to treatment resistance and death. Therefore, it is essential to complement these studies with in-depth sampling and analysis of the evolution of metastatic melanoma.

The research autopsy has emerged as a method to overcome the limitations of tumor sampling during life (7), with several examples in melanoma (8–11). However, various areas remain hitherto unexplored, including the genotype-to-phenotype link via the analysis of transcriptomic and radiologic data; the application of an extensive multiregional sampling design, shown to reveal subclones that are missed from single samples (12–14); as well as phylogenetic analyses that utilize clone-based trees rather than sample trees (15).

Another area of interest is the significance of copy-number alterations in late-stage disease, ranging from focal somatic copy-number alterations (SCNA) through arm-level changes to whole-genome duplications. Chromosomal instability is known to enhance metastatic potential, increasing somatic copy-number alteration burden and fueling natural selection (12, 16), as well as supporting co-option of innate immune processes (17). Aneuploidy also correlates with reduced overall survival in anti–CTLA4-treated patients (18). Whole-genome doubling (WGD) is associated with poor prognosis in the pan-cancer setting (19) and has been reported in metastatic melanoma to varying degrees (6, 11). In addition, mirrored subclonal allelic imbalance (MSAI), indicative of parallel evolution of copy-number changes and ongoing chromosomal instability, is increased in WGD tumors (20). However, the degree to which WGD and chromosome instability are required for progression to late-stage disease remains unclear.

Here, we present an in-depth analysis of late-stage metastatic melanoma in the first 14 patients recruited to the Posthumous Evaluation of Advanced Cancer Environment (PEACE; NCT03004755) study, with extensive multiregional and multitumor sampling at autopsy (573 tumor samples in total). To address the gaps in knowledge discussed, we use exome, transcriptome, and high-depth panel sequencing data; radiologic imaging; and single-cell whole-genome sequencing data. We find that although many late-stage melanomas exhibit WGD, this process does not appear to be required for late-stage disease. However, when WGD does occur, it is usually associated with widespread loss of heterozygosity (LOH). We also associate lesion-level immunotherapy responses to recurrent copy-number changes, including *MYC* amplification and 1q gain. We identify polyclonal metastatic seeding in one patient through single-cell profiling, which was not identified solely from bulk sequencing data, indicating temporally separate waves of metastatic spread. In addition, we find that late-emerging brain metastases in this cohort often have distinct copy-number profiles compared with other tissue sites, diverging early in the phylogenetic tree but emerging late in the clinical disease course, suggesting a period of dormancy. Lastly, we observe frequent losses of antigen-presentation genes, such as *B2M* and *JAK2*, but do not detect significant loss of neoantigens compared with nonsynonymous mutations in general.

## RESULTS

### Cohort Overview

Our cohort comprises multiple melanoma subtypes, including cutaneous (seven, CRUKP2986, CRUKP1842, CRUKP2567, CRUKP9097, CRUKP6216, CRUKP6746, and CRUKP1599), acral (three, CRUKP9359, CRUKP2378, and CRUKP1047), mucosal (one, CRUKP6170), and melanomas of unknown primary site (MUP; three; CRUKP1614, CRUKP6553, and CRUKP5107), included in the PEACE study (Supplementary Table S1). In total, 573 samples from 387 tumors across 14 patients were profiled using either a gene panel (493 samples; mean ± SD coverage 606.27× ± 71.75; range, 375.61–1,089.46; see Methods for the panel design), whole-exome sequencing (WES; 222 samples; mean ± SD coverage 308.30× ± 122.58, range, 46.76–1330.26), RNA sequencing (RNA-seq; 161 samples), or a combination of the three (Supplementary Fig. S1). These samples included normal and tumor tissue taken at autopsy, with an emphasis on a comprehensive sampling of metastases in each patient (Supplementary Table S2). In addition, archival formalin-fixed, paraffin-embedded (FFPE) blocks from matched primary tumors and metastases surgically removed during life were also profiled when available. To assess intratumor heterogeneity, multiregional samples were taken from individual metastases where possible (49/156 exome-profiled tumors; median of 2 samples per tumor, range, 2–10). Patients received a median of 2.5 lines of treatment (range, 1–6; Supplementary Table S3). All patients received ICI during their disease course, with 9 of 14 patients treated with combination PD-1 + CTLA4 blockade and five with single-agent ICIs. Eight patients were treated with MAPK-targeting therapies, while one patient received two KIT inhibitors. Three patients were treated with chemotherapy, either temozolomide or dacarbazine in combination with a platinum agent.

**Figure 1.**
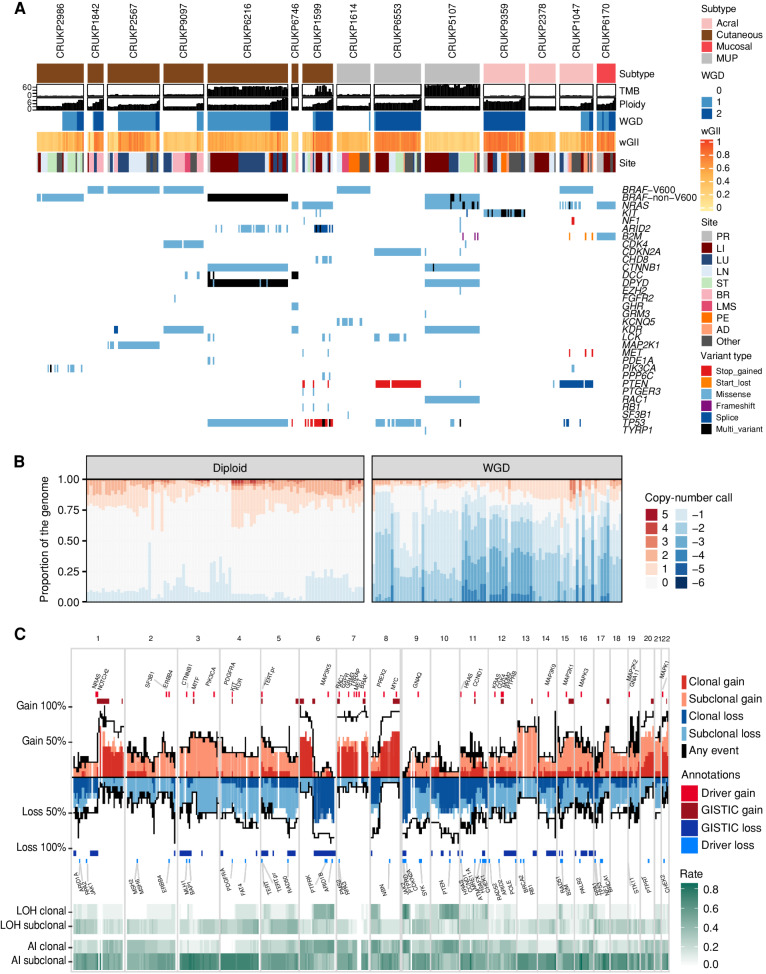
Driver mutations and SCNA overview. **A,** Genomic landscape of the cohort, illustrating mutations in key melanoma driver genes, TMB (total mutations/Mb), ploidy, WGD status, weighted genome instability index (wGII, an SCNA burden metric), and the anatomic site of each sample. “Multi-variant” indicates the presence of more than one variant in the same gene within one sample. Panel and WES samples are included. AD, adrenal; BR, brain; LI, liver; LMS, leptomeninges; LN, lymph node; LU, lung; PE, peritoneum; PR, primary; ST, soft tissue. **B,** The proportion of the genome altered by copy-number gains and losses per sample in diploid and WGD tumor samples. **C,** The frequency of copy-number gains and losses along the genome (based on WES data only). Dark red and blue indicate clonal events, and light red and blue indicate subclonal events. Also shown are frequency of clonal and subclonal LOH and AI.

We observe a wide range of tumor mutational burden (TMB; Fig. 1A). At the patient level, TMB based on WES ranged from 2.44 to 156 mutations per megabase (Mb) in cutaneous melanomas (mean ± SD 41.5 ± 55.1), 8.47 to 111 in MUPs (mean ± SD 42.8 ± 58.9), 1.11 to 6.79 in acral melanomas (mean ± SD 3.41 ± 2.99), and 2.18 in the mucosal melanoma, CRUKP6170. These data are consistent with prior reports (5) and reflect differing levels of mutagenic exposure between melanoma subtypes and patients, principally UV light and chemotherapy. As expected, we observed fewer insertions or deletions (indel) compared with single-nucleotide variants (SNV; mean ± SD 30.71 ± 17.95). All cases except CRUKP6553 and CRUKP2378 had a recognized melanoma driver alteration in *BRAF* (V600 in six cases, non-V600 in two), *NRAS* (three cases), or *KIT* (one case; detailed in Fig. 1A). Of interest, a cutaneous case (CRUKP1842) had a pathogenic germline mutation in *CDKN2A* (21) and had the lowest TMB of the sun-exposed cutaneous melanomas. In terms of somatic mutations, this patient had a clonal BRAF V600 mutation (Fig. 1) with no additional mutations in other known drivers. This was accompanied by clonal LOH at the *CDKN2A* locus.

Regarding SCNAs, we detected WGD in 11 cases (Fig. 1A), spanning all melanoma subtypes. We observed clonal WGD (WGD detectable in all the tumor samples in a given patient) in four cases (Fig. 1A). These mainly were single rounds of WGD except for CRUKP9359, in which two rounds of clonal WGD were evident. By incorporating mutational timing, we demonstrate instances of parallel WGD in distinct subclones of the same patient (CRUKP6216, CRUKP1599, CRUKP6553, CRUKP9359, and CRUKP1047), suggesting that WGD confers a selective advantage (example in Supplementary Fig. S2A and S2B). In contrast, most tumor regions in patients CRUKP2986, CRUKP9097, and CRUKP1614 were diploid, with subclonal WGD limited to a small number of tumor regions, consistent with WGD appearing late in tumor evolution. An intermediate example is CRUKP2567, in which WGD was detected in all the thoracic metastases but not in the brain metastasis (Fig. 1A). There was no significant association between the subtype of melanoma and the presence of WGD or clonal WGD, potentially limited by the size of the cohort (chi-squared tests *P* = 0.8 and *P* = 0.4, respectively).

In the context of WGD and increasing chromosome copies (ploidy), we observed significantly elevated weighted genome instability index (wGII, see Methods; R-squared = 0.6; *P* < 2.2e−16; Supplementary Fig. S3A and S3B), driven by copy-number losses (Fig. 1B). The median ploidy of WGD tumors was 3.82. WGD was also associated with increased intrapatient heterogeneity of copy-number alterations (subclonal wGII, the burden of copy-number events not present in all tumors from a case; Wilcoxon test *P* = 0.006; Supplementary Fig. S3C). Regions with recurrent clonal LOH were enriched in tumor suppressor genes (chi-squared test of genes with LOH in at least three patients vs. other genes, *P* = 0.038), including 9p (*CDKN2A*), which we observed in 10 of 14 cases, 10q (*PTEN*) in nine cases, and 6q (*ARID1B*) in six cases. In addition, eight cases had clonal LOH of 8p, although the selective drivers behind this event are unclear. Allelic imbalance (AI) was common (Fig. 1C), and subclonal AI was enriched in WGD samples. At the gene level, the most common SCNA was amplification of *BRAF*, observed in 12 of 14 patients (clonal in seven and subclonal in five), including two cases without activating *BRAF* mutations (CRUKP6746 and CRUKP2378).

### Tumor Mutational Signatures and Evolutionary Histories

**Figure 2.**
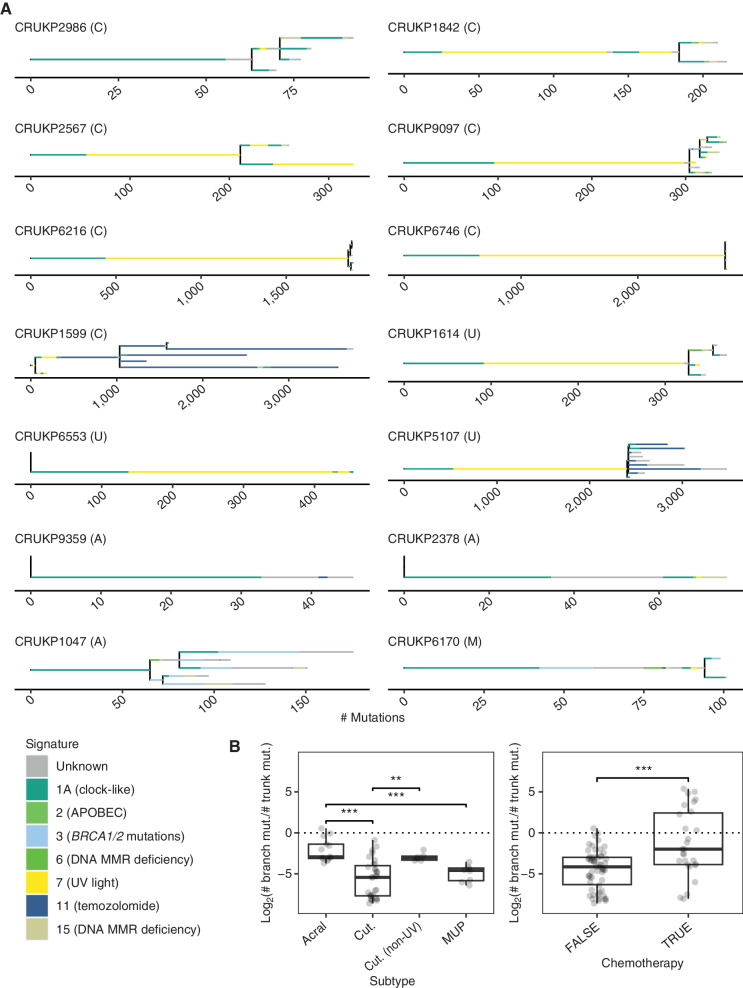
**A,** Phylogenies inferred for the 14 patients. Only WES samples are included. Letters in brackets indicate melanoma subtype: A = acral, C = cutaneous, M = mucosal, U = melanoma of unknown primary. Branch length is proportional to the number of mutations. Branch colors represent the mutational signatures of the mutations. For clarity, only the most common mutational signatures are shown; the remainder are categorized as “unknown.” Scale bars indicate the number of mutations. The legend includes etiologies for each signature (24). MMR, mismatch repair. **B,** Boxplots indicate the ratio of subclonal mutations (length of branches) to clonal mutations (length of the trunk) by subtype and chemotherapy status. Values smaller than zero indicate the dominance of truncal mutations. Mann–Whitney *U* test was used for statistical comparisons (**, *P* < 0.01; ***, *P* < 0.001). Cut., cutaneous; mut., mutation.

Tumors exhibited a range of mutational signatures, reflecting melanoma subtype and/or chemotherapy exposure (Fig. 2A). In cutaneous melanoma, with the exception of a patient with a sun-protected perianal primary melanoma (CRUKP2986), most clonal mutations were attributed to mutational signature 7, which is associated with UV light exposure (22). Signature 7 was also dominant in all cases of MUP, suggesting they arose from sun-exposed primary melanomas that subsequently regressed (23). As expected, the dominant signature in patients with acral and mucosal melanomas was signature 1A, which is caused by the deamination of 5-methylcytosine and correlates with age (24).

To investigate the evolutionary trajectories of metastatic melanomas, we constructed clone-level phylogenies based on the SNVs and small indels identified in the WES data (see Methods). We observed diverse phylogenetic structures in our cohort (Fig. 2A). CRUKP6553, CRUKP9359, and CRUKP2378 followed a linear evolutionary trajectory (25) with no apparent branching and a small number of subclones (ranging from two to three). The pattern of metastatic seeding in these patients was monoclonal—that is, all metastases were seeded by the same single clone (26). In contrast, CRUKP2986, CRUKP9097, CRUKP1599, CRUKP1614, CRUKP5107, and CRUKP1047 were characterized by branched phylogenies and multiple subclones (ranging from 7 to 13). The number of clones was not associated with the melanoma subtype.

We observed instances of polyclonal seeding, in which genetically distinct clones in the primary tumor seeded different metastatic sites, in most patients (10 of the 14; see Methods). For example, in CRUKP2567, the primary tumor was polyclonal, with subclones specific to brain and thoracic metastases. Finally, we examined individual metastases for evidence of polyclonality, defined as the presence of clones from independent branches of the SNV/indel phylogeny (26). The majority of metastases appeared to be monoclonal at the SNV/indel level, consistent with an evolutionary bottleneck during the metastatic colonization and absence of frequent cross-metastatic seeding in this cohort; some examples of polyclonal metastases were seen in pericardial, lung, and brain metastases in CRUKP9097; lung metastases in CRUKP6216; a lymph node metastasis in CRUKP159; and a soft-tissue metastasis in CRUKP5107 (Supplementary Fig. S4).

TMB was associated with the melanoma subtype and exposure to chemotherapy (Fig. 2B). Three patients received chemotherapy (either temozolomide or dacarbazine with cisplatin), of whom two, CRUKP1599 and CRUKP5107, had increased subclonal TMB (as shown by branch lengths; Fig. 2A) characterized by mutational signature 11, which is linked to temozolomide exposure (22). We did not observe this mutational signature in the third patient treated with chemotherapy (CRUKP6170), potentially due to much shorter exposure immediately prior to death (2 cycles compared with >5 cycles of chemotherapy in CRUKP1599 and CRUKP5107; Supplementary Table S3). We found clones shared by liver and lymph node metastases that were characterized by a chemotherapy signature in case CRUKP1599. We inferred these clones appeared after treatment initiation and are the result of intermetastatic seeding (Supplementary Fig. S4A–S4C).

To assess the patterns of evolution at the level of copy number, we used MEDICC2 (27) to build SCNA sample trees (Supplementary Fig. S5). SCNA-based trees were consistent with the SNV phylogenies in most cases, but in cases with linear SNV phylogeny (CRUKP6553, CRUKP9359, and CRUKP2378) they revealed SCNA-driven subclonal diversification (Fig. 2; Supplementary Fig. S5). A cohort-level SCNA tree (Supplementary Fig. S6) demonstrated samples clustered by patient and not subtype, in agreement with the SCNA frequency along the genome being similar across melanoma subtypes (Supplementary Fig. S7A–S7C).

We next sought to examine whether the metastatic site influenced tumor SCNAs, either through shared ancestry of tumors within an organ or through convergent evolution of tumors within an organ due to selective pressure imposed by their shared environment. We observed that copy-number profiles of individual metastatic tumors within the same metastatic site were clustered together in some cases (e.g., lung metastases in CRUKP1599; Supplementary Fig. S5) but not in others (e.g., liver metastases in CRUKP6216; Supplementary Fig. S5). In addition, we calculated the Fst (fixation index, commonly used in population genetics; ref. 28) for each patient. A high Fst indicates that SCNAs of tumors between metastatic sites are varied, whereas low Fst values indicate that SCNAs of tumors vary within metastatic sites. In our cohort, the mean Fst was mostly explained by the number of metastatic sites and samples (as indicated by a linear model, R2 = 0.75, *P* = 6.8e−4), although the three MUP cases deviated more from the model, suggesting greater organ-specific SCNA diversification.

To investigate the parallel evolution of SCNAs, we looked for evidence of MSAI (see Methods), where the same SCNA event occurs more than once but involves different alleles. MSAI was common in our cohort, with at least one MSAI event observed in >95% of metastatic samples and 20% of all AI events being mirrored. Notable was the absence of MSAI in chromosome 9, with independent subclonal LOH events always affecting the same allele even in patients without mutations in *CDKN2A* (CRUKP2986, CRUKP1599, and CRUKP1047). The other region with evidence of conserved allele-specific patterns of LOH was 17p, which harbors *TP53*. Although some losses occurred in samples with *TP53* mutations, we also detected subclonal LOH of the same allele in patients without *TP53* mutations (CRUKP2986 and CRUKP1842). A potential reason for these patterns is the fixation of a methylated allele with recurrent loss of the wild-type allele.

### Distinctive Features of Late-Emerging Brain Metastases

**Figure 3.**
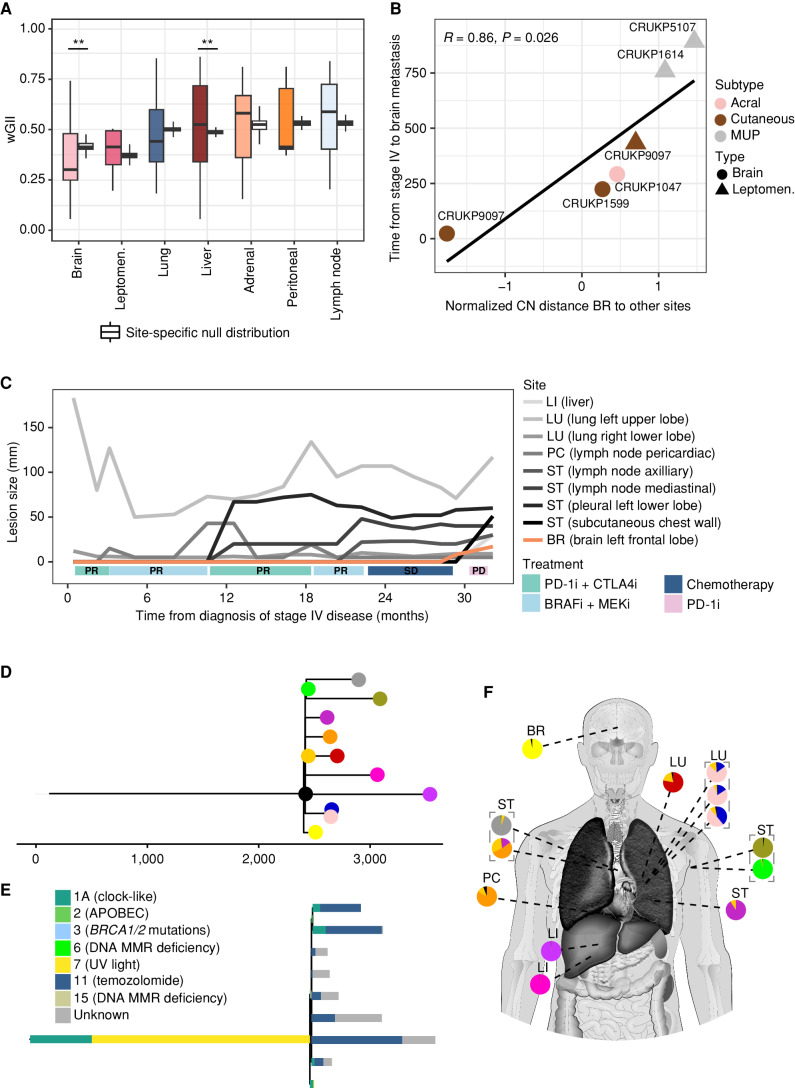
Late-emerging brain metastases have a lower copy-number burden. **A,** wGII per metastatic site. Site-specific null distributions of mean wGII were generated by randomizing sample sets (from any metastatic site) while keeping patient contributions constant (see Methods). **, *P* ≤ 0.01. Leptomen., leptomeninges. **B,** Correlation between brain copy-number (CN) distance to other sites and time of emergence of brain metastases after stage IV diagnosis in days. **C,** Growth dynamics of tumors in patient CRUKP5107. The brain lesion (in orange) was detected in only the last two scans after the targeted therapy [BRAF inhibitor (i) + MEKi], ICI (PD-1i + CTLA4i), and chemotherapy courses. PD, progressive disease; PR, partial response; SD, stable disease. **D,** SNV and indel phylogenetic tree of tumor clones in patient CRUKP5107. **E,** The mutational signature contributions to each clone in the phylogeny in **D** are shown. MMR, mismatch repair. **F,** The anatomic distribution of clones. Each pie chart represents a sample with its clonal composition indicated by the colors. A multiregional sampling of the same tumor is indicated by the gray dashed lines. BR, brain; LI, liver; LU, lung; PC, pericardium; ST, soft tissue.

We next considered genomic differences relative to the site of metastases. We observed the lowest burden of SCNAs, expressed as wGII, in brain metastases in this cohort (linear mixed-effects model *P* = 0.042; permutation test *P* = 0.009; Fig. 3A). Clones seeding the brain often diverged early in SNV and SCNA trees (Supplementary Figs. S5 and S6), with brain metastases emerging late in the clinical course of metastatic disease. Overall, the correlation between early evolutionary divergence and late clinical emergence of brain metastases was consistent across patients (*R* = 0.86, *P* = 0.026; Fig. 3B), and particularly apparent in CRUKP1614 and CRUKP5107. Our observations are consistent with the scenario in which the clone seeding the brain is characterized by lower fitness due to lower SCNA burden and results in later emergence of detectable metastasis. In support of the latter notion, the transcriptomic data showed downregulation of DNA replication in the brain compared with thoracic disease (*q*-value = 5.23e-6). We found further support for this hypothesis in the case of patient CRUKP5107, who was treated with 6 cycles of cisplatin and dacarbazine (Fig. 3C). The chemotherapy mutational signature was absent only from brain metastasis (24), which was characterized by early clonal divergence and late presentation relative to other metastatic sites (Fig. 3D–F).

In contrast, liver metastases harbored a numerically higher burden of SCNAs relative to other sites, although these results did not reach statistical significance (permutation test *P* = 0.003; mixed-effects model *P* = 0.38). Liver metastases with a larger evolutionary divergence had a nonsignificant trend toward emerging earlier in the disease course (SCNA distance to other metastases vs. time of emergence *R* = −0.27, *P* = 0.52; Supplementary Fig. S8). Our observations are consistent with the typically aggressive clinical behavior of melanoma liver metastases, including reduced sensitivity to ICIs (29).

### Melanomas Develop Resistance to Therapy via Mutational and Copy-Number Mechanisms

Most patients treated with MAPK-targeted therapy experience resistance following an initial response, with median progression-free survival of 9.3 months for dabrafenib and trametinib (30). In our cohort, we observe a number of previously described mechanisms of resistance that converge on reactivation of the MAPK pathway (31), including *NRAS* mutations (in CRUKP1047 and CRUKP5107), parallel evolution of distinct subclonal *NRAS* mutations (Q61H and G13R in CRUKP5107), and a *MAP2K1* mutation (in CRUKP2567; Fig. 1A).

**Figure 4.**
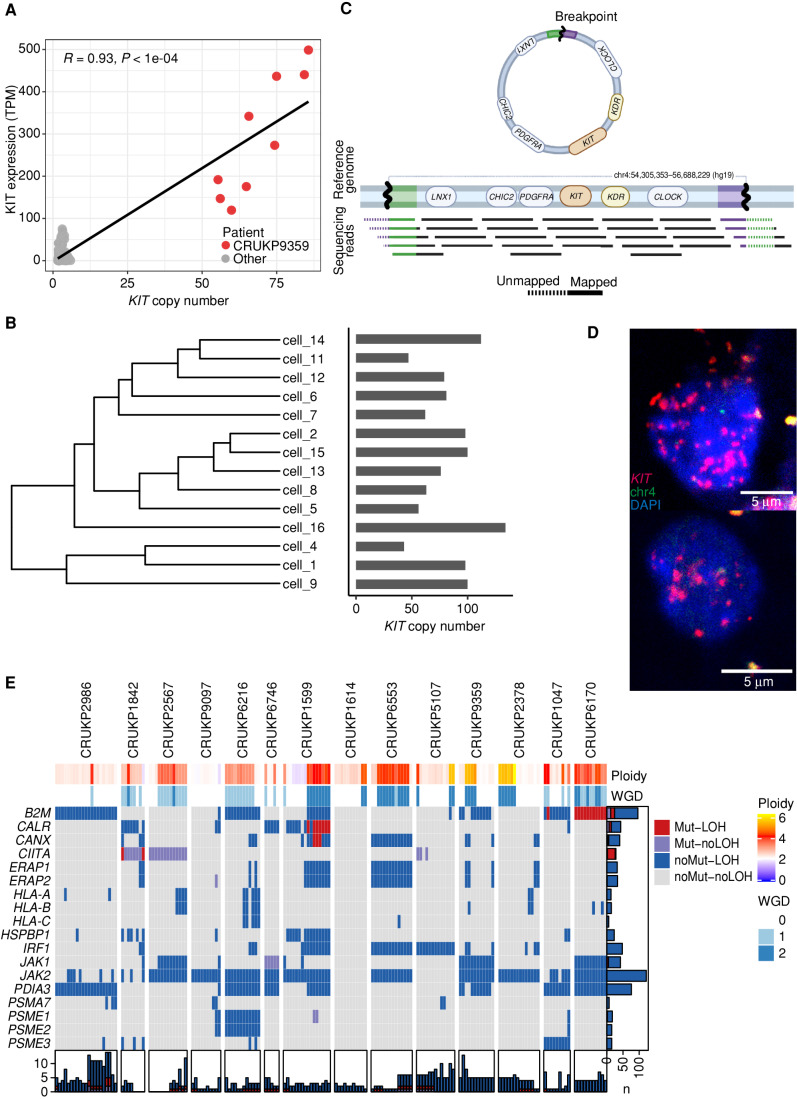
Mechanisms of resistance to therapy. **A,***KIT* copy number vs. KIT expression in matching exome and RNA-seq samples. TPM, transcripts per million. **B,** Hierarchical clustering tree of SCNAs found in the single cells of a representative sample of CRUKP9359. Bars on the right show the copy number of *KIT* in each cell. **C,** Diagram of split reads mapping at the edges of the amplified region, from which a circular structure can be inferred. Created with BioRender.com. **D,** Images showing FISH probes against *KIT* (red) in individual cells. **E,** Heat map of alterations in antigen-presentation genes in the exome data. Each column represents a sample. LOH events are shown in red and blue, and nonsynonymous mutations are in red and purple. Bars on the *x*-axis show the number of genes altered in each sample, whereas *y*-axis bars show the number of samples altered per gene, colored by the type of event.

One case of a *KIT* mutant melanoma (CRUKP9359, clonal V650A) also harbored an extreme clonal copy-number amplification at the KIT-encoding locus, with corresponding elevated KIT expression (Fig. 4A). Single-cell sequencing of a representative metastasis revealed cell-level *KIT* copy number of 43 to 134 (Fig. 4B, right), a range reported in the context of extrachromosomal DNA (ecDNA; ref. 32). We observed split sequencing reads in the single-cell pseudobulk, suggestive of ecDNA (Fig. 4C). We sought to further validate this by fluorescence *in situ* hybridization (FISH) and observed a large number of KIT copies colocalizing with DAPI-stained nuclear DNA in this case (Fig. 4D). Through hierarchical clustering of the single-cell level SCNAs (excluding KIT), we observed evidence of random segregation of ecDNA, which is consistent with this wide range of copy number (Fig. 4B, left). We note that this patient was treated with two consecutive KIT inhibitors and the extrachromosomal amplification of the drug target may have contributed to the lack of response to these agents.

All patients in this cohort received ICIs [either as single agent (PD-1 or CTLA4 inhibitors) or in combination (PD-1 and CTLA4 inhibitors)] and exhibited either primary or acquired resistance to therapy, and all were ICI refractory at the time of death and tumor sampling. Across the cohort, we observed alterations in the known drivers of ICI resistance (33), including LOH in *JAK2* in 12 patients and LOH in *B2M* in eight patients (Fig. 4E). The rate of LOH at these sites was higher than expected by chance for *JAK2* (permutation test *P* < 0.005) though not reaching significance for *B2M* (permutation test *P* = 0.09). We additionally observed clonal mutations in *JAK1* (p.S961L) in CRUKP6746 and in *B2M* (p.R101P) with accompanying LOH in CRUKP6170 (Fig. 4E). We found similar levels of HLA LOH (5% subclonal) to those previously reported for metastatic melanoma (3% subclonal; ref. 20), and there was no enrichment for LOH in the region encoding HLA genes (Fig. 4E; ref. 34). We did not observe any mutations in HLA genes.

Previous work (35–37) has demonstrated within-patient heterogeneity between tumors in terms of acquired mechanisms of resistance to targeted MAPK pathway inhibition therapies. This is likely due to the stochastic nature of mutational processes between tumors, with selection acting on the first viable mechanism that arises in each tumor. We sought to examine whether this was also the case for the putative mechanisms of ICI resistance identified in our cohort, and also whether tumor site influenced these mechanisms. Hierarchical clustering of tumors by antigen-presentation machinery alterations (as in Fig. 4E) revealed that tumors for the most part cluster by patient rather than by site (Supplementary Fig. S9). In addition, many tumors have clonally identical antigen-presentation alteration profiles within patients (Fig. 4E; Supplementary Fig. S9).

Genomic regions subject to copy-number loss in our cohort overlapped with those previously associated with ICI resistance (refs. 38, 39; Wilcoxon test *P* < 2.2e-16; Supplementary Fig. S10). Genes encoded by these regions were lost more often than expected in both cutaneous and acral melanomas (60.93% and 81.62% of genes, respectively; permutation tests, *P* < 0.05), including *PTEN*, previously linked to immune evasion in melanoma (40).

We next looked for evidence of immunoediting—that is, copy-number losses at loci encoding neoantigens, a reported mechanism of immune evasion under ICI (41)—but we observed no significant bias in favor of predicted neoantigen loss compared with nonsynonymous mutations across the cohort (Fisher exact test). Relatedly, we looked for evidence of downregulation in the expression of neoantigens using a binary classification of expression as in ref. 42, first for clonal neoantigens and then for all neoantigens (clonal and subclonal). This association was significant in several patients, indicating downregulation of neoantigens in patients CRUKP2986, CRUKP6746, and CRUKP1599, which did not appear to be influenced by gene copy-number dosage (Supplementary Fig. S11). However, patients CRUKP5107 and CRUKP9097 had contrasting relationships, with nonneoantigenic nonsynonymous mutations being downregulated over neoantigens. Only one of these (CRUKP5107) appeared to be influenced by gene dosage. Expanding the analysis to all neoantigens (clonal and subclonal), CRUKP2986, CRUKP1599, and CRUKP6553 had significant downregulation of neoantigens compared with nonneoantigenic nonsynonymous mutations, whereas CRUKP5107 and CRUKP9359 had the opposite relationship. While histologically, tumor-infiltrating lymphocyte scores were generally low at the time of postmortem sampling (Supplementary Figs. S12 and S13), these significant associations suggest that at some point, immune infiltration had caused immunoediting.

### Identifying Factors Influencing Lesion-Specific Response

**Figure 5.**
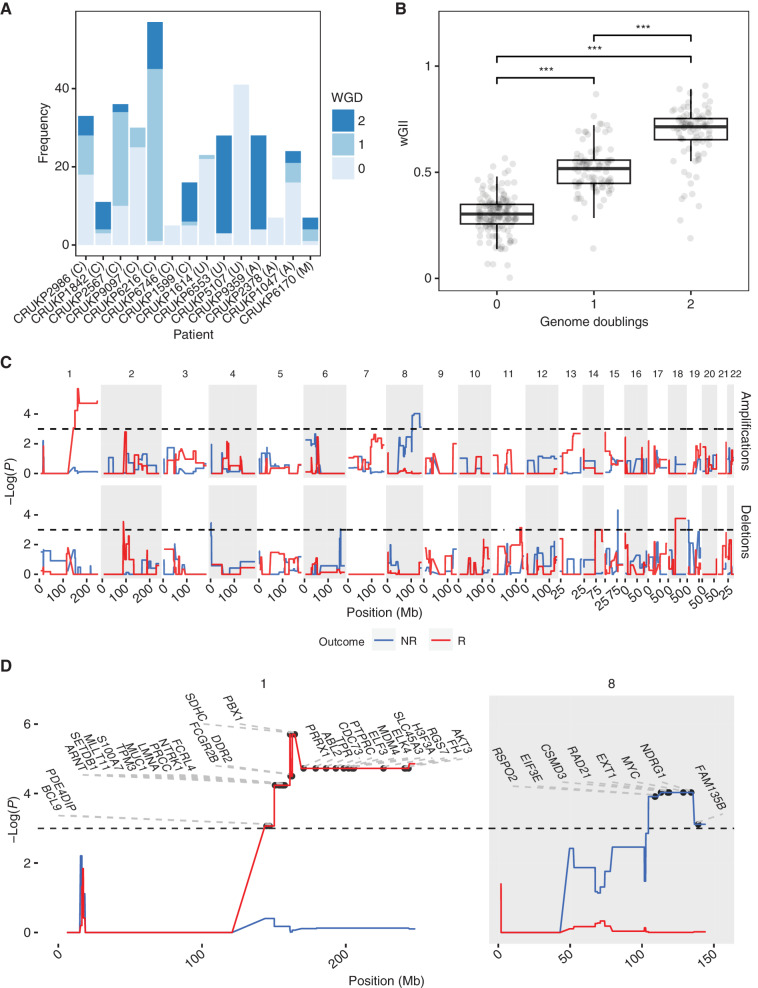
Tissue-level amplifications and deletions associated with response to ICI. A large proportion of samples underwent WGD (**A**), with successive WGD events associated with increasing wGII (**B**). Letters in brackets indicate melanoma subtype: A = acral, C = cutaneous, M = mucosal, U = melanoma of unknown primary. ***, *P* < 0.001. GISTIC permutation analysis (**C**) associated *MYC* amplification (chromosome 8q) with a nonresponsive phenotype, as well as chromosome 1 amplification with a responsive phenotype. Horizontal black dashed lines in top two panels of **C** indicate significance (*P* < 0.05). NR, nonresponse; R, response. **D,** Significant amplifications on chromosomes 1 and 8 from **C** with COSMIC genes labeled.

Dynamics of response to ICI are varied and include mixed responses, in which some tumors are regressing and others progressing (42). Given the diverse clonal evolutionary trajectories both within and between patients (Fig. 2A), including differential WGD status (Fig. 5A) and significantly increasing wGII with increasing WGD (Fig. 5B), we hypothesized that site-level responses to treatment might vary between lesions depending on their genetic constitution, whether in terms of genotype or copy-number alteration. To assess this, we matched radiologic response data based on RECIST criteria to panel sequencing data for each tumor, yielding an available dataset of 32 tumors from seven patients (mean ± SD 4.6 ± 3.3 tumors per patient; Supplementary Table S4). This limited subset can be attributed to the selection of patients who had ICI therapy as their last line of treatment before death, as well as our multiregional sampling design, as response data were at the lesion level. We performed a permutation-based GISTIC test to find regions that underwent significant copy-number alteration in responding versus nonresponding lesions.

Interestingly, copy-number gains of chromosome 8q containing COSMIC genes *RSPO2*, *EIF3E*, *CSMD3*, *RAD21*, *EXT1*, *MYC*, *NDRG1*, and *FAM135B* were significantly associated with a lack of response at the lesion level (Fig. 5C and D). *MYC* has previously been shown to promote an immune-suppressive stroma via cooperation with Ras (43). These results indicate that *MYC* may also be a marker of ICI resistance in metastatic melanoma. Conversely, a focal region on chromosome 1 (Fig. 5D) was significantly amplified in lesions that responded to ICI. Also of interest was the 9p21 locus (44, 45), which contains the prominent tumor suppressor *CDKN2A*, *MTAP*, as well as a cluster of *IFN* genes and has been linked to ICI resistance in pan-tumor studies (46). Loss of 9p21 was numerically more frequent in lesions with a lack of response, although this failed to reach significance in a Fisher exact test (*P* = 0.08). We also tested other loci commonly associated with therapeutic resistance in melanoma for an association between copy-number alterations and lesion-level response, including *BRAF, PTEN*, and *TP53;* however, these were all nonsignificant.

### Transcriptional Alterations Associated with Late-Stage Melanoma

To gain insight into the transcriptional changes associated with late-stage disease, we performed RNA-seq on bulk tumor samples. Tumor transcriptional profiles clustered first by patient and then by tissue site, indicating that tumor-specific factors determine expression profile over the location of the tumor in the body. Mean purity of samples for which RNA-seq was performed was 0.72 (Supplementary Fig. S14). Differential expression analyses between tumor and normal tissue revealed the MYC targets V2 hallmark gene set as most significantly upregulated in tumor samples (*q*-value < 0.0001; Supplementary Tables S5 and S6). Other significantly upregulated gene sets in tumor samples include those associated with cell division: G_2_–M checkpoint (*q*-value < 0.001), E2F targets (*q*-value < 0.001), and mitotic spindle (*q*-value < 0.01).

We also investigated whether increases in ploidy were associated with transcriptional changes. Differential expression analyses revealed that hallmark gene sets mitotic spindle (*q*-value < 0.005) and G_2_–M checkpoint (*q*-value < 0.05) were significantly upregulated with increasing ploidy, although for mitotic spindle, purity was also a significant factor (*q*-value < 0.01).

To assess the composition of immune cells present in tumor samples, we applied consensusTME (47) to the transcriptional data from our cohort together with The Cancer Genome Atlas (TCGA) melanoma samples. Mean normalized enrichment scores (NES) were mostly negative for all cell types and lower than in the TCGA samples (Supplementary Fig. S15A), whereas treated TCGA samples showed intermediate immune scores (Supplementary Fig. S15B). Tumor Immune Dysfunction and Exclusion (TIDE) scores (48) predicted more than 98% of our samples as nonresponders to ICI. Specifically, our cohort had positive immune exclusion and negative immune dysfunction scores, in agreement with the cold microenvironment observed histologically (most samples had minimal tumor-infiltrating lymphocytes; Supplementary Fig. S12). We did, however, observe positive NES for M2 macrophages in 29.2% of samples across 11 of 14 patients, which are known to promote tumorigenesis (49), although it should be noted that macrophage classification is more complex than the M1/M2 paradigm (50). We hypothesized that as MYC promotes macrophage recruitment through emission of the CCL9 chemokine (44), MYC copy-number amplification might be associated with M2 NES score. This association was, however, nonsignificant using both raw and purity-corrected consensusTME NES scores (Pearson correlation). Further comparison between M2 NES and ploidy was also nonsignificant.

A comparison of brain and extracranial metastases revealed no significant differences in immune infiltration, exclusion, and dysfunction scores. However, we observed higher scores of melanocytic plasticity signature (MPS; ref. 51) in brain metastases (Mann–Whitney *U* test *P* = 0.00014), suggesting a less differentiated phenotype (Supplementary Fig. S16A). We further evaluated the effect of the metastatic site on pathways found to be differentially expressed in brain metastases in previous studies (52–57). No difference in the expression of OXPHOS genes across sites was found when controlling for purity, although brain metastases were significantly associated with the enrichment of the biosynthesis of unsaturated fatty acids (Supplementary Fig. S16B).

Another question of interest was the influence of local copy-number changes on transcription, either of local genes or genes at other loci via transcription factors. In a pairwise analysis of all genes, the most significant correlation was a self-association involving *PHF3* on chromosome 6 (Supplementary Fig. S17). Patient CRUKP2986 had a clonal, focal copy number increased from a copy number of 2 to 15 at this locus, with corresponding increases in expression compared with other patients, suggesting that this change was under selection. PHF3 has been associated with a variety of functions, including UV-induced DNA damage response (58) and neuronal differentiation (59). PHF3 is also significantly amplified in the TCGA cutaneous melanoma cohort. Interestingly, CRUKP2986 was a case of a non-UV–exposed melanoma, suggesting a potential alternative function for PHF3 here.

To assess the potential impact of chromosomal rearrangements on protein-coding genes, we inferred putative gene fusions from the RNA-seq data. We found a mean of 10 gene fusions per patient (range, 2–20), 90.8% of which were subclonal. In seven cases the fusions involved an oncogene or tumor suppressor gene, although we observed no *BRAF* fusions in this cohort (Supplementary Fig. S18A and S18B).

### Investigating Copy-Number Changes at Single-Cell Resolution

In our analysis of ploidy by FISH, we often observed cell-to-cell variation in ploidy within the same tumor sample (Supplementary Fig. S19). To investigate this further, we used a single-cell whole-genome sequencing (scWGS) approach. Importantly, our particular method did not include a whole-genome preamplification step, allowing the reliable generation of high-resolution single-cell copy-number profiles (60). We performed scWGS in one metastatic tumor from each case (*n* = 50 cells per sample) and included four normal samples as a control.

Overall, the inferred mean ploidies from the scWGS data were largely concordant with our estimates using bulk sequencing and FISH (Supplementary Fig. S20), suggesting most cells in the sample were a part of the same clonal expansion. In three patients, CRUKP2567, CRUKP1614, and CRUKP9359 (bulk sequencing–estimated ploidies of 3n, 2n, and 5n, respectively), we identified two distinct populations (i.e., low and high ploidy) via fluorescence-activated cell sorting (FACS; Supplementary Fig. S21) and performed scWGS on both. The lower ploidy samples in CRUKP1614 and CRUKP9359 did not contain cancerous cells (Supplementary Fig. S22), as indicated by their low wGII values, suggesting homogeneous ploidy in these metastases.

**Figure 6.**
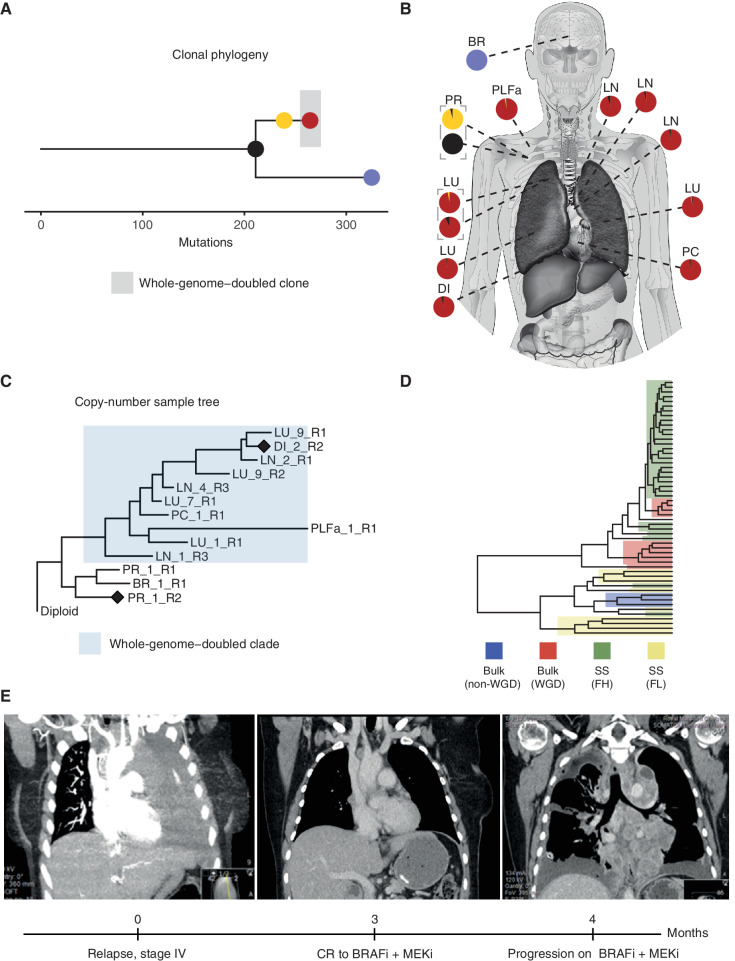
Identification of a likely non–whole-genome–doubled clone that was not identifiable from bulk sequencing data in CRUKP2567. Clonal phylogeny of CRUKP2567 (**A**), with anatomic diagram **(B)** based on bulk SNVs mapping samples to clones on the tree. The scale indicates the number of mutations. **C,** MEDICC2 copy-number tree for bulk exome samples from CRUKP2567. The cluster highlighted in blue has undergone one WGD event, while the other nonhighlighted cluster, containing brain metastasis and primary tumor samples, has not. Diamonds indicate the samples for which bulk copy-number profiles are displayed in Supplementary Fig. S23. **D,** Hierarchical clustering tree containing all single cells (SS) from FACs-high-ploidy sorting (FH) and FACs-low-ploidy sorting (FL), as well as WGD bulk samples and non-WGD bulk samples. **E,** Radiologic images of the patient indicating thorax upon initiation of stage IV disease and complete extracranial response to BRAF inhibitor, followed by rapid recolonization of the thorax with resistant clones (left to right). BR, brain; BRAFi, BRAF inhibitor; CR, complete response; DI, diaphragm; LN, lymph node; LU, lung; MEKi, MEK inhibitor; PC, pericardium, PLFa, pleural fluid (archival); PR, primary.

CRUKP2567, on the other hand, was exceptional (clonal phylogeny shown in Fig. 6A) in that the lower ploidy population in the diaphragmatic metastasis (Fig. 6B) was reflective of malignant cells, indicated by their high wGII values. Further inspection of the bulk sequencing copy-number tree for this patient revealed a WGD event in the branch leading to the majority of metastases but not detected in the primary tumor or brain metastasis (Fig. 6C). Specifically, in the bulk copy-number profile primary sample (ploidy ∼2), we observed five copies (5n) of chromosome 5p (Supplementary Fig. S23A), which rose to 7n in the WGD diaphragmatic metastasis (ploidy ∼ 3). In the single-cell copy-number profile of the diaphragmatic metastasis, we observed both cells with 7n (from the high-ploidy FACS population) and cells with 4n to 5n (from the lower-ploidy FACS population, with ploidies ranging from 1–2; Supplementary Fig. S23B). Overall, this suggests the presence of two coexisting clones in the diaphragmatic metastasis: one WGD and one non-WGD clone. To assess this further, we performed hierarchical clustering of copy-number profiles from the bulk samples and nonnormal single cells from this patient (Fig. 6D). The resulting tree contained two primary clusters, one containing the bulk samples that had undergone WGD as well as the high-ploidy FACS single cells, and the other containing the bulk samples lacking WGD as well as the low-ploidy FACS single cells.

In terms of the clinical course of CRUKP2567, at stage IV diagnosis, this patient had extensive disease in the thoracic cavity, followed by complete extracranial response to combined BRAF and MEK inhibitor treatment, and subsequent rapid reemergence of thoracic disease that was resistant to targeted treatment (Fig. 6E). One of the pretreatment pleural fluid samples exhibited evidence of WGD, suggesting that clones existed with WGD both prior to and after treatment. The presence of two clones of differing WGD status in the diaphragmatic metastasis of CRUKP2567 raises the possibility that the lower-ploidy non-WGD clone originates from the non-WGD pretreatment population, and that the WGD clone repopulated this site after acquisition of resistance.

## DISCUSSION

Our study expands on previous multilesional metastatic melanoma analyses (8–11, 61) by increasing the number of patients studied, the number of samples per patient, and the breadth of represented sites of metastases, as well as the breadth of multiomic data generated. We observe a number of different evolutionary routes to lethality. Notable among these is the presence of WGD, which is clonal in four patients, subclonal in seven patients, and absent in three patients. This contrasts with previous reports, in which near “universal tetraploidization” was observed in distant metastases (11). One caveat of extensive multilesional sampling is that patient numbers are often small; as more work is performed, the extent of the influence of sampling bias will become clear. Predominantly single-lesion analyses (6) have derived a figure of 40% for the rate of WGD in advanced melanoma, although, of course, the caveat here is that WGD may be subclonal and therefore missed in some patients. Likely, the truth lies somewhere between these two extremes. The differential status of WGD between patients was found in both cutaneous and acral melanomas, indicating that tumors use WGD to sculpt a select number of critical genes rather than being driven by the excess mutations in sun-damaged cutaneous melanomas. Copy-number events found in WGD tumors were mainly losses, indicative of a mechanism to buffer against the ratchet-like accumulation of deleterious alterations, as illustrated by previous simulatory work (62).

Examination of tumor characteristics of specific sites led us to the observation that late-emerging brain metastases display unique properties compared with other sites, having lower wGII and often diverging early in the clonal phylogeny. This is reminiscent of our observations of pancreatic metastases from clear-cell renal cell carcinoma, which also emerged from ancestral clones and grew slowly (16). We found that a chemotherapy mutational signature was lacking in the brain metastasis of patient CRUKP5107. Given that these chemotherapies target dividing cells, the lack of evidence in the brain suggests either lack of exposure (due to the blood–brain barrier) or a slow-cycling population of tumor cells in the brain. However, clinical manifestations of brain metastases in melanoma are varied, so it is unlikely that all brain metastases follow this path.

As was expected, we observed the acquisition of resistance to both targeted therapies (30) and ICIs (39, 63, 64). The former primarily involved secondary driver mutations in the MAPK pathway. The consistency of acquired resistance to targeted therapy in our cohort suggests that these treatments may have little effect on late-stage, ICI-exposed melanoma. Resistance to ICIs involved copy-number changes and LOH events in antigen-presentation pathway genes, such as *B2M* and *JAK2*. Losses and mutations in these genes have been linked to primary (63) and acquired resistance to PD-1 blockade in melanoma (64), suggesting that melanoma cells become insensitive to the antiproliferative effects of IFNγ (via JAK1/2 alterations) or lose antigen presentation (via *B2M* alterations). Examination of within-patient heterogeneity of antigen-presentation alterations revealed that these were for the most part present clonally across all tumors, which is in contrast to data from targeted MAPK inhibition therapies (35, 37), suggestive of an early acquisition of these alterations. In addition, we observed a lack of solid immunoediting signal, suggesting that neoantigen burden was not particularly important in these patients and is trumped by antigen-presentation pathway changes. It must be noted, however, that the technical limitations of neoantigen calling from DNA sequence data may have also influenced this analysis; neoantigens are difficult to predict, and it might be the case that small numbers of true strong neoantigens were downregulated or lost that were not identified here. Other potential influences that could explain the lack of immunoediting signals include tumor microenvironmental factors such as immunoregulatory cell lineages, including M2 macrophages (65) and regulatory T cells (66). However, we did not study these in detail here.

Increasing the resolution of this analysis of treatment resistance in relation to individual lesion radiologic response, we found several genes of interest that may influence response to ICIs, including *MYC*, known to promote an immunosuppressive stroma (43). It should be noted, however, that functional validation of this finding in melanoma models was beyond the scope of this work. Although *MYC* is an essential modulator of tumor growth via both tumor–cell intrinsic mechanisms and its influence on the tumor microenvironment, and immune effectors (43, 67), direct targeting of *MYC* is not feasible (68). Instead, small-molecule inhibitors that target cofactors of *MYC* (69), such as histone deacetylase genes, have been developed and are used in the clinic to treat a variety of cancers (70). Taken together, these findings suggest that the combination of ICIs with a form of MYC inhibition could be a potential therapeutic avenue in ICI-refractory melanoma. It will also be of interest to assess 9p21 loss in larger cohorts, as this event was more frequent in lesions with a lack of response but not statistically significant.

Much of the existing postmortem melanoma research has been limited to bulk DNA sequencing data (8, 10, 11). The inclusion of additional data modalities, namely, transcriptomics and single-cell sequencing data in our study, revealed novel findings with clinical and technical implications. PHF3 has previously been associated with UV DNA damage response in melanoma (58); however, here, it appears to be a clonal driver of a non–sun-exposed melanoma, suggesting a potential alternative role for *PHF3* in melanoma progression, warranting further investigation in larger cohorts. It should be noted that differing purity of RNA-seq samples has the potential to influence transcriptomic analyses, although in our cohort we observed relatively high purity of samples measured using WES and did not observe specific biases in purity toward particular patients (Supplementary Fig. S24) or tissue sites (Supplementary Fig. S25). Polyclonal seeding is typically thought of in terms of mutations; bulk sequencing data and the infinite sites model allow clonal reconstruction from the pooled sequencing data of many cells (71). Reconstruction of subclonal copy-number profiles, however, remains challenging. Our single-cell data reveal a case of polyclonal seeding at the level of WGD, suggesting that sample-level trees produced with tools such as MEDICC2 (27) could underestimate intratumor heterogeneity at the copy-number level.

In this study, we have emphasized extensive tumor sampling over patient numbers. Consequently, one of the limitations we face is low statistical power when using the patient as the unit of statistical inference. Other considerations include sampling time; as our samples are taken at postmortem, we may miss selective events that occur during the patient's life. Patients were also subject to multiple lines of therapy, the influences of which may be difficult to disentangle. Primary tumor samples were not available for all patients, reflecting the challenges of sampling small cutaneous lesions. Nonetheless, this detailed multilesional study has elucidated various important themes in advanced melanoma, shedding light on the topic of WGD, mechanisms of resistance, polyclonal seeding at the level of copy number, and more. In terms of clinical implication, the lack of response to ICIs in these late-stage patients supports an emphasis on neoadjuvant ICI trials, and tumor cell–intrinsic mechanisms of therapy may be required in the future.

## METHODS

### Sample Procurement and Processing

The PEACE study is a pan-cancer, UK-wide research autopsy program (NCT03004755) designed to comprehensively evaluate the biology of metastatic disease and drug resistance. A list of the consortium members can be found in Supplementary Table S1. The study is sponsored by the University College London Clinical Trials Unit. Inclusion criteria include patients with advanced cancer. Written informed consent was provided by patients during life or by next of kin after death. The study was approved by the Health Research Authority National Research Ethics Service Committee ­London–Dulwich on the August 15, 2013, in accordance with the Human Tissue Act 2004 of the Parliament of the United Kingdom, with Research Ethics Committee reference 13/LO/0972. Postmortems are referred to as “tissue harvests” (TH) within this study. These were conducted as soon as possible following death [median 52 hours, range, 23–144; median time to refrigeration (TTR) 7 hours, range, 1–13], at either the University College London Hospital (UCLH) or Guy's and St Thomas’ NHS Foundation Trust mortuaries. The postmortem interval (PMI) and TTR were noted for each case. At TH, sampling of metastases was led by a pathologist, and multiple regions of individual metastases were procured where size permitted. Individual tumor regions were bisected along the long axis, with one half immediately snap-frozen in liquid nitrogen before long-term storage at −80°C and the other half fixed in 10% neutral buffered formalin prior to embedding in paraffin blocks and stored at room temperature. Fresh instruments were used for handling each individual tumor region to avoid cross-contamination. Body cavity fluid (pleural, abdominal, and cerebrospinal) was also collected, and following centrifugation, cell pellets were isolated from supernatant and snap-frozen in liquid nitrogen. Where possible, peripheral blood was collected in life (at the time of consent) and processed to separate buffy coat and plasma. Where no blood was procured in life, blood was collected at TH by the pathologist by performing a ventricular stab.

### DNA and RNA Extraction from Frozen Tissue, FFPE Tissue, and Blood

DNA and RNA were copurified using the AllPrep DNA/RNA Mini Kit (Qiagen). Briefly, a 2-mm^3^ piece of tissue was added to 900 μL of lysis buffer and homogenized for 5 seconds using the TissueRaptor (Qiagen), with a fresh homogenization probe being used for each preparation. Each lysate was applied to a QiaShredder (Qiagen) and then sequentially purified using the DNA and RNA columns according to the manufacturer's protocol. Germline control DNA was isolated from whole blood using the DNeasy Blood and Tissue Kit (Qiagen) according to the manufacturer's protocol. Archival FFPE tissue specimens were first evaluated by a histopathologist from hematoxylin and eosin slides to identify tumor-rich areas and macrodissected from the FFPE block. The Qiagen GeneRead FFPE DNA extraction kit was used for DNA purification according to the manufacturer's protocol.

### Quality Control of DNA and RNA Samples

For each postmortem case, a selection of samples of DNA and RNA (from fresh-frozen tissues) was run on 1.2% agarose gels to review their quality. For DNA samples, routine TapeStation analysis was performed for DNA integrity assessment (DIN score) for 11 of the 14 cases. For the other cases, library preparation was undertaken at a different institution (UCLH) for CRUKP2986, and for CRUKP1842 and CRUKP6170, these were prepared prior to this operating procedure being in place. RNA samples selected for sequencing were also assessed for integrity on the Agilent bioanalyzer to give an integrity score (RIN). Spearman correlation coefficients were calculated between the DIN and RIN scores and the PMI and TTR. DNA integrity as measured by the DIN score was collated for 125 of 153 samples that were subjected to WES (82%) and correlated with both the PMI and TTR where data were available. The 28 missing samples (18%) were derived from the first three TH conducted at the start of the study (CRUKP2986, CRUKP1842, and CRUKP6170) before DINs were routinely recorded and DNA stock was exhausted. Overall, the majority of samples had a DIN greater than 6 (107/125, 86%).

Evaluation of RNA integrity as measured by the RIN score was undertaken for tumor samples included in the RNA-seq analysis (*n* = 111 with available information). We observed a wide range of RIN scores from 0.0 to 8.2, with a median of 4.3. A RIN >6 was noted in 12%. There was a significant but weak negative correlation with the PMI (*r* = −0.23, *P* 0.02). No trend was observed between the RIN and TTR.

### Targeted Panel Design and Validation

To facilitate the sequencing of large numbers of samples, we developed a custom melanoma gene panel. First, we performed a comprehensive review of the literature for melanoma driver genes (4, 5, 72–76) and selected genes involved in treatment resistance for both BRAF/MEK-targeted therapy and immune-checkpoint therapies (30, 35, 38, 64, 77–82) as well as DNA repair genes (83). We also included the 20 most common genes mutated in melanoma as reported in COSMIC (84) and MutSig, as well as a preexisting list of immune genes included in a renal panel (13). Genes carrying variants of germline interest that may be involved in immune-related toxicity were also included (Supplementary Table S7). A single-nucleotide polymorphism (SNP) backbone was included to improve the accuracy of copy-number calling. The total size of the panel was 1.9 Mb. We validated the panel design by ensuring sufficient coverage of all target regions and by comparing the SNV/indel and copy-number calls from samples that were sequenced with both panel and WES. Out of all mutations called in the exome samples (see details below), 91.9% were also called in the panel data, and the copy-number calls were consistent between the exome and panel in more than 93% of segments.

### Whole Exome and Custom Panel Library Construction and Sequencing

Depending on the available yield, genomic DNA samples were normalized to either 1 to 3 μg or 200 ng for the Agilent SureSelectXT Target Enrichment Library Protocol (Agilent Technologies), standard- or low-input sample preparation, respectively. Samples were sheared to 150 to 200 bp using either a Covaris E220 or LE220-plus (Covaris). For samples sheared using the E220 instrument, the run parameters outlined in the Agilent SureSelectXT protocols were followed; for those sheared on the LE220-plus, the following optimized parameters were used: 36 iterations of sonication for 10 seconds with 30% duty factor, 450 peak incident power and 200 cycles per burst, at 4°C to 8°C.

Library construction of samples was then performed following the SureSelectXT protocols, using 6 precapture PCR cycles for the standard-input samples and 10 precapture PCR cycles for the 200-ng low-input samples. Hybridization and capture were performed for each individual sample using an Agilent custom target-specific capture library, Melanoma Driver Panel (version 2). Captured libraries were amplified and indexed using 13 postcapture PCR cycles in PCR reactions, which included 1 of 96 unique single indexes. The quality and fragment size distributions of the purified libraries were assessed using the Agilent TapeStation High-Sensitivity D1000 Assay on a 4200 TapeStation Instrument (Agilent Technologies). Amplified, captured, and indexed libraries passing this quality control step were normalized to 2 nmol/L and pooled for sequencing, ensuring that unique indexes were allocated to all libraries in a pool. The quality and fragment size distributions of the library pool were assessed using the Agilent TapeStation High-Sensitivity D1000 Assay and quantified using the Qubit dsDNA HS Assay (Thermo Fisher Scientific). These quality control results were used to denature and dilute the pool in preparation for sequencing on the Illumina NextSeq 500 sequencing platform. The final libraries were sequenced with 150 bp paired-end reads on the NextSeq 500 at the Advanced Sequencing Facility at The Francis Crick Institute. Target coverage was 500× for tumor regions and associated normal tissue.

A subset of patient samples were nominated for WES to maximize the types of metastatic sites represented in the WES analysis. Genomic DNA isolated from each sample was normalized to 1 to 3 μg. Libraries were prepared using the Agilent SureSelectXT Human All Exon v5 enrichment capture library. The libraries were prepared using 6 precapture and 12 postcapture PCR cycles. The quality and fragment size distributions of the purified libraries were assessed using the Agilent TapeStation High-Sensitivity D1000 Assay (Agilent Technologies). Captured whole-exome libraries passing this quality control step were normalized to 2 nmol/L and pooled for sequencing on the HiSeq 4000 sequencing platform. The final libraries were sequenced with 100 bp paired-end reads on the HiSeq 4000 at the Advanced Sequencing Facility at The Francis Crick Institute. Target coverage was 250× for tumor regions and the associated normal tissue.

### Library Construction from Genomic DNA Extracted from FFPE Tissue for Sequencing

For DNA extracted from FFPE tissue, adapter-ligated libraries were prepared using the KAPA HyperPrep Kit (KAPA Biosystems), followed by Agilent SureSelectXT capture enrichment according to the manufacturer's protocols. Samples were normalized to 400 ng and sheared to 150 to 200 bp using a Covaris E220 (Covaris), following the parameters outlined in the KAPA HyperPrep Kit for SureSelect Target Enrichment protocol. KAPA HyperPrep libraries were generated and amplified using 10, 11, or 12 precapture PCR cycles and subsequently enriched using either the Agilent custom Melanoma Driver Panel or SureSelectXT Human All Exon v5 capture library. The quality and fragment size distributions of the purified libraries were assessed using the Agilent TapeStation High-Sensitivity D1000 Assay (Agilent Technologies).

### RNA-seq Library Construction and Sequencing

Libraries for RNA-seq were constructed using the KAPA RNA HyperPrep RNA Kit with RiboErase (HMR; for Illumina; KAPA Biosystems) according to the manufacturer's protocol. Samples were specifically depleted for both cytoplasmic (5S, 5.8S, 18S, and 28S) and mitochondrial (12S and 16S) rRNA species. RNA samples were normalized to 50 to 70 ng and fragmented to 200 to 300 bp fragments for library construction. Libraries were indexed with unique KAPA Dual-Indexed Adapters (KAPA Biosystems) and PCR amplified using 15 or 16 PCR cycles. The quality and fragment size distributions of the purified libraries were assessed on a 4200 TapeStation Instrument (Agilent Technologies). Libraries passing this quality control step were normalized and pooled for sequencing on the HiSeq 4000 sequencing platform. The final libraries were sequenced with 100 bp paired-end reads, to a target depth of 50 million reads per sample, on the HiSeq 4000 at the Advanced Sequencing Facility at The Francis Crick Institute.

### Single-Nuclei Preparation, Sorting, and Sequencing

Single-nuclei sequencing was performed following the protocol in ref. 61 (Chapter 15). In short, nuclei were isolated from frozen tissue and stained with Hoechst and propidium iodide, and then populations of different ploidy were gated in FACS. From each population, 48 cells were processed through library preparation and single-end DNA sequencing.

### Somatic SNV and Indel Calling from Multiregion WES and Panel Sequencing

Paired-end reads (2 × 100 bp for WES, 2 × 150 bp for panel sequencing) in FastQ format sequenced by Hiseq or NextSeq were aligned to the human reference genome (build hg19) using the Burrows–Wheeler Aligner (BWA) v0.7.15 with seed recurrences (-c flag) set to 10,000 (1). Intermediate processing of Sam/Bam files was performed using Samtools v1.3.1, and deduplication was performed using Picard 1.81 (http://broadinstitute.github.io/picard/). SNV calling was performed using Mutect v1.1.7 and small-scale indels were called running VarScan v2.4.1 in somatic mode with a minimum variant frequency (–min-var-freq) of 0.005 and a tumor purity estimate (–tumor-purity) of 0.75 and then validated using Scalpel v0.5.3 (scalpel-discovery in –somatic mode; intersection between two callers taken; refs. 2–4). SNVs called by Mutect were further filtered using the following criteria: (i) variants falling outside the targeted capture range (±50 bp padding) or into mitochondrial chromosome, haplotype chromosome, HLA genes, or any intergenic region were not considered; (ii) presence of both forward and reverse strand reads supporting the variant; (iii) >5 reads supporting the variant in at least one fresh-frozen tumor region of a patient; (iv) variants were required to have a variant allele frequency (VAF) of 0.2 in at least one fresh-frozen tumor region; and (v) sequencing depth needed to be ≥20 and ≤3,000 across all fresh-frozen tumor regions. Mutations called in FFPE samples were restricted to variants that passed this additional filtering in fresh-frozen samples. Dinucleotide substitutions (DNV) were identified when two adjacent SNVs were called, and their VAFs were consistently balanced (based on the proportion test, *P* ≥ 0.05). In such cases, the start and stop positions were corrected to represent a DNV, and frequency-related values were recalculated to represent the mean of the SNVs. Variants were annotated using Annovar (5). Individual tumor biopsy regions were judged to have failed quality control and excluded from analysis based on the following criteria: (i) sequencing coverage depth below 100× and (ii) low tumor purity such that copy-number calling failed.

### Germline Variant Calling

SNPs were called in the germline sample using Platypus v0.8.1 with default parameters apart from –genIndels = 0 and –minMapQual = 40, and calls were restricted to the targeted capture range (±50 bp padding). Tumor regions were genotyped based on the variants identified in the germline (parameters set to –minPosterior = 0 –getVariantsFromBAMs = 0). SNPs with a minimum coverage of 50× in the germline and the tumor sample were used for allele-specific copy-number segmentation.

### Purity, Ploidy, and Copy-Number Analyses

CNVkit v0.7.3 was used with default parameters on paired tumor–normal sequencing data (6). Outliers of the derived log_2_-ratio (logR) calls from CNVkit were detected and modified using Median Absolute Deviation Winsorization before case-specific joint segmentation of fresh-frozen samples to identify genomic segments of constant logR (7). FFPE samples were segmented separately, leveraging the segment information from the fresh-frozen samples.

Tumor sample purity, average ploidy, and absolute allelic copy number per segment were estimated using ABSOLUTE v1.2 in allelic mode (10). In line with recommended best practice, exome ABSOLUTE solutions were reviewed by three bioinformaticians, with solutions selected based on the majority vote. We implemented an automated solution selection for the panel sequencing samples based on the algorithm used in the manual selection. In short, we prioritized solutions with a better fit of somatic SNV multiplicity, whose clonal VAF peak matched half the purity and whose proportion of subclonal copy-number segments along the genome was lower than 50%. We weighted the scores assigned to these criteria based on their variance on different solutions for the same sample, allowing us to select samples based on the most meaningful differences.

Copy-number alterations were then called as losses or gains relative to overall sample-wide estimated ploidy. Arm-level gains and losses were called if ≥50% of the arm was gained or lost. Driver cytoband copy number was identified by overlapping called somatic copy-number segments with putative driver copy-number regions previously identified by GISTIC2 analysis of TCGA melanoma data. Allele-specific segmentation was performed using the paired PSCBS method after removal of single-locus outliers (R package PSCBS v0.61.0). The frequency of events across the cohort was calculated after cohort-level minimum consistency segmentation, dividing segments so that all samples have common edges.

The wGII was calculated for every sample as the average proportion of the genome with aberrant copy number, weighted on each of the 22 autosomal chromosomes.

To evaluate the effect metastatic sites have on wGII across the cohort, we compared the mean wGII of each site to a site-specific null distribution. For each site, the null distribution consisted of the mean wGII of 10,000 sets of samples containing the same number of samples per patient as each specific site but including samples from any metastatic site. This way, the patient effect on the mean wGII is included in the null distribution while randomizing the metastatic site effect. Additionally, we tested the effect of metastatic sites on wGII with a linear mixed-effects model.

MSAI was called when samples from the same case had regions of AI with a different parental allele lost/gained. MSAI segments were identified by annotating the minor allele (“ref” or “alt”) at every SNP overlapping the segment. SNPs with allele frequencies between 0.45 and 0.55 were excluded to avoid sequencing sampling errors. Segments were called MSAI when they had at least three overlapping SNPs and >80% of SNPs were discordant between a pair of samples. Pairwise Manhattan distances were calculated for each of these segments, and sample status for each segment was assigned with two-means clustering.

To conservatively time mutations with respect to WGD, we calculated the multiplicity of each mutation. Mutations with multiplicity equal to the major copy number of the genomic region in a WGD sample were called pre-WGD, whereas mutations with multiplicity lower than the major and minor copy number were called post-WGD. The timing of other mutations was not called. Parallel WGD events were called when pre-WGD mutations were not shared between samples.

### FISH

Upon noticing high-ploidy (up to 7n) estimates in some samples, we undertook FISH on a selection of samples as an orthogonal measure. Sections (4 μm) were cut from corresponding FFPE tissue blocks of at least two samples that had been submitted for WES, and a diploid control (normal spleen) was enumerated in the same manner. Following dewaxing and rehydration in ethanol, the sections were hybridized to chromosomes 2 and 15 centromere FISH probes (Abbott Molecular/Vysis, labeled with SpectrumFreen and SpectrumOrange flurophores, respectively) using the Dako Histology FISH Accessory Kit (Dako; K5799) according to the manufacturer's instructions. Image acquisition was performed with a confocal microscope (Zeiss Invert880 with Airyscan). Z-stack images of single nuclei were acquired and imported into Fiji for further analysis. Manual evaluation of centromeric probes was performed and in at least 300 nuclei with nonoverlapping borders taken from >3 spatially distinct “tiles” per slide image.

Ploidy estimates were highly significantly positively correlated between FISH and exome (Pearson test, *r* = 0.7, *P* < 0.0001; Supplementary Figs. S19 and S20), although the modal ploidy of FISH tended to underestimate exome ploidy at higher ploidies (e.g., >3n). This is most likely due to a combination of the use of interphase FISH rather than metaphase FISH, which was necessary but is technically more challenging—for example, due to lack of melanoma-specific probes and resulting contamination from normal cells, as well as cell-to-cell heterogeneity in ploidy. Nonetheless, at these higher ploidies, the range of ploidies observed with FISH encompassed the exome estimated ploidy for the majority of cases, suggesting that this was a genuine signal.

### Subclonal Reconstruction

In order to estimate whether mutations were clonal or subclonal, and the clonal structure of each tumor, a modified version of PyClone was used. For each mutation, two values were calculated, obsCCF and phyloCCF. obsCCF corresponds to the observed cancer cell fraction (CCF) of each mutation. Conversely, phyloCCF corresponds to the phylogenetic CCF of a mutation. To clarify the difference between these two values, consider a mutation present in every cancer cell within a tumor. A subclonal copy-number event in one tumor region may lead to loss of this mutation in a subset of cancer cells. Although the obsCCF of this mutation is below 1, from a phylogenetic perspective, the mutation can be considered “clonal,” as it occurred on the trunk of the tumor's phylogenetic tree, and, as such, the phyloCCF may be 1. To calculate the obsCCF of each mutation, local copy number (obtained from ABSOLUTE), tumor purity (also obtained from ABSOLUTE), and VAF were integrated. In brief, for a given mutation, we first calculated the observed mutation copy number, *n*_mut_, describing the fraction of tumor cells carrying a given mutation multiplied by the number of chromosomal copies at that locus using the following formula:




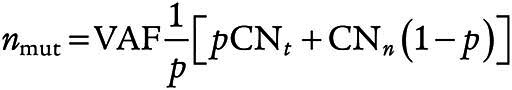




where VAF corresponds to the VAF at the mutated base, and *p*, CN_t_, and CN_n_ are respectively the tumor purity, the tumor locus-specific copy number, and the normal locus-specific copy number (CN_n_ was assumed to be 2 for autosomal chromosomes). We then calculated the expected mutation copy number, *n*_chr_, using the VAF and assigning a mutation to one of the possible local copy-number states using maximum likelihood. In this case, only the integer copy numbers were considered.

All mutations were then clustered using the PyClone Dirichlet process clustering (85). PyClone version 0.13.1 was used for two cases (CRUKP6216 and CRUKP1599 due to the exceptionally high TMB in these cases), whereas the remaining cases were analyzed with PyClone version 0.12.3. For each mutation, the observed variant count was used and reference count was set such that the VAF was equal to half the preclustering CCF. Given that copy number and purity had already been corrected, we set the major allele copy numbers to 2, minor allele copy numbers to 0, and purity to 0.5, allowing clustering to simply group clonal and subclonal mutations based on their preclustering CCF estimates. We ran PyClone with 10,000 iterations and a burn-in of 1,000, and default parameters, with the exception of –var_prior set to “BB” and –ref_prior set to “normal.”

To determine the phyloCCF of each mutation, a similar procedure to that described above was implemented, with the exception that mutations were corrected for subclonal copy-number events. Specifically, if the observed VAF was significantly different from that expected (*P* < 0.01, using prop.test in R) given a clonal mutation, we determined whether a subclonal copy-number event could result in a nonsignificant (*P* > 0.01) difference between observed and expected VAFs. The preclustering CCF for each mutation was then calculated by dividing *n*_mut_ by *n*_chr_. Subclonal copy-number events were estimated using the raw values from ABSOLUTE output. Finally, to ensure that potentially unreliable VAFs of indels did not lead to separate mutation clusters, each estimated indel CCF was multiplied by a region-specific correction factor. Assuming the majority of ubiquitous mutations, present in all regions, are clonal, the region-specific correction factor was calculated by dividing the median mutation CCF of ubiquitous mutations by the median indel CCF of ubiquitous indels.

### Identification of Subclonal Mutations Driven by Copy-Number Loss

Mutations were investigated in order to identify those whose absence, or low CCF values, may be driven by copy-number loss events. For each tumor, we identified any SNV residing in genomic segments of copy-number heterogeneity across tumor regions, with minor and major copy-number aberrations considered separately. For each chromosome, we grouped mutations into noncontiguous genomic segments with consistent copy-number states within tumor regions and within SNV clusters defined above. To restrict our analysis to mutations lost in at least one tumor region, we determined the median CCF value of each SNV group and only considered SNV groups in which the median CCF value was ≤0.25 in at least one tumor region. We then evaluated whether copy-number loss coincided with lower CCF levels using a one-sided Wilcoxon test or, if more than two copy-number states were present across tumor regions, a one-sided Cochrane–Armitage trend test. To ensure the lower CCF value was driven by copy number and not tumor region, we also implemented a regression analysis, including both copy number and region in the model. If more than 85% of mutations within a given PyClone cluster were determined to be driven by copy number, then the entire cluster was classified as copy-number driven. Finally, to avoid overestimating copy number–driven losses of mutations, only losses occurring in ≤75% of tumor regions were considered. In addition, comparisons were made between the results of each mutation's obsCCF and phyloCCF. Given that the only difference between the calculation of the two is that obsCCF does not correct for subclonal copy-number events, mutations that appear clonal by phyloCCF but subclonal by obsCFF may reflect copy number–driven heterogeneity. To avoid overestimating copy number–driven heterogeneity, only mutations with a VAF of at least 1% were considered potentially to reside on a subclonal copy number and thereby considered as potentially driven by subclonal copy-number loss.

### Phylogenetic Tree Construction

To ensure accurate tree construction, mutation clusters were first filtered to ensure no violation of evolutionary principles. In brief, two principles were considered. First, the pigeonhole principle, which states that two mutation clusters cannot be considered independent and on separate branches of an evolutionary tree if the sum of the cancer cell prevalence values of the two clusters exceeds 100% within a single tumor region. Second, a descendent clone must exhibit a smaller cellular prevalence than its ancestor within each and every tumor region, referred to as the “crossing rule.” Using these principles, it can be determined whether particular mutation clusters conflict with each other and cannot be fitted to the same evolutionary tree. For instance, if the cellular prevalence of mutation cluster 1 in tumor region 1 is 80% and the mean cellular prevalence of mutation cluster 2 in tumor region 1 is 60%, then, by the pigeonhole principle, cluster 2 must be a descendent of cluster 1. However, if in a different tumor region, the cellular prevalence of cluster 2 is greater than cluster 1, it can be said that clusters 1 and 2 conflict due to the “crossing rule.” To ensure accurate tree construction, only clusters with at least five mutations were included. For the majority of tumors, all subsequent clusters were used to manually construct a phylogenetic tree. However, for a subset of tumors, evolutionary conflicts were identified, and a small number of mutation clusters were therefore removed. In total, 65 of 177 mutation clusters were removed, 23 of these containing fewer than five mutations. This removed 528 mutations from a total 22,148 mutations, representing 2.4% of all clustered mutations. Topologies of manually constructed phylogenetic trees were verified with Pairtree (86). Ten of 14 trees had entirely consistent topologies between our manually constructed trees and the trees’ output by Pairtree; the four trees that were not exactly consistent had minor alterations to the topologies that did not affect any of the analyses (Supplementary Table S8).

Copy-number sample trees for bulk sequencing data were inferred using MEDICC2 (27).

Polyclonal seeding from primary to metastases was determined either by assessment of multiregional samples from the primary tumor or by inspection of the clonal phylogenetic trees and muta­tional signatures.

### Estimating Clonal Proportion from CCF Values for Anatomy Plotting

Anatomy plots are labeled with pie charts whose colors represent the clonal proportion of clones in a sample. As clone proportion is not an output of PyClone, it was necessary to create an algorithm that estimates clonal proportion jointly from the CCF values and the clonal tree topology for each patient. To do this, we developed a tree traversal algorithm as follows: start with the CCF of the basal node N1, CCFN1. If the sum of CCFs of all immediate children of N1 (CCFC1) is larger than CCFN1, scale CCFC1 to equal CCFN1. Subtract CCFC1 from CCFN1 to get the clonal proportion value of N1, CPN1. Repeat until all tips of the tree are reached.

### HLA Typing

HLA typing of patients, as well as identification of mutations in HLA genes, was performed with Polysolver v1.0.0.

### Neoantigen Prediction and Analysis of Immunoediting

Neoantigens were predicted from mutation data using netMHC v4.0 or netMHCpan v4.1 where this failed. For the immunoediting analysis, tests were performed at the level of the patient. A 2 × 2 contingency table was calculated for predicted neoantigens that look to have undergone loss, as well as nonsynonymous mutations that have undergone a loss. Loss of a neoantigen or mutation was defined relative to other samples from the same patient and was considered a loss if two or more other samples contained the neoantigen, while other samples had LOH and a lack of neoantigen/mutation call at the same locus.

For the analysis of immunoediting based on changes to the expression of neoantigenic loci, we used the same methodology as in ref. 87. Namely, the expression of neoantigens and mutations was defined in a binary manner, being expressed if there were more than four reads supporting the neoantigen in the RNA-seq data for a sample. Each tumor was considered separately in determining the overall counts for expressed versus nonexpressed neoantigens or mutations within a patient to account for changes in expression at the level of tumors. For example, for neoantigen x, tumors y and z could differentially alter the expression of x, even though x is at the same locus in both y and z.

### Mutational Signature Analysis

Mutational signatures were estimated using the deconstructSigs package in R (88). Subclone-specific mutational signature analysis was restricted to subclones with at least 50 mutations.

### RNA-seq Analyses

The sequencing data were analyzed using the nfcore/rnaseq pipeline (v3.0; ref. 89). First, the input FastQ files underwent quality evaluation with FastQC (0.11.9) and adapter trimming with TrimGalore! (0.6.6). Next, the reads were aligned to the human reference genome GRCh37 and quantified with RSEM (1.3.1). SAMtools (1.10) was used to sort the Bam files generated by the alignment and generate mapping statistics. featureCounts was used to summarize the mapped read distribution over genomic features and count overlaps with different classes of genomic features. Further quality control was performed by RSeQC (3.0.1) and Qualimap (2.2.2dev). MultiQC (v1.10.1) was used to check for artifacts introduced by contamination. Two out of the 162 samples tested (both normal samples) had very high percentages of unalignable reads and were removed from subsequent analyses. Additionally, three samples showed high percentages of mitochondrial ribosomal RNA (>1%), indicating possible bacterial contamination; these were also removed. Differential expression analyses between tumor and normal tissue were performed using VST-normalized expression values from DESeq2 (90), and using the geometric mean, these values within each hallmark gene set as input into a linear mixed-effects model, with tissue type (normal or tumor) and purity as fixed effects and patient as a random effect. For examining associations between expression of hallmark gene sets and the ploidy of samples, the geometric mean of VST-corrected expression values for genes in the gene set was taken, which was used as input to a linear mixed-effects model with ploidy and purity as predictors and patient as a random effect, accounting for grouping of patient samples.

### RNA Fusion Calling

To identify RNA fusions, nf-core (89) rnafusion pipeline version 2.1.0 was used. Briefly, RNA-seq reads were aligned to reference genome GRCh38 with STAR version 2.7.10a (91) to leverage the annotations of this build. STAR-Fusion version 1.10.1 (92) was used to detect fusions. Fusion calls were conservatively filtered by LeftBreakEntropy >0.3, SpanningFragCount >1, and fusion fragments per million (FFPM) >0.2. Fusions were called subclonal in each case when they were found in <80% of samples.

### Lesion-Specific Response to Treatment Analysis

Lesions were categorized as either progressing or not based on RECIST 1.1 criteria (93). A permutation-based GISTIC test was then performed to find regions that were significantly amplified or deleted in progressing versus nonprogressing lesions.

### Assessing KIT Amplification and Evidence for ecDNA Using FISH

*KIT* amplification was determined using KIT/Con4 FISH probes (Empire Genomics) labeled with Orange-dUTP and Green-dUTP, respectively. Four-micrometer cryosections were generated using a cryostat set at −20°C (BRIGHT) from tumor cryoblocks matching tumor regions in which genomic profiles showed *KIT* amplification. The sections were first air-dried for 30 minutes and then placed in a 3:1 methanol:acetic acid fixative for 5 minutes at room temperature. The sections were then hybridized with the FISH probes using the Dako Histology FISH Accessory Kit (Dako; K5799) according to the manufacturer's instructions. Image acquisition was performed with a confocal microscope (Zeiss Invert880 with Airyscan). Z-stack images of single nuclei were taken and imported into Fiji for further analysis.

### Data Availability Statement

Raw data are available under request on the European Genome-phenome Archive (EGA) repository under accession number EGAS00001007081. Processed data used in this study are available at https://doi.org/10.5281/zenodo.7673904. The source data and code used to generate the figures can be found in https://github.com/FrancisCrickInstitute/PEACE_melanoma_14_paper.

## Supplementary Material

Supplementary Tables S1-S8 S. Table 1: List of PEACE Consortium members. S. Table 2: Sample database used for the study for Panel, Exome and RNASeq samples. S. Table 3: Overview of lines of treatment given to each patient. S. Table 4: Lesions and patients included in the lesion-level response to ICI analysis. S. Table 5: Hallmark genesets significantly upregulated in normal tissue vs tumor tissue. Table shows p-values for tissue type, purity; T value for tissue type, purity; q-value for tissue type, purity (FDR corrected). S. Table 6: Hallmark genesets significantly upregulated in tumor tissue vs normal tissue. Table shows p-values for tissue type, purity; T value for tissue type, purity; q-value for tissue type, purity (FDR corrected). S. Table 7: Genes included in the target panel. S. Table 8: Tree topology comparisons between manually constructed trees and pairtree-constructed trees.

Supplementary Figures S1-S25 Supplementary figure 1: Cohort overview. Number of samples sequenced with whole exome, panel or whole RNA sequencing. Supplementary figure 2: Phylogeny and WGD events in CRUKP1047. Supplementary figure 3: Ploidy and SCNA burden. Supplementary figure 4: Overview of each case. Supplementary figure 5: MEDICC2 copy number sample trees. Supplementary figure 6: MEDICC tree of all exome samples demonstrating that samples cluster together by patient, and not by melanoma subtype. Supplementary figure 7: SCNA frequency of cutaneous (a), acral (b) and melanoma of unknown primary (MUP, c). Supplementary figure 8: Correlation between liver copy number distance to other sites and time of emergence after stage IV diagnosis. Supplementary figure 9: Examination of tumour heterogeneity of alterations to antigen-presentation machinery genes,with site and patient annotation. Supplementary figure 10: Boxplots indicating the proportion of losses in the cohort for each segment. Supplementary figure 11: Balance of expression between nonsynonymous mutations that were not predicted to be neoantigens and clonal predicted neoantigens. Supplementary figure 12: Barplot of TIL infiltration score frequencies, determined by pathologist assessment of histology, across all samples. Supplementary figure 13: Number of samples per patient that are classified as either none-low in terms of TILs or moderate-heavy. Supplementary figure 14: Histogram of purity for samples with RNA-seq data. Supplementary figure 15: TME deconvolution. Supplementary figure 16: The effect of metastatic site on transcriptional profiles. Supplementary figure 17: Association of PHF3 copy number with expression. Supplementary figure 18: Overview of gene fusions identified in RNA-seq data. Supplementary figure 19: Comparison of ploidy estimates from panel sequencing data, exome data and FISH. Supplementary figure 20: Comparison of ploidy estimates in panel, exome, FISH and single cell data. Supplementary figure 21: FACs sort plot for CRUKP2567 diaphragmatic metastasis. Supplementary figure 22: Ploidy and wGII values from single cell sequencing of FACS-sorted tumour cells. Supplementary figure 23: Copy number profiles on chromosome 5 for bulk samples from primary and DI_2_R2, a diaphragmatic metastasis. Supplementary figure 24: Histogram of purity of samples for which RNA-seq was performed faceted by patient. Supplementary figure 25: Histogram of purity of samples for which RNA-seq was performed faceted by tissue site.
