# Supplementary material for: Late-Stage Metastatic Melanoma Emerges through a Diversity of Evolutionary Pathways
Source: Cancer Discov. 2023 Mar 28;13(6):1364–85. doi: 10.1158/2159-8290.CD-22-1427 (PMC10236155; doi:10.1158/2159-8290.CD-22-1427)
Supplement: Supplementary Figures S1-S25 — Supplementary figure 1: Cohort overview. Number of samples sequenced with whole exome, panel or whole RNA sequencing. Supplementary figure 2: Phylogeny and WGD events in CRUKP1047. Supplementary figure 3: Ploidy and SCNA burden. Supplementary figure 4: Overview of each case. Supplementary figure 5: MEDICC2 copy number sample trees. Supplementary figure 6: MEDICC tree of all exome samples demonstrating that samples cluster together by patient, and not by melanoma subtype. Supplementary figure 7: SCNA frequency of cutaneous (a), acral (b) and melanoma of unknown primary (MUP, c). Supplementary figure 8: Correlation between liver copy number distance to other sites and time of emergence after stage IV diagnosis. Supplementary figure 9: Examination of tumour heterogeneity of alterations to antigen-presentation machinery genes,with site and patient annotation. Supplementary figure 10: Boxplots indicating the proportion of losses in the cohort for each segment. Supplementary figure 11: Balance of expression between nonsynonymous mutations that were not predicted to be neoantigens and clonal predicted neoantigens. Supplementary figure 12: Barplot of TIL infiltration score frequencies, determined by pathologist assessment of histology, across all samples. Supplementary figure 13: Number of samples per patient that are classified as either none-low in terms of TILs or moderate-heavy. Supplementary figure 14: Histogram of purity for samples with RNA-seq data. Supplementary figure 15: TME deconvolution. Supplementary figure 16: The effect of metastatic site on transcriptional profiles. Supplementary figure 17: Association of PHF3 copy number with expression. Supplementary figure 18: Overview of gene fusions identified in RNA-seq data. Supplementary figure 19: Comparison of ploidy estimates from panel sequencing data, exome data and FISH. Supplementary figure 20: Comparison of ploidy estimates in panel, exome, FISH and single cell data. Supplementary figure 21: FACs sort plot f [file cd-22-1427_supplementary_figures_s1-s25_suppsf1.pdf]

Supplementary figures - Late-stage metastatic melanoma emerges through a diversity of evolutionary pathways

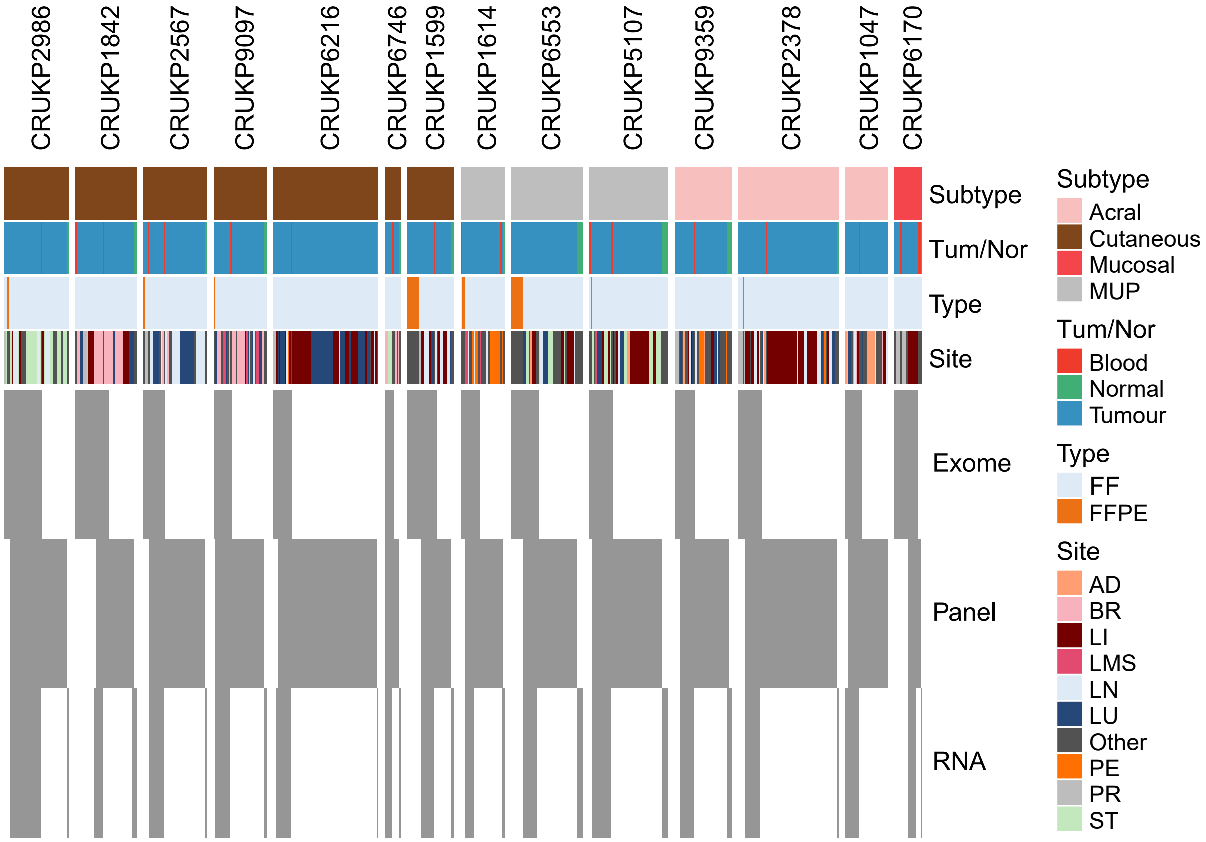

Supplementary figure 1: Cohort overview. Number of samples sequenced with whole exome, panel or whole RNA sequencing. Also shown are patient subtype, type of sample and metastatic site. Vertical grey lines represent included sam-ples. AD: Adrenal, BR: Brain, BO: Bone, DI: Diaphragm, LI: Liver, LN: LymphNode, LMS: Leptomeningeal, LU: Lung, ME: Mesenteric, MU: Muscle, ON: OpticNerve, PA: Pancreas, PC: Pericardium, PE: Peritoneal, PI: Pituitary, PLF: PleuralFluid, PR: Primary, PV: Paravertebral, ST: SoftTissue, SP: Spleen. An “a” indicates archival FFPE samples for metastatic samples.

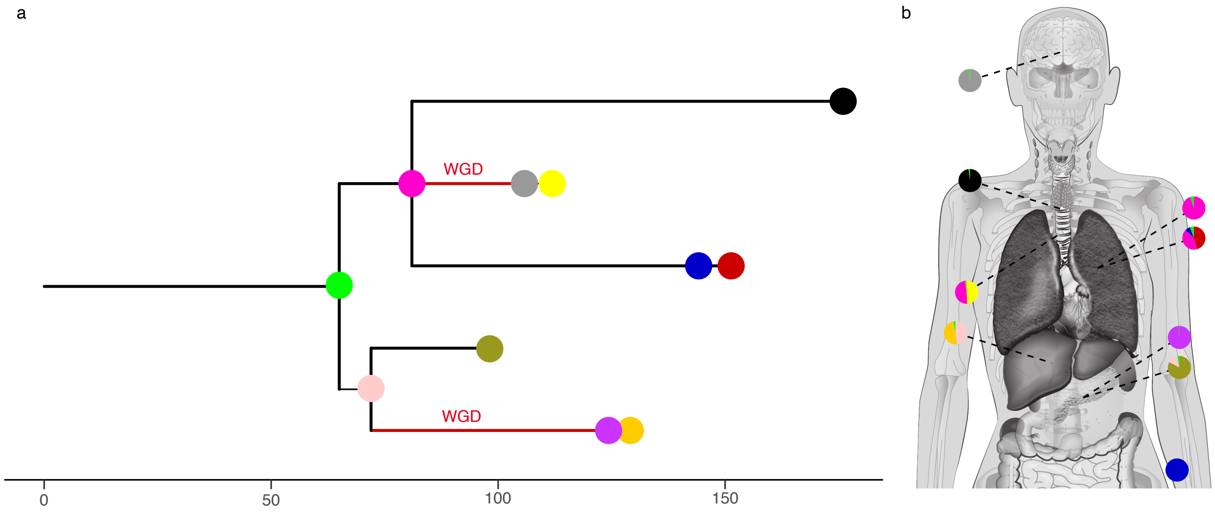

Supplementary figure 2: Phylogeny and WGD events in CRUKP1047. (a) Phylogeny of patient CRUKP1047 with WGD events annotated in red. (b) Diagram showing the anatomical distribution of clones.

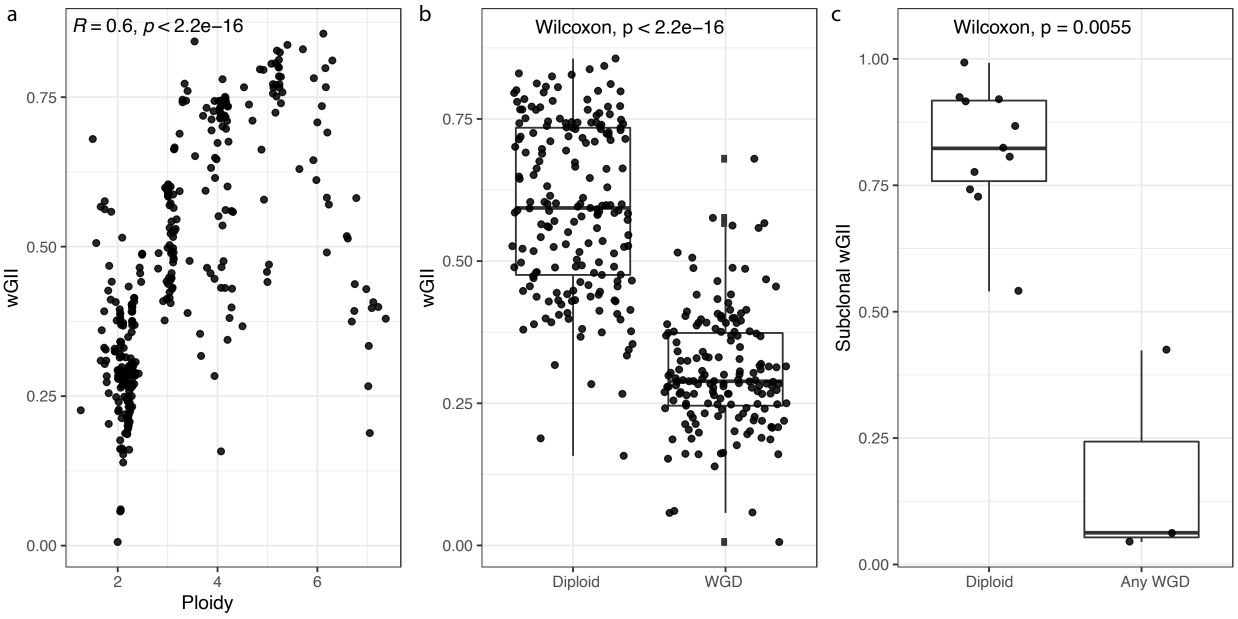

Supplementary figure 3: Ploidy and SCNA burden. (a) Weighted genome instability index (wGII, weighted proportion of the genome with SCNAs) by ploidy for each panel-sequenced sample. (b) wGII by WGD status for each panel-sequenced sample. (c) Subclonal wGII (intra-patient SCNA heterogeneity) by WGD status for each patient.

CRUKP2986 Cutaneous (Anorectal)

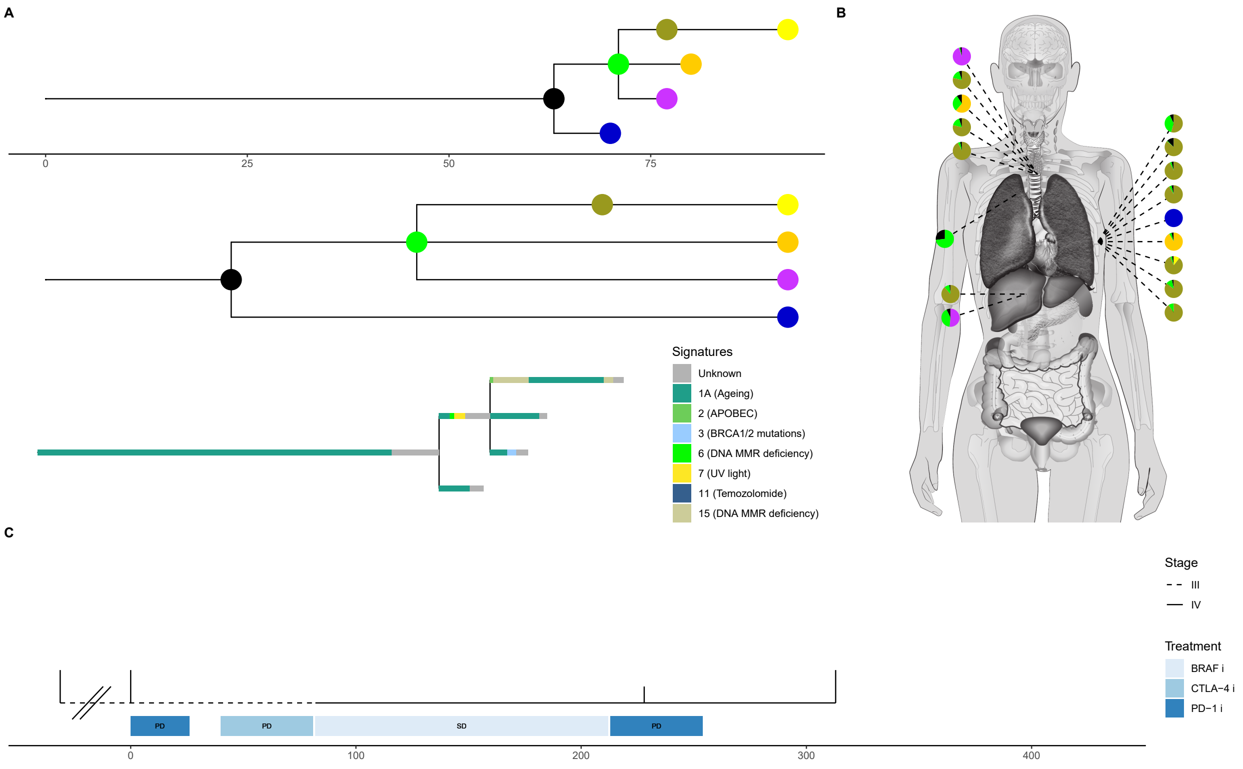

CRUKP1842 Cutaneous

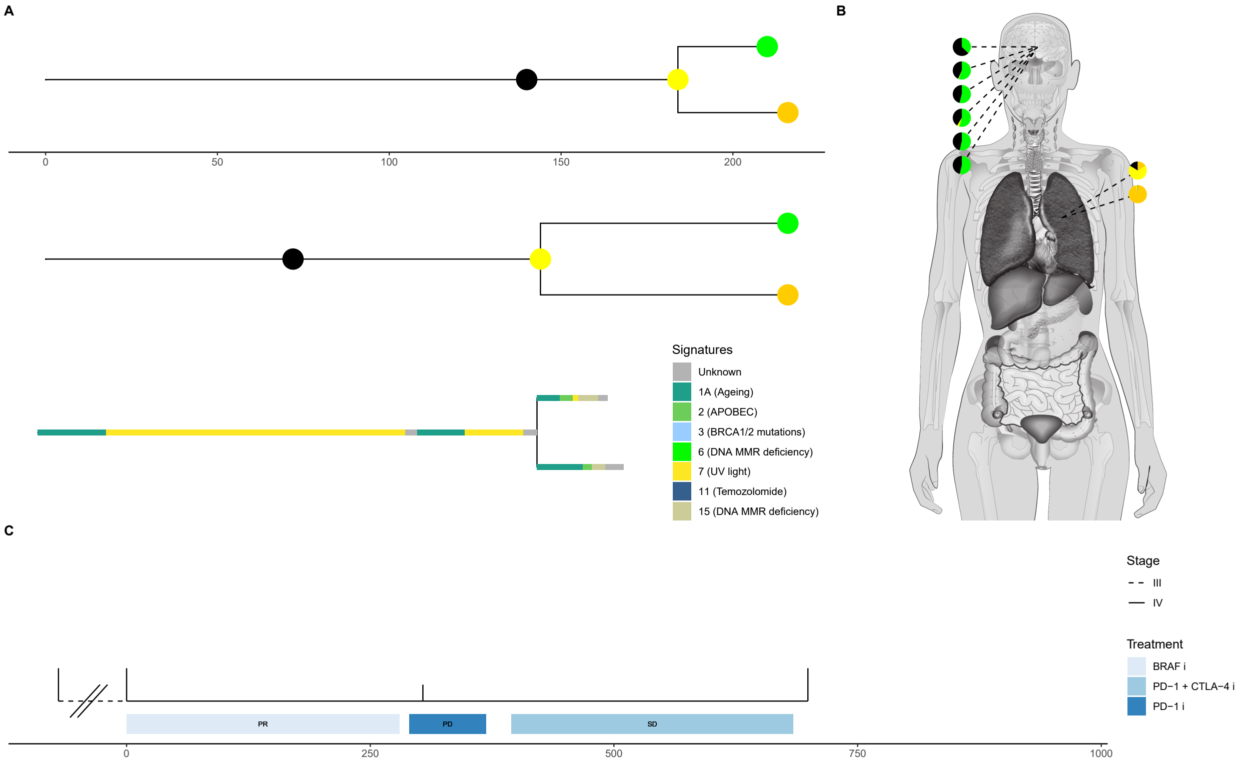

CRUKP2567 Cutaneous

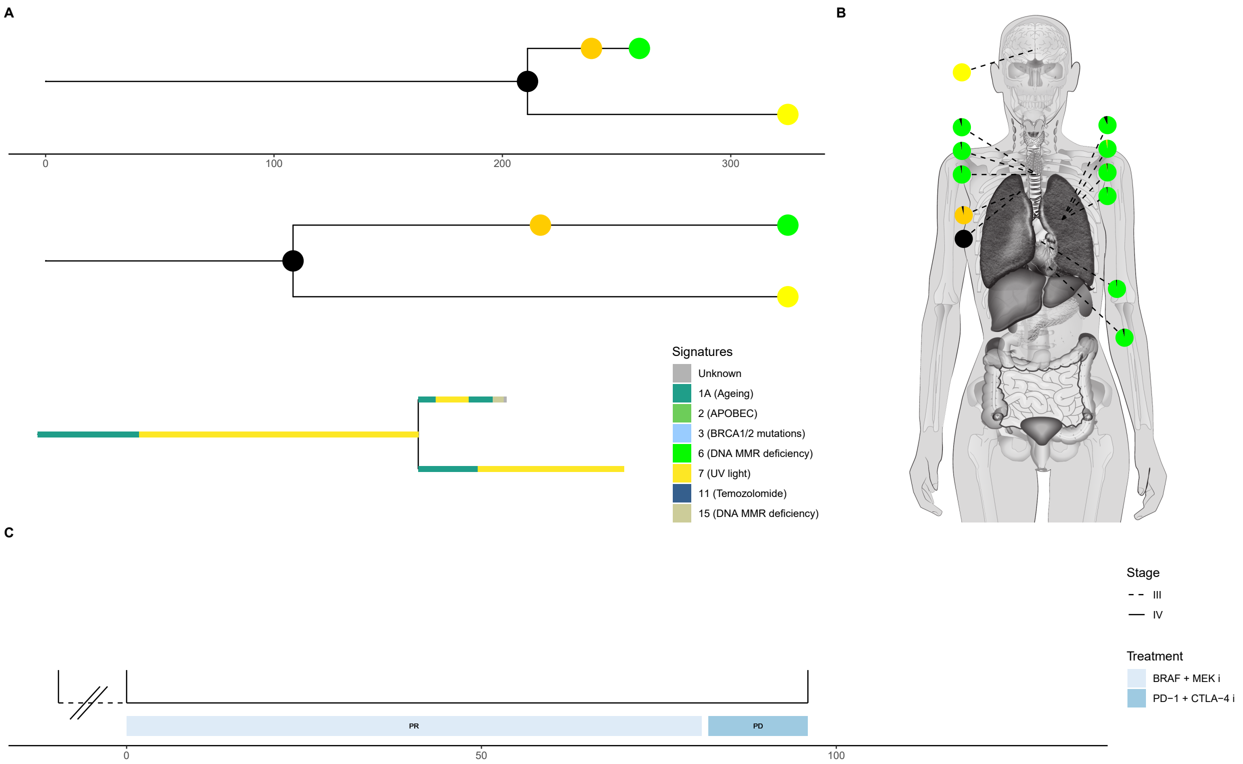

CRUKP9097 Cutaneous

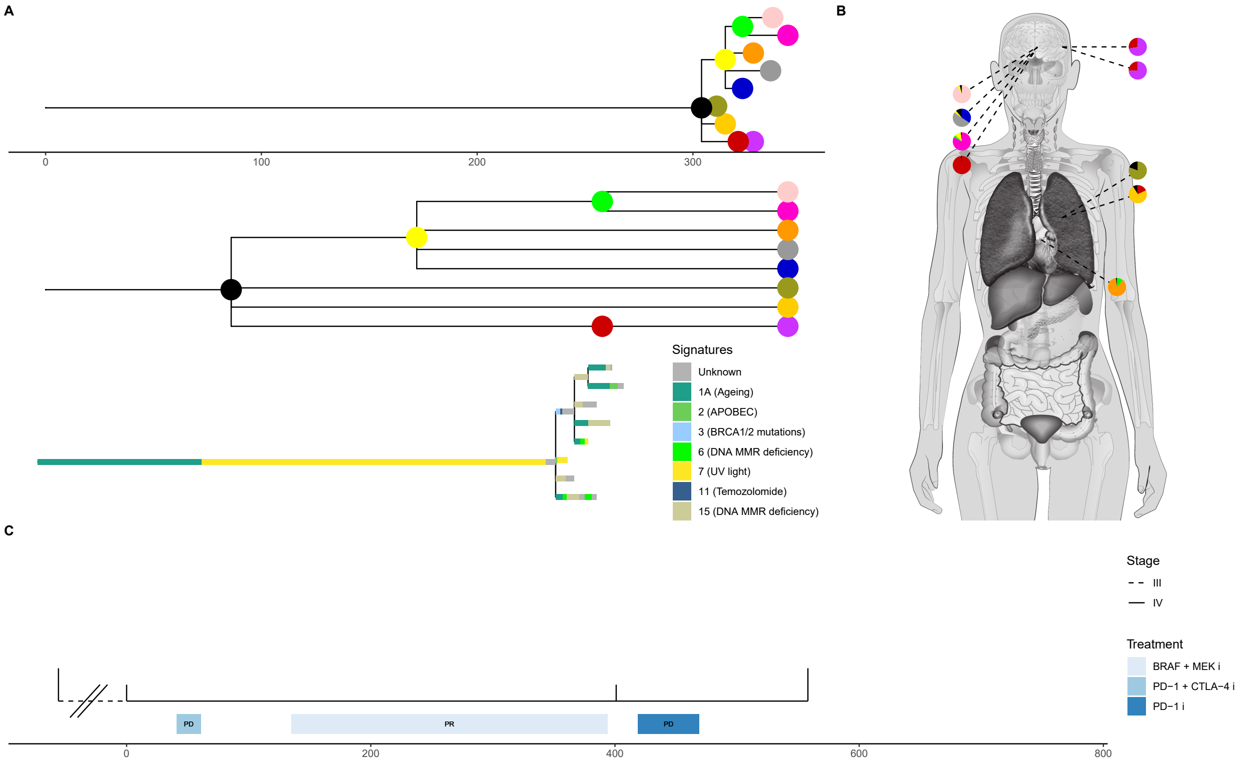

CRUKP6216 Cutaneous

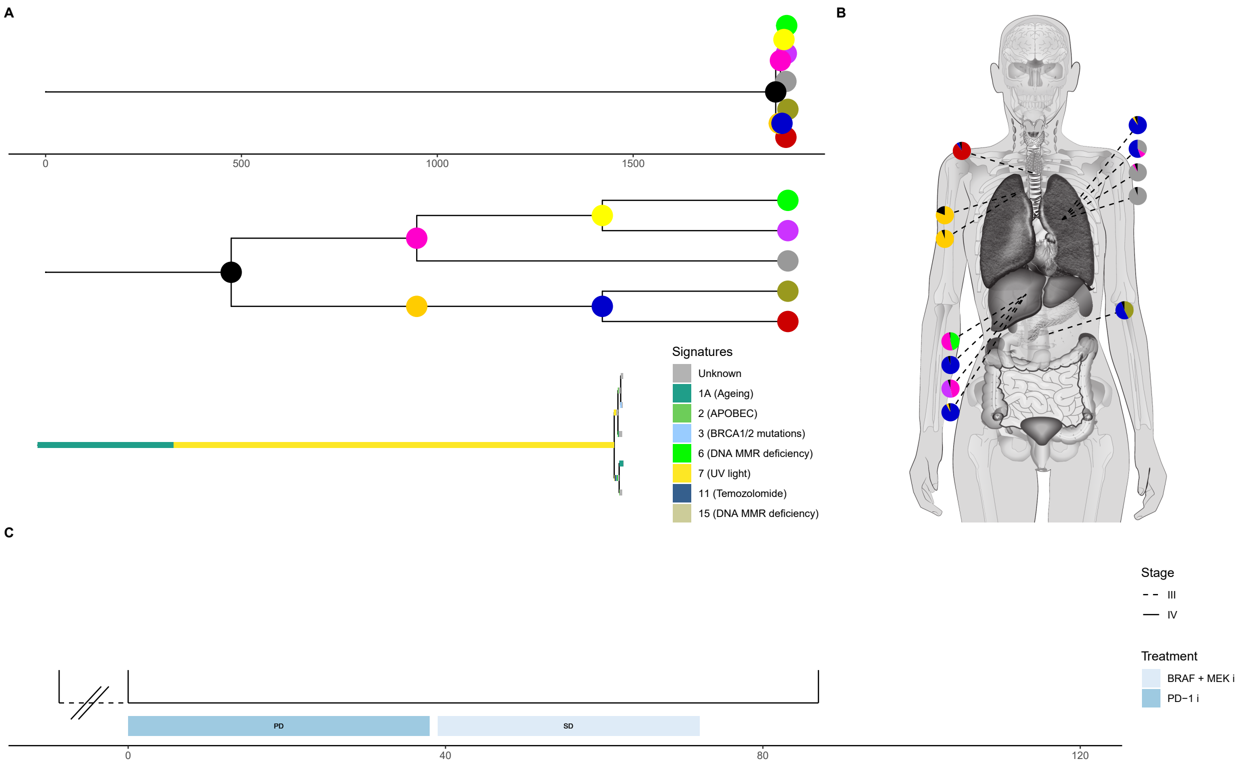

CRUKP6746 Cutaneous

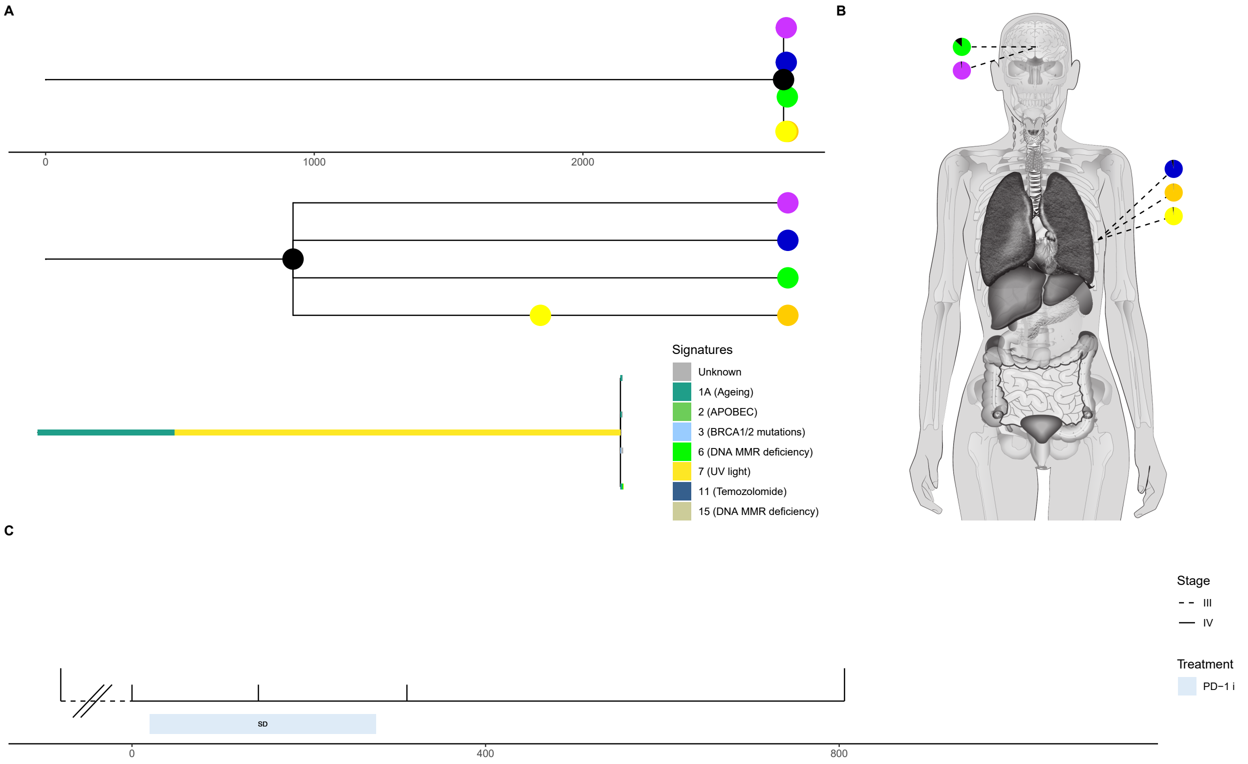

CRUKP1599 Cutaneous CHEMO

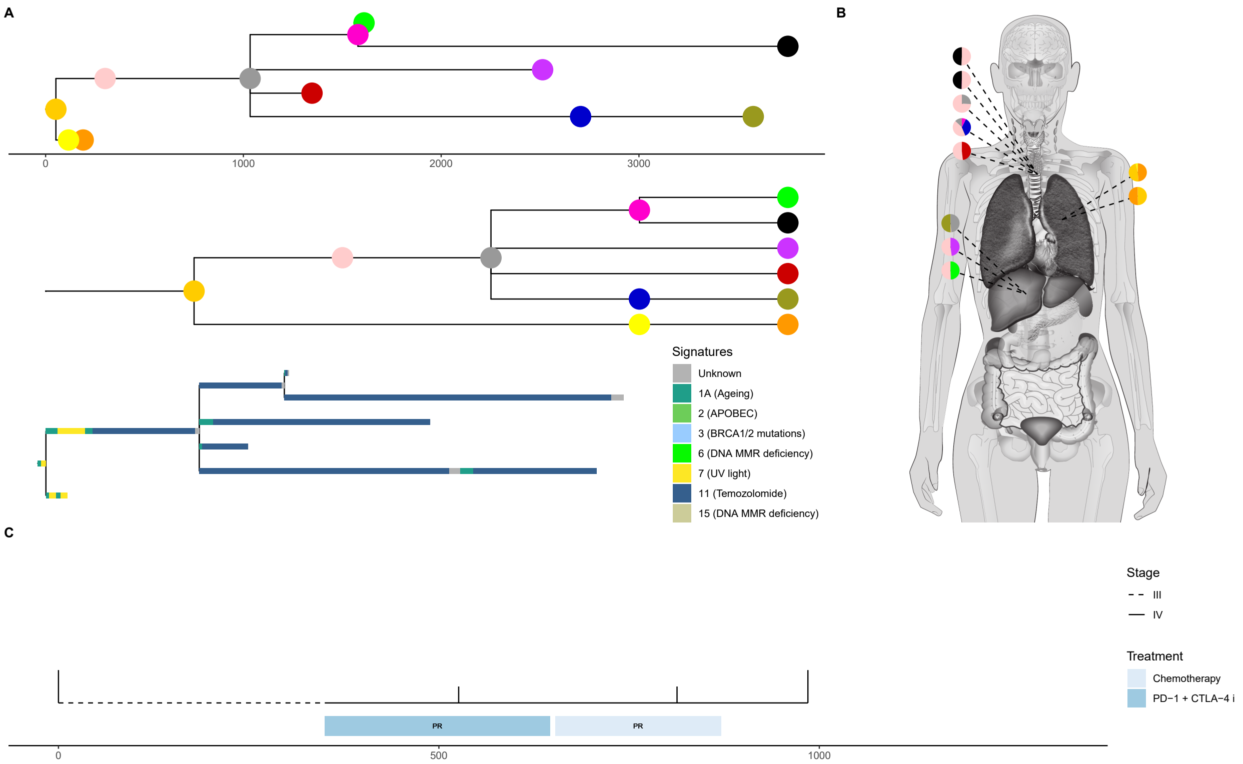

CRUKP1614 MUP

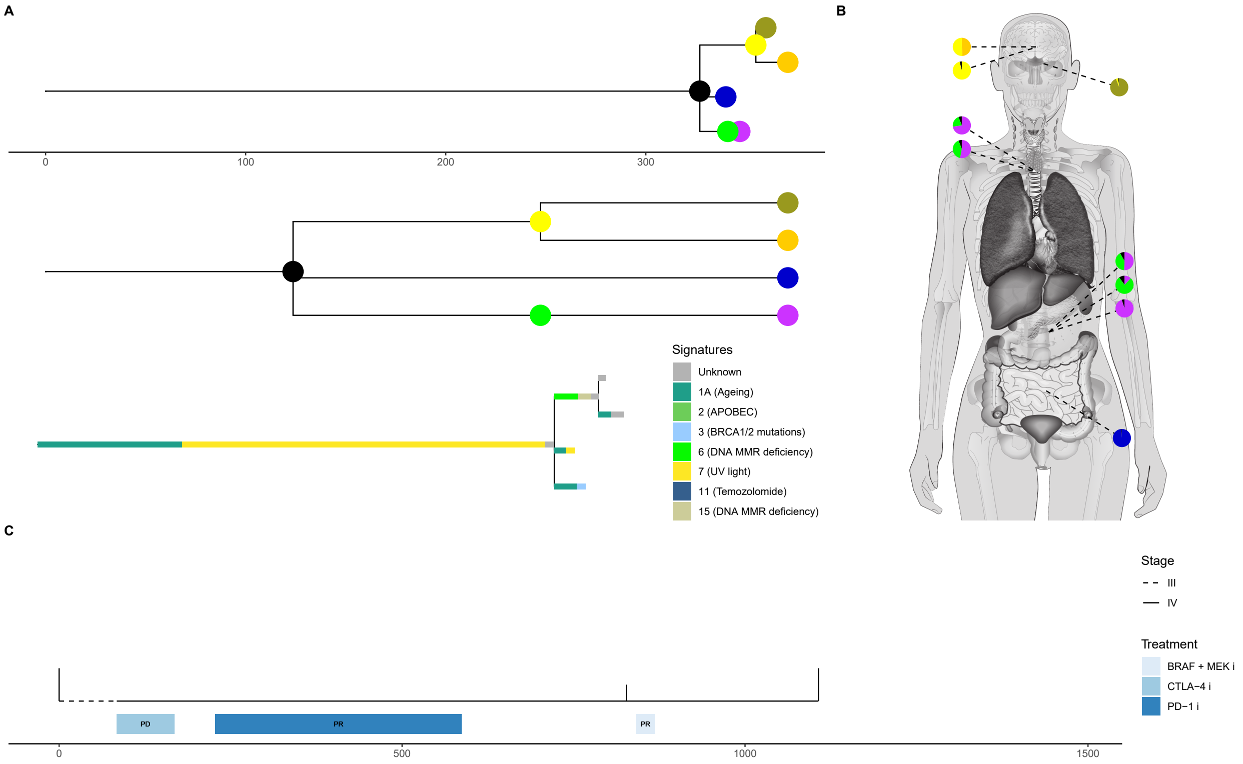

CRUKP6553 MUP

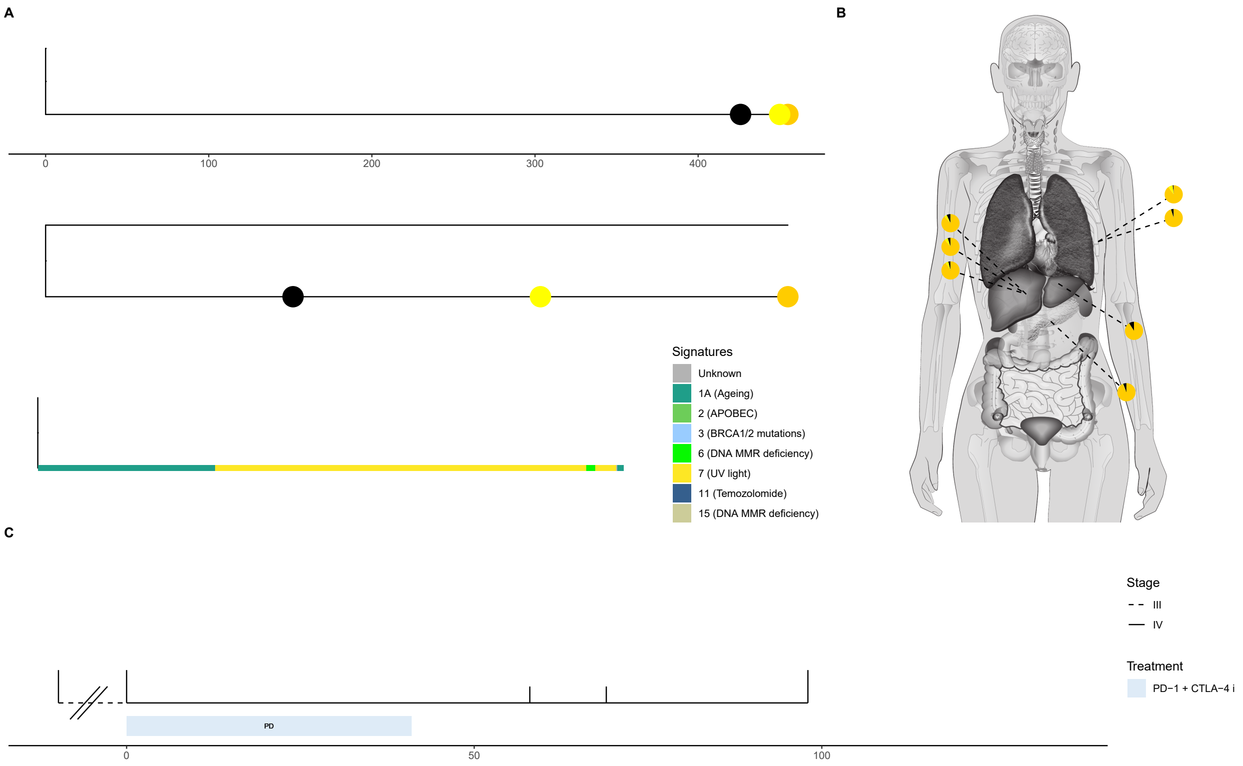

CRUKP5107 MUP CHEMO

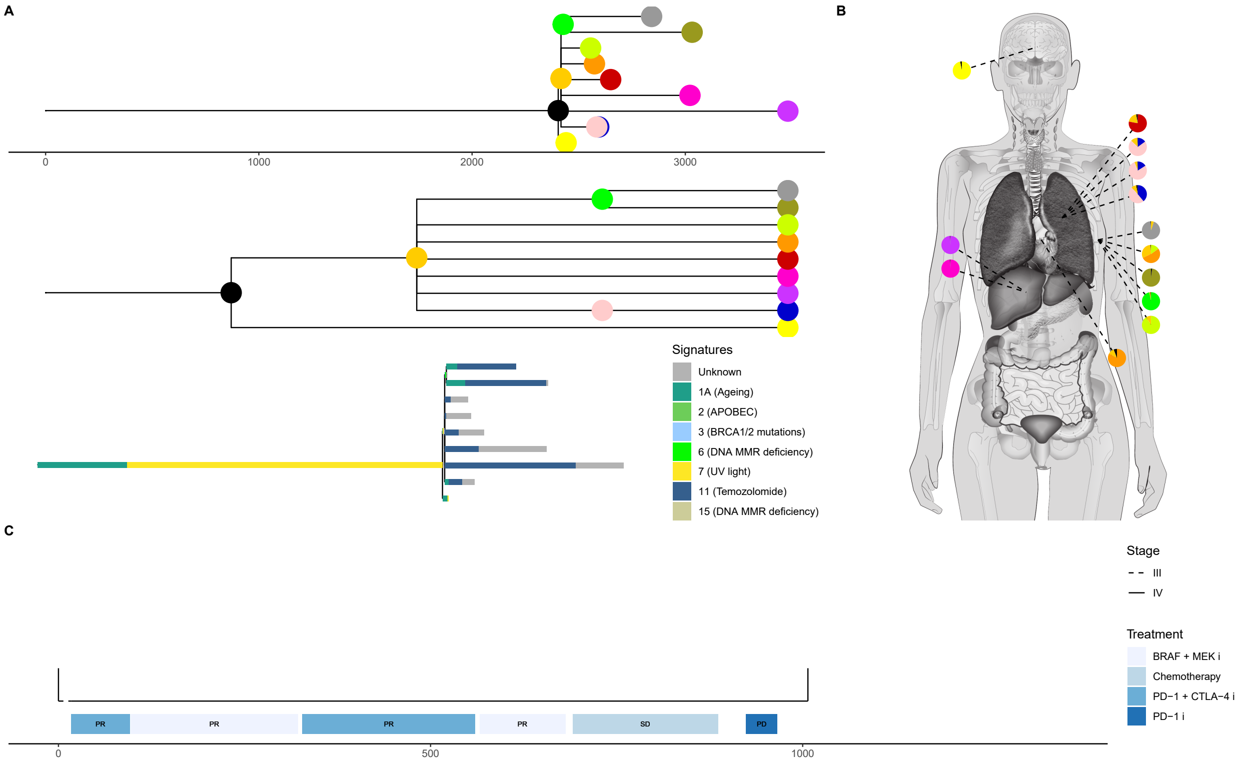

CRUKP9359 Acral

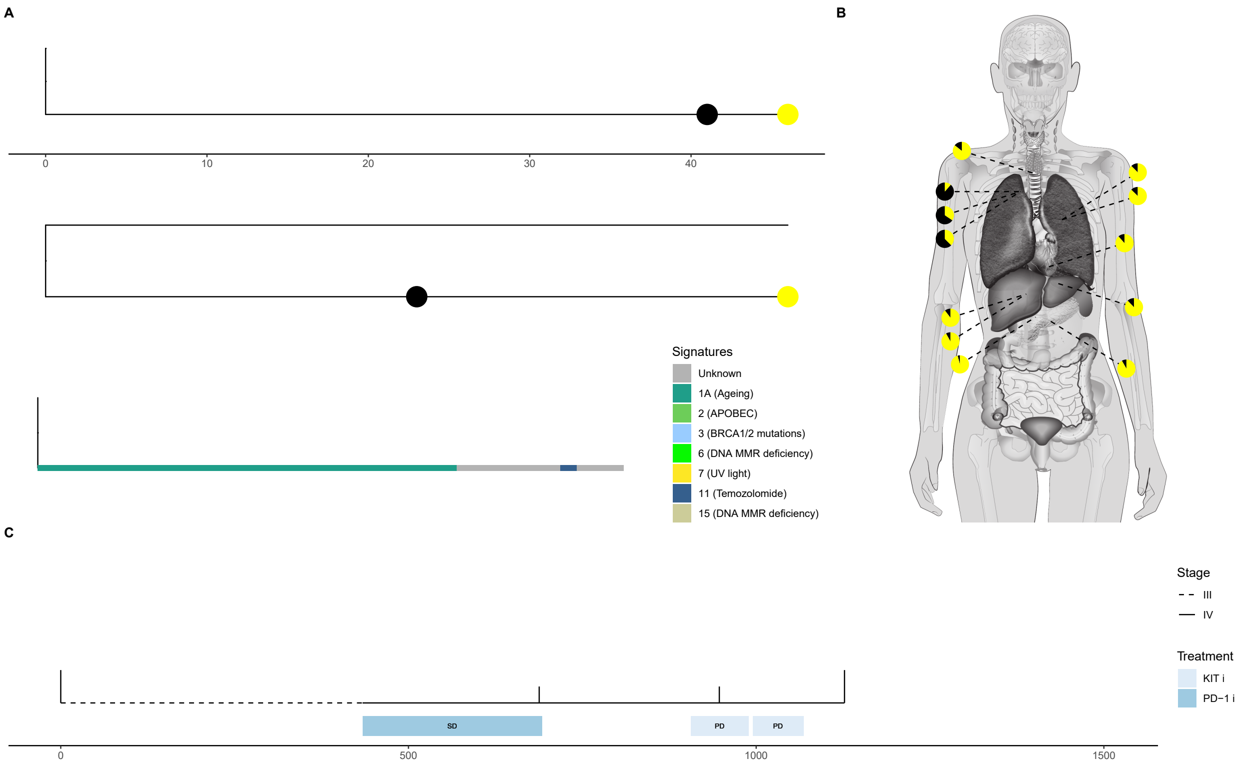

CRUKP2378 Acral

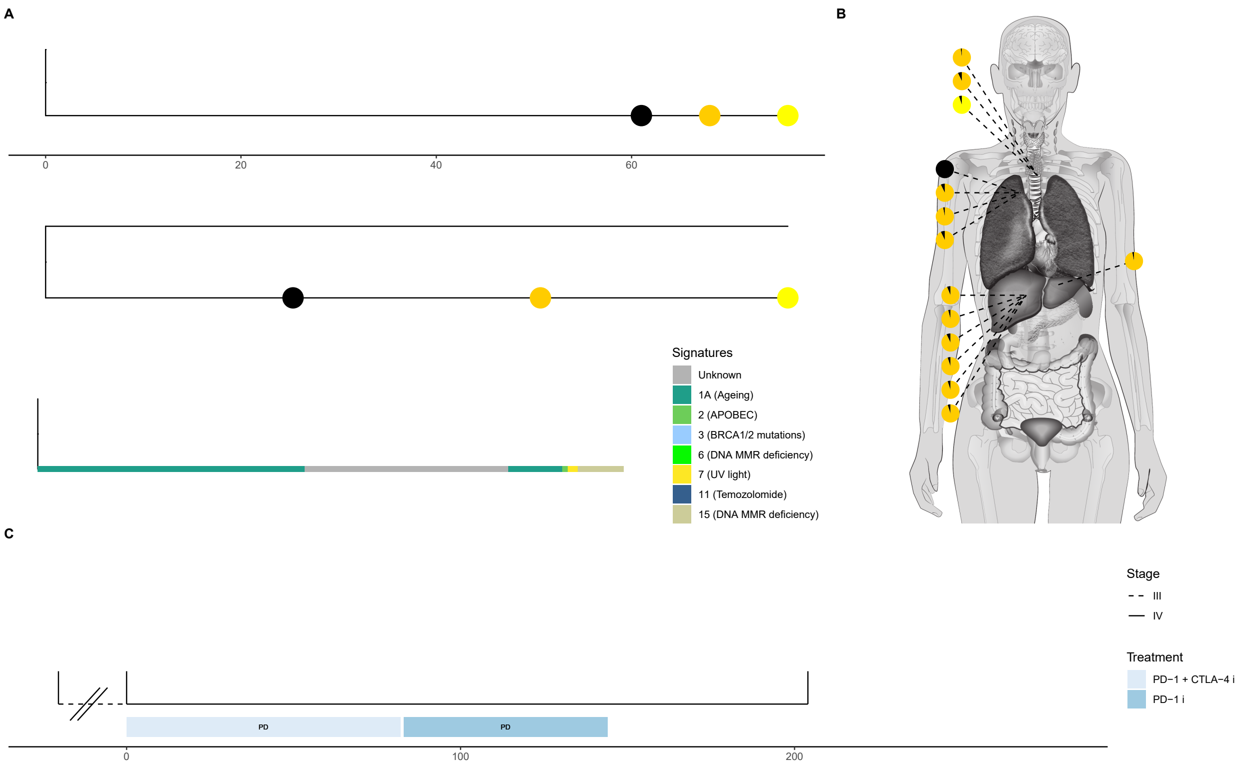

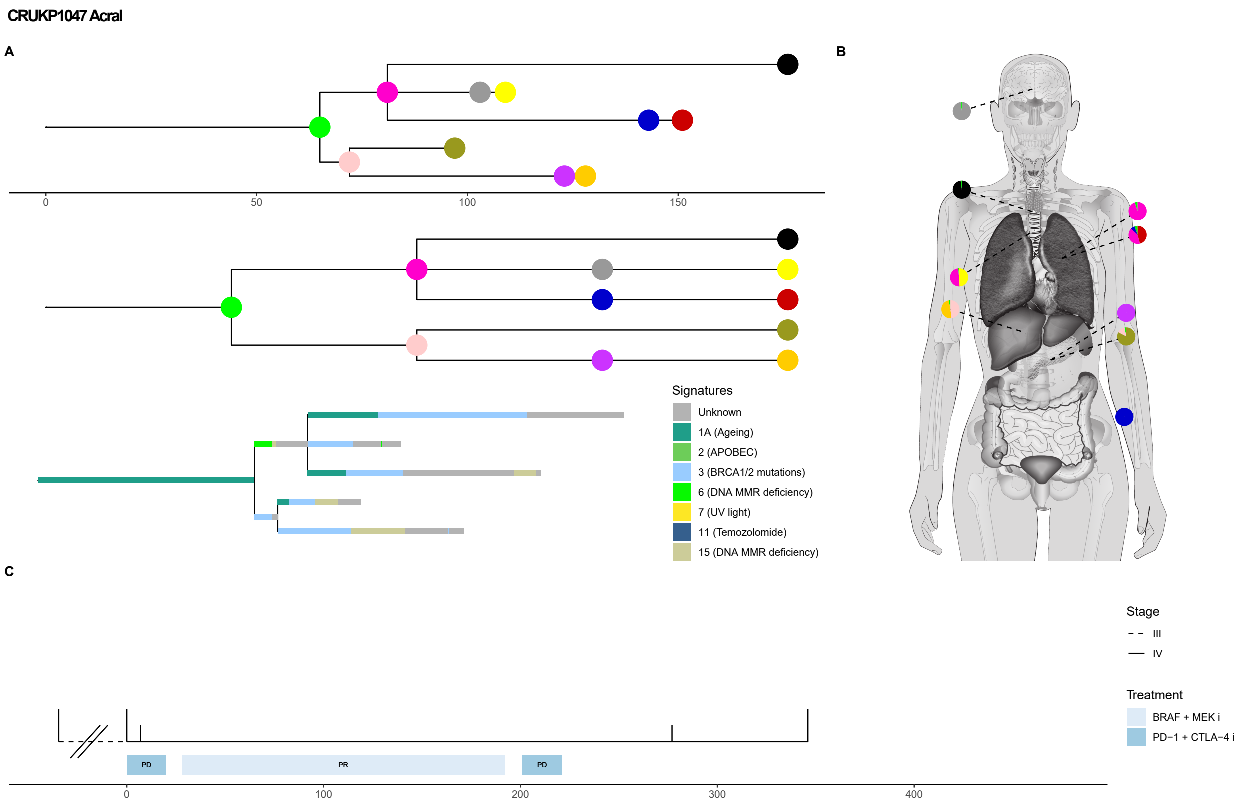
CRUKP1047 Acral
A
0
50
100
150
B
Signatures
Unknown
1A (Ageing)
2 (APOBEC)
3 (BRCA1/2 mutations)
6 (DNA MMR deficiency)
7 (UV light)
11 (Temozolomide)
15 (DNA MMR deficiency)
C
Stage
III
IV
Treatment
BRAF + MEK i
PD−1 + CTLA−4 i
PD
PR
PD
0
100
200
300
400

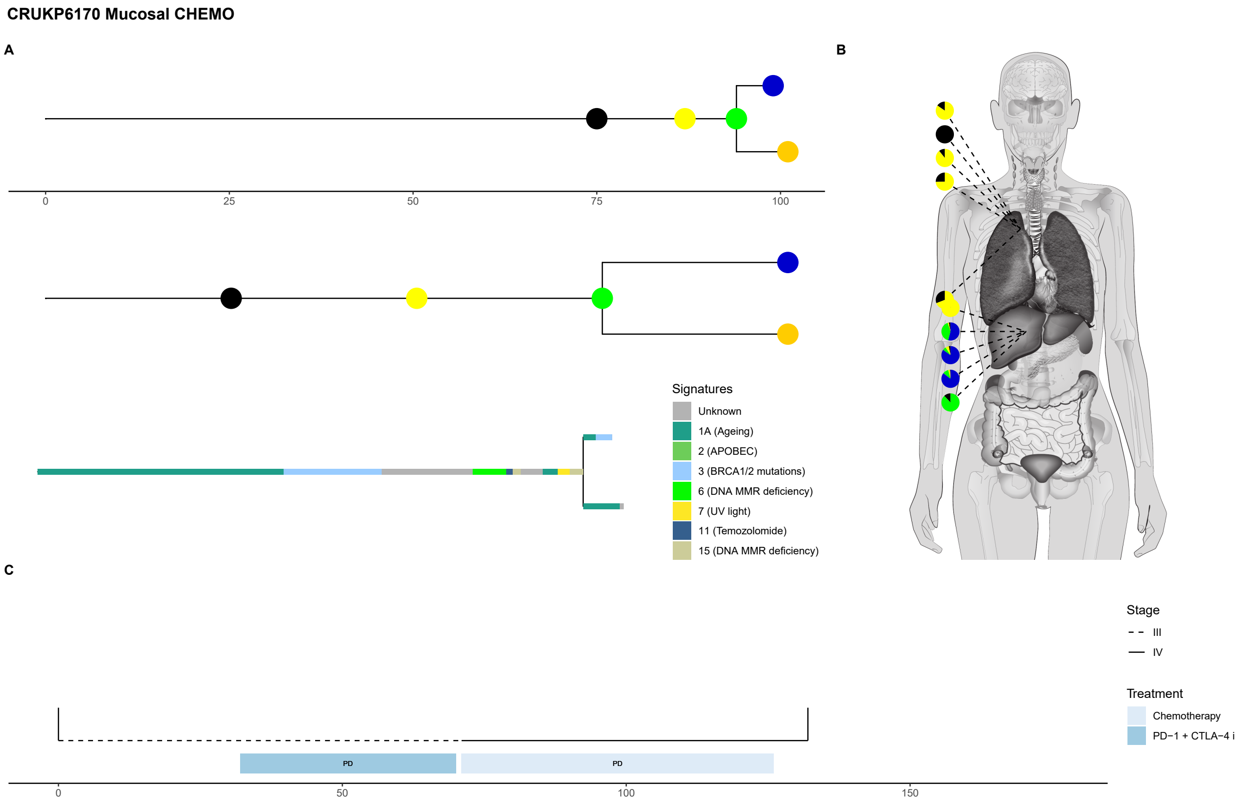

Supplementary figure 4: Overview of each case. Case IDs are indicated at the top and each panel is subdivided into (A) phylogeny, cladogram, and mutational signature trees, (B) sampling regime and (C) clinical course. Coloured circles indicate mutation clusters in the trees and pie charts indicate the CCFs (proportions) of each cluster in a specific sample. The clinical course shows the staging, treatment, and responses (complete response (CR), partial response (PR), progressive disease (PD), and stable disease (SD)) along time in days.

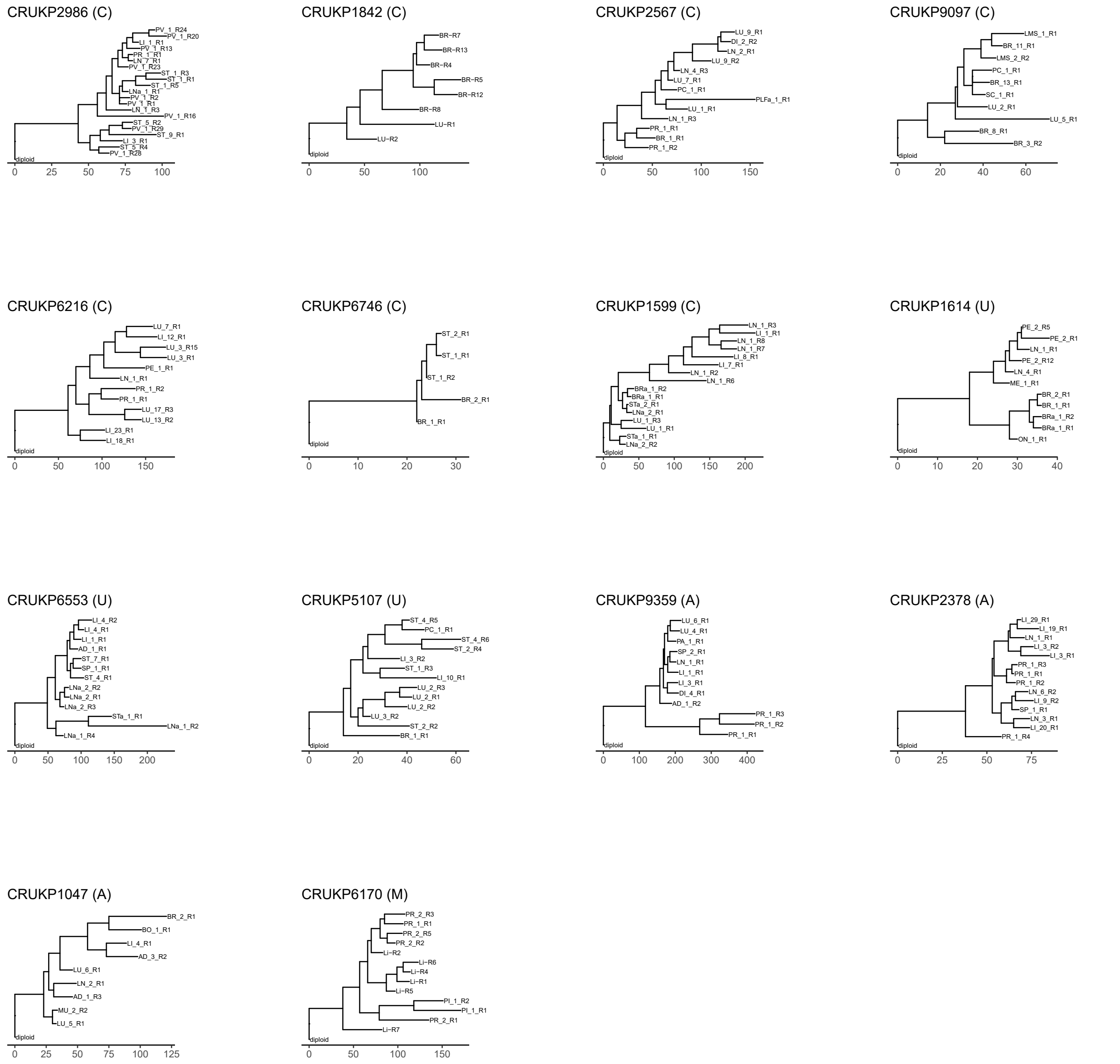

Supplementary figure 5: MEDICC2 copy number sample trees. AD: Adrenal, BR: Brain, BO: Bone, DI: Diaphragm, LI: Liver, LN: LymphNode, LMS: Leptomeningeal, LU: Lung, ME: Mesenteric, MU: Muscle, ON: OpticNerve, PA: Pancreas, PC: Pericardium, PE: Peritoneal, PI: Pituitary, PLF: PleuralFluid, PR: Primary, PV: Paravertebral, ST: SoftTissue, SP: Spleen. An "a" indicates archival FFPE samples for metastatic samples.

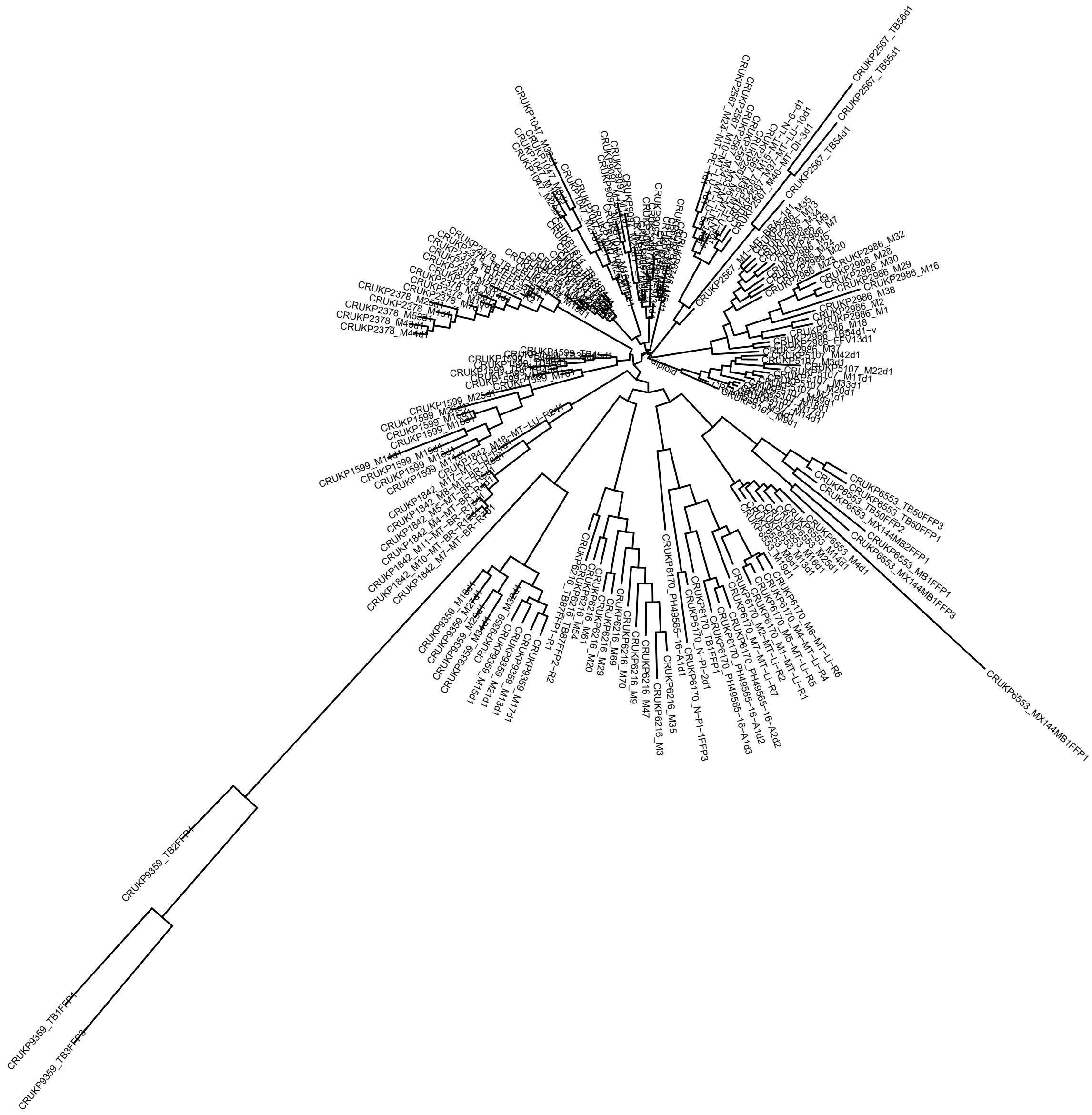

Supplementary figure 6: MEDICC tree of all exome samples demonstrating that samples cluster together by patient, and not by melanoma subtype.

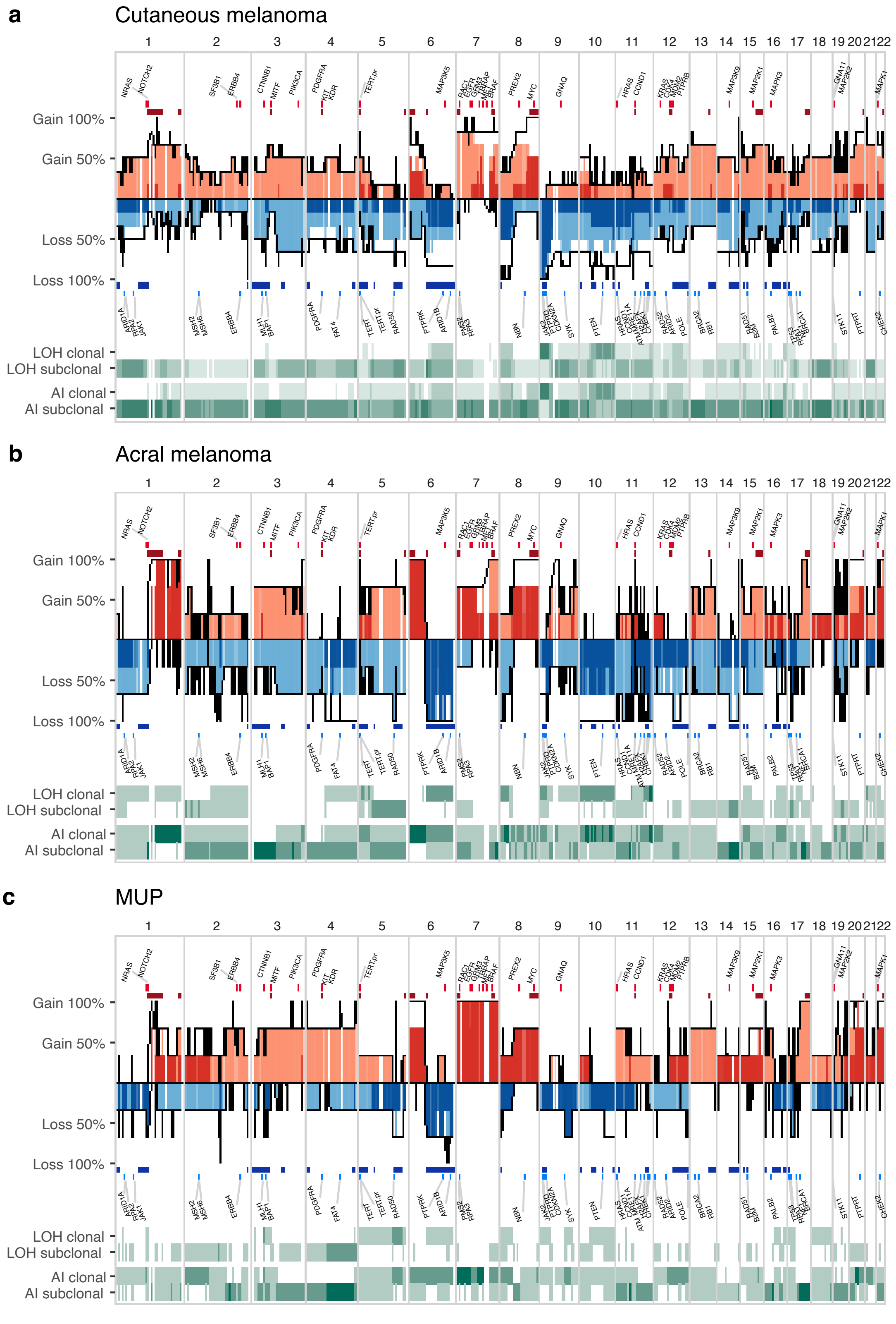

Supplementary figure 7: SCNA frequency of cutaneous (a), acral (b) and melanoma of unknown primary (MUP, c). Red and dark blue indicate clonal events, orange and light blue indicate subclonal events. Also shown are the clonal and subclonal frequencies of loss of heterozygosity (LOH) and allelic imbalance (AI). Annotated along the genome are melanoma driver genes and copy number events enriched in The Cancer Genome Atlas (TCGA) skin cutaneous melanoma cohort. Only WES data is shown.

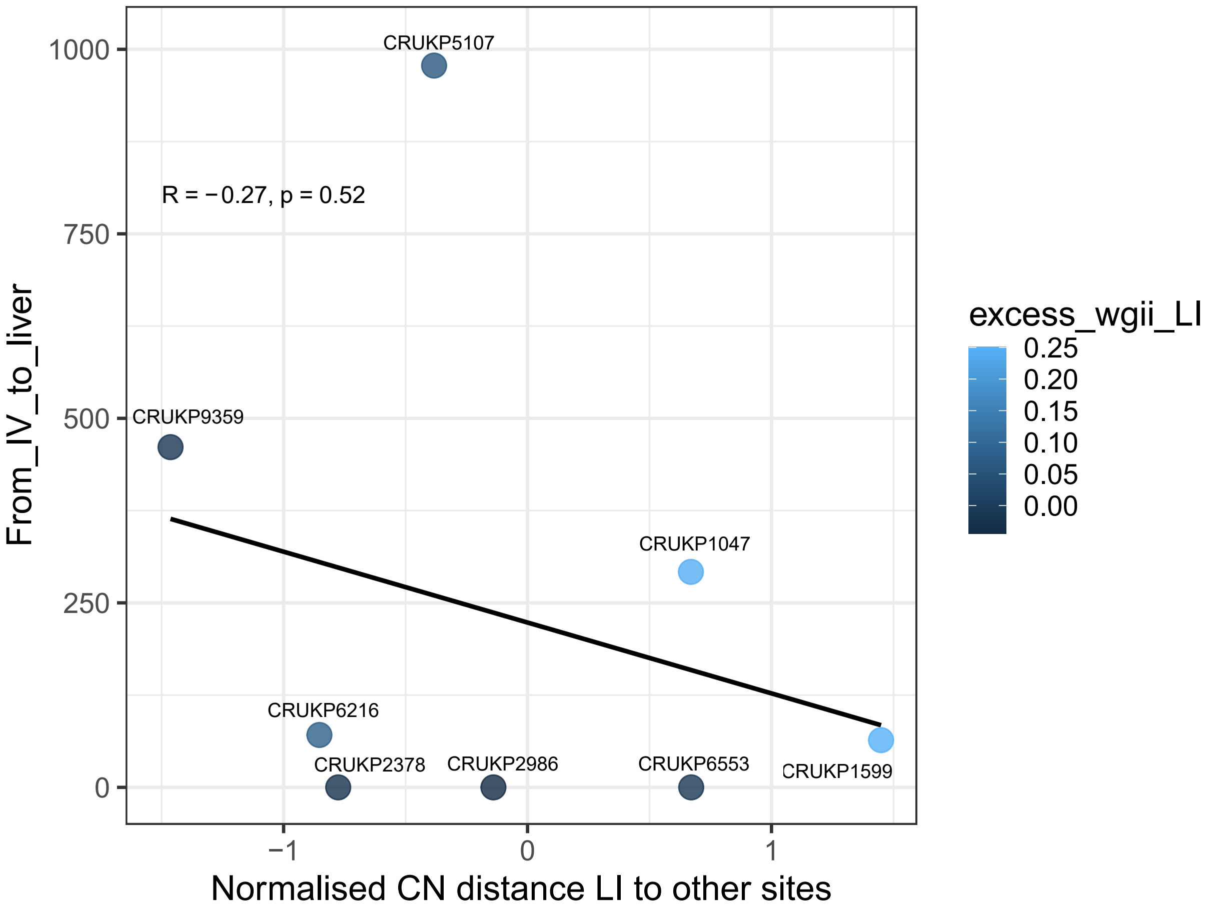

Supplementary figure 8: Correlation between liver copy number distance to other sites and time of emergence after stage IV diagnosis. Colour indicates the difference between the mean wGII in the liver and the mean of other sites. Opposite to brain tumours, liver tumours with larger SCNA distances seem to emerge earlier in the clinical course.

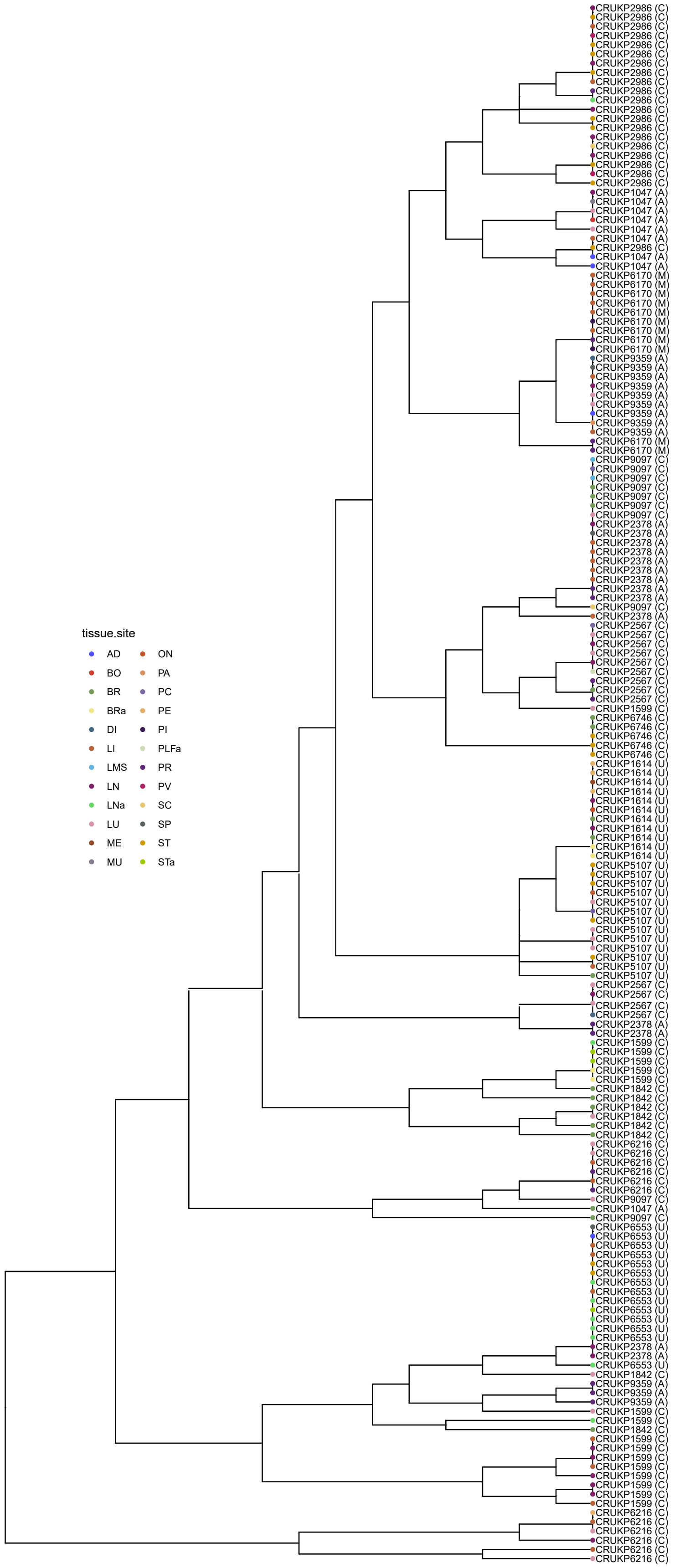

Supplementary figure 9: Examination of tumour heterogeneity of alterations to antigen-presentation machinery genes,with site and patient annotation. AD: Adrenal, BR:15 Brain, BO: Bone, DI: Diaphragm, LI: Liver, LN: LymphNode, LMS: Leptomeningeal, LU: Lung, ME: Mesenteric, MU: Muscle, ON: OpticNerve, PA: Pancreas, PC: Pericardium, PE: Peritoneal, PI: Pituitary, PLF: Pleural fluid, PR: Primary, PV: Paravertebral, ST: SoftTissue, SP: Spleen. An "a" indicates archival FFPE samples for metastatic samples.

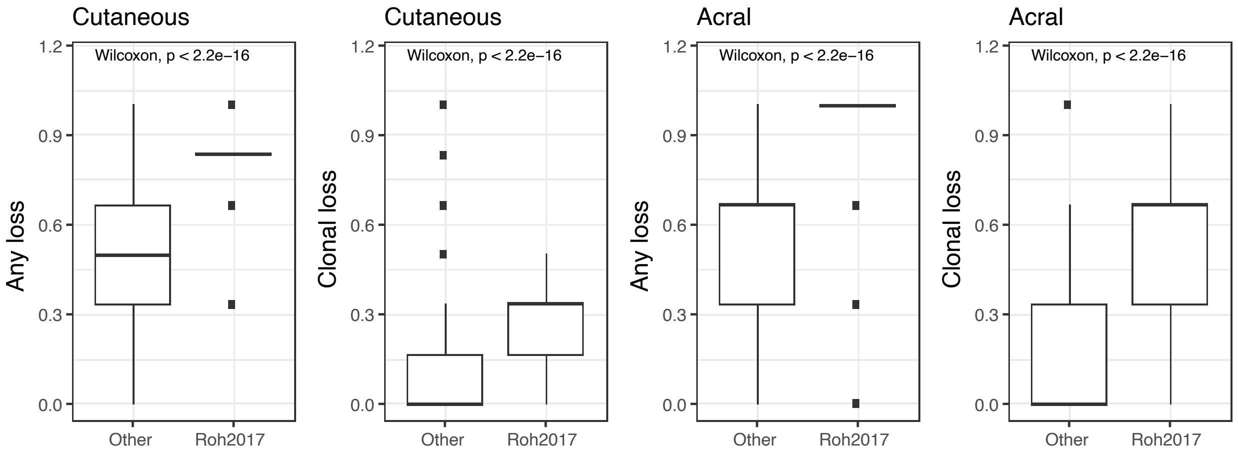

Supplementary figure 10: Boxplots indicating the proportion of losses in the cohort for each segment. Segments overlapping regions identified as lost more frequently in non-responders by Roh et al., 2017 were also lost more frequently in our cohort, both when all segments were included (left), as well as when only clonal losses were included (right).

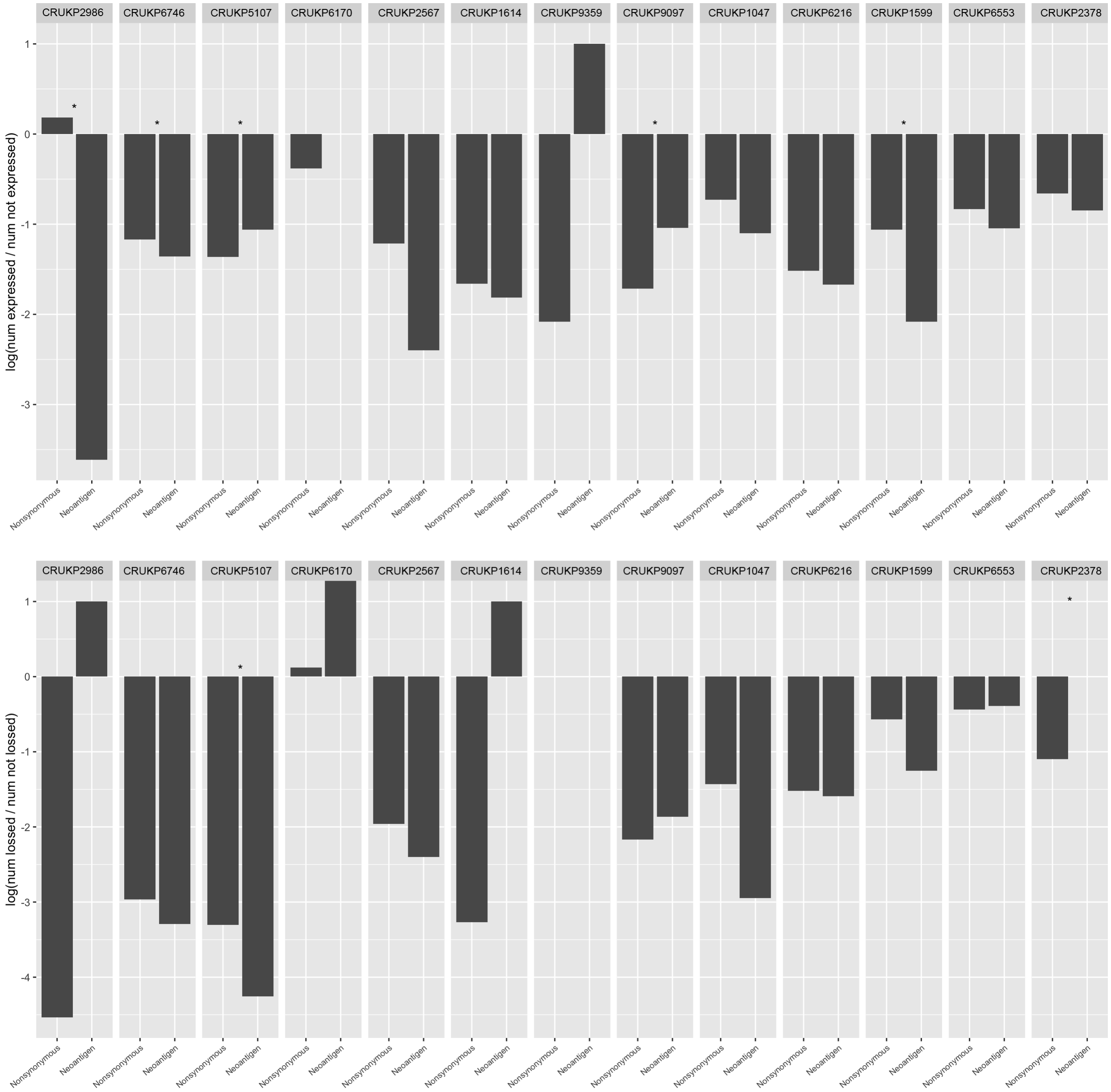

Supplementary figure 11: Top: Balance of expression between nonsynonymous mutations that were not predicted to be neoantigens and clonal predicted neoantigens. Values below 0 indicate a downregulation in expression of neoantigens (i.e., more were not expressed than were expressed), values above 0 the contrary. Bottom: Balance of copy number loss (lost vs not lost).

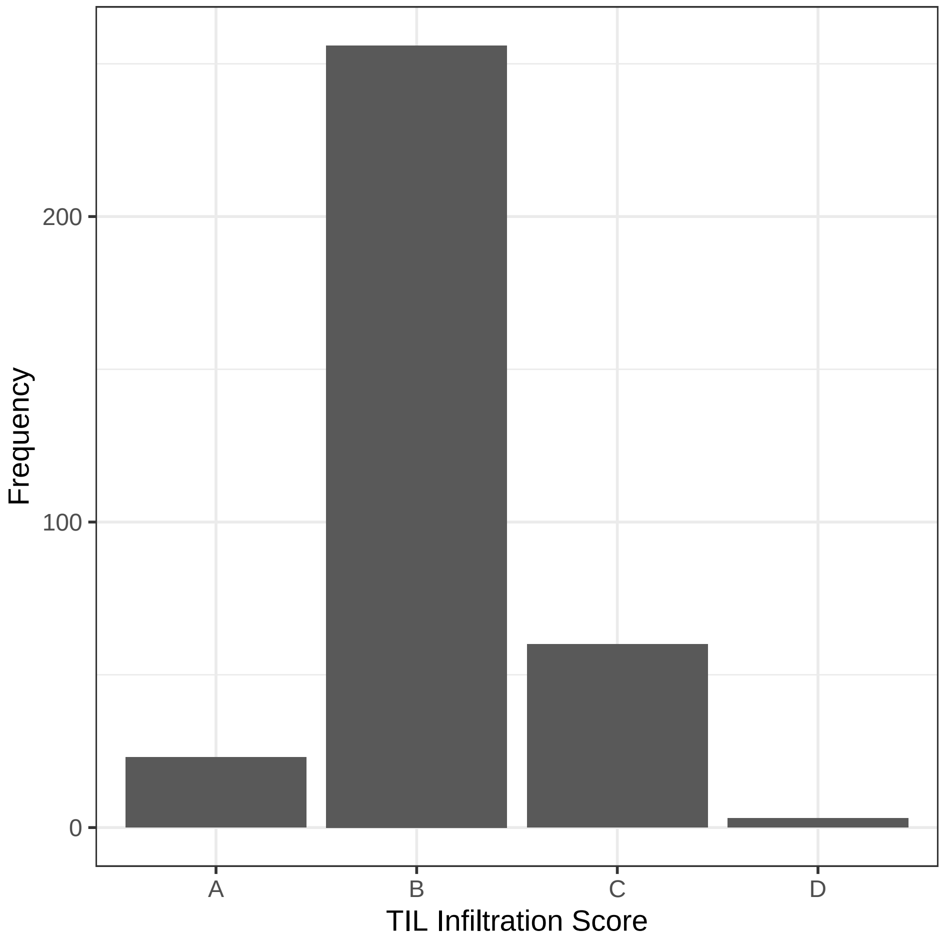

Supplementary figure 12: Barplot of TIL infiltration score frequencies, determined by pathologist assessment of histology, across all samples. A = Nil, B = Minimal, C = Moderate, D = Heavy.

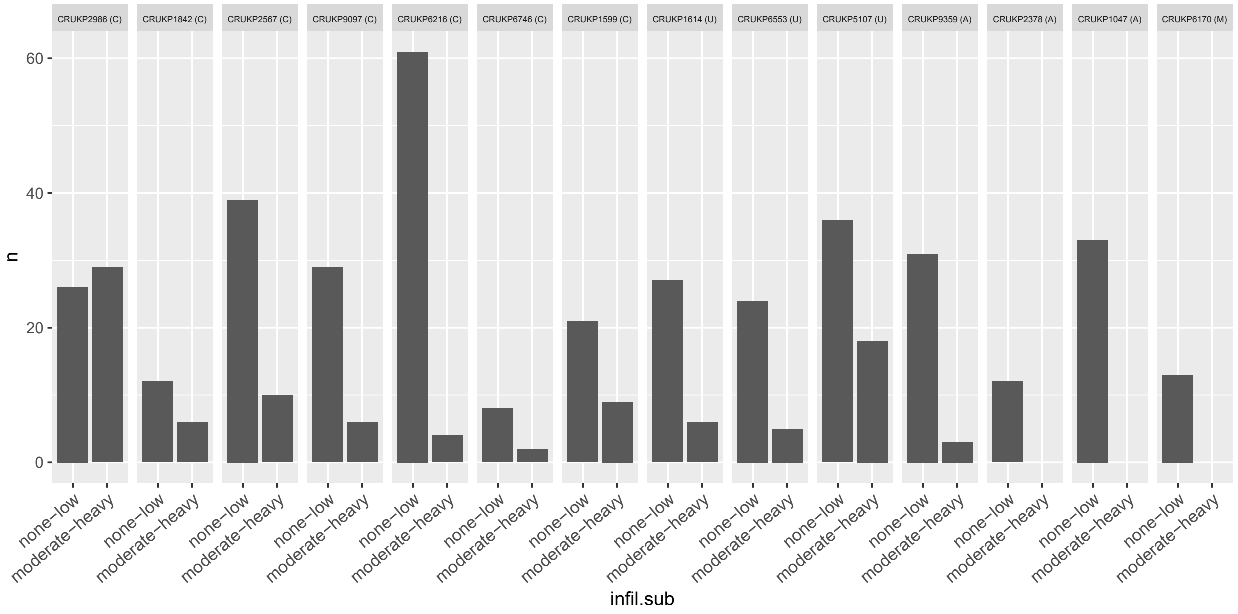

Supplementary figure 13: Number of samples per patient that are classified as either none-low in terms of TILs or moderate-heavy.

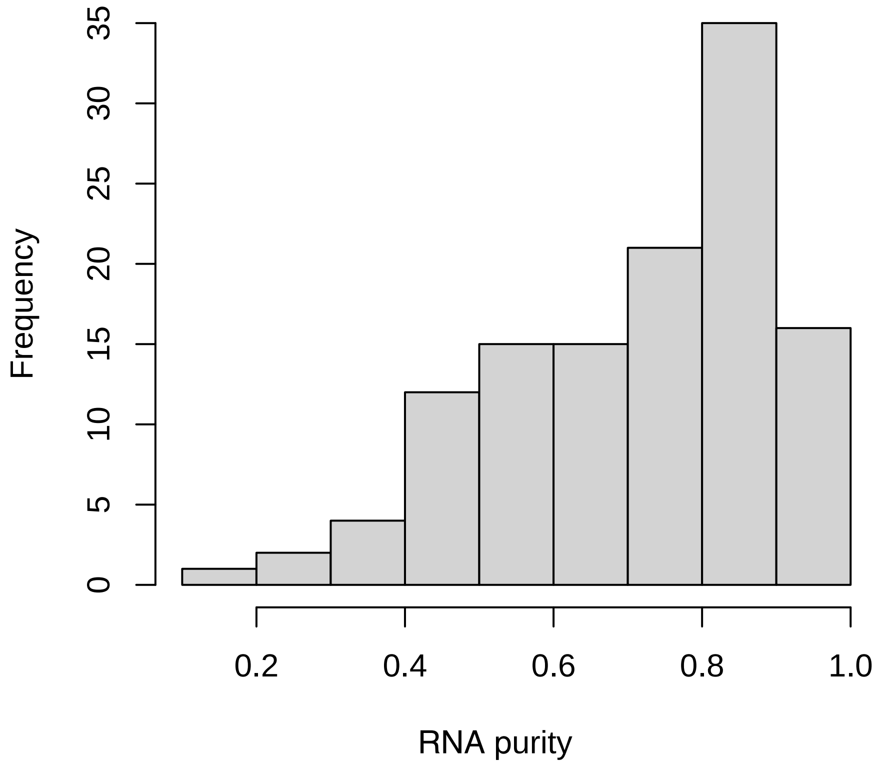

Supplementary figure 14: Histogram of purity for samples with RNA-seq data.

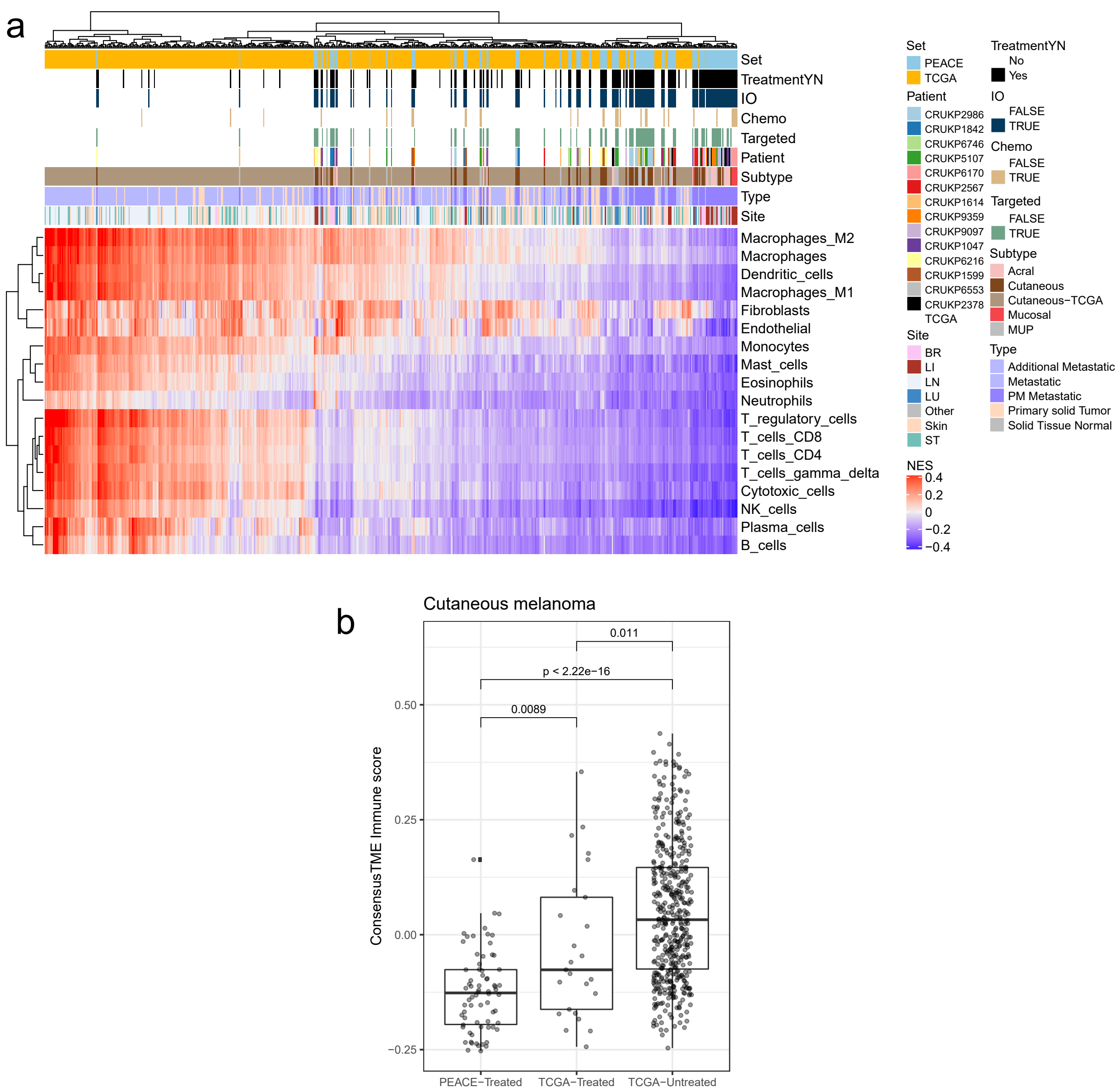

Supplementary figure 15: (a) TME deconvolution. Results from consensusTME analysis of transcriptome data. TCGA melanoma samples were run together with the melanoma PEACE samples. IO: Immunotherapy, BR: brain, LI: liver, LN: lymph node, LU: lung, ST: soft tissue. (b) The impact of treatment on immune infiltration. Immune scores from cutaneous melanoma samples across datasets and treatments. Wilcoxon rank-sum tests were used for the comparisons.

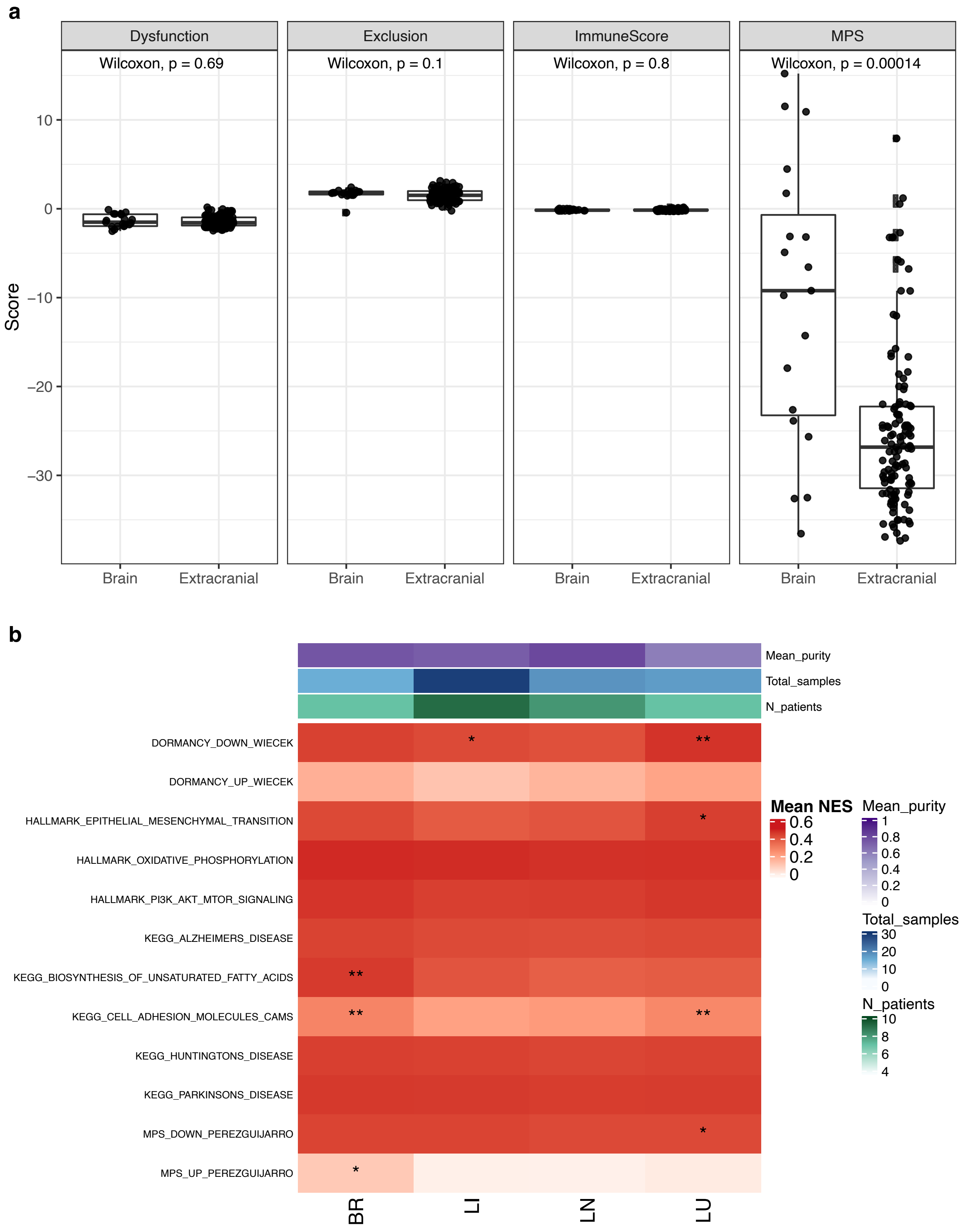

Supplementary figure 16: The effect of metastatic site on transcriptional profiles. (a) Brain vs extracranial metastatic samples. RNA signature scores: TIDE immune dysfunction and exclusion scores, consensusTME Immune Score and MPS plasticity score. (b) Pathways previously shown to be upregulated in brain metastases. Mean NES values per signature and site are shown, and the significance of site in a linear mixed effects model of NES by site and purity, with patient as a random effect is indicated by the asterisks (* p-value < 0.5, **, p-value < 0.01, *** p-value < 0.001).

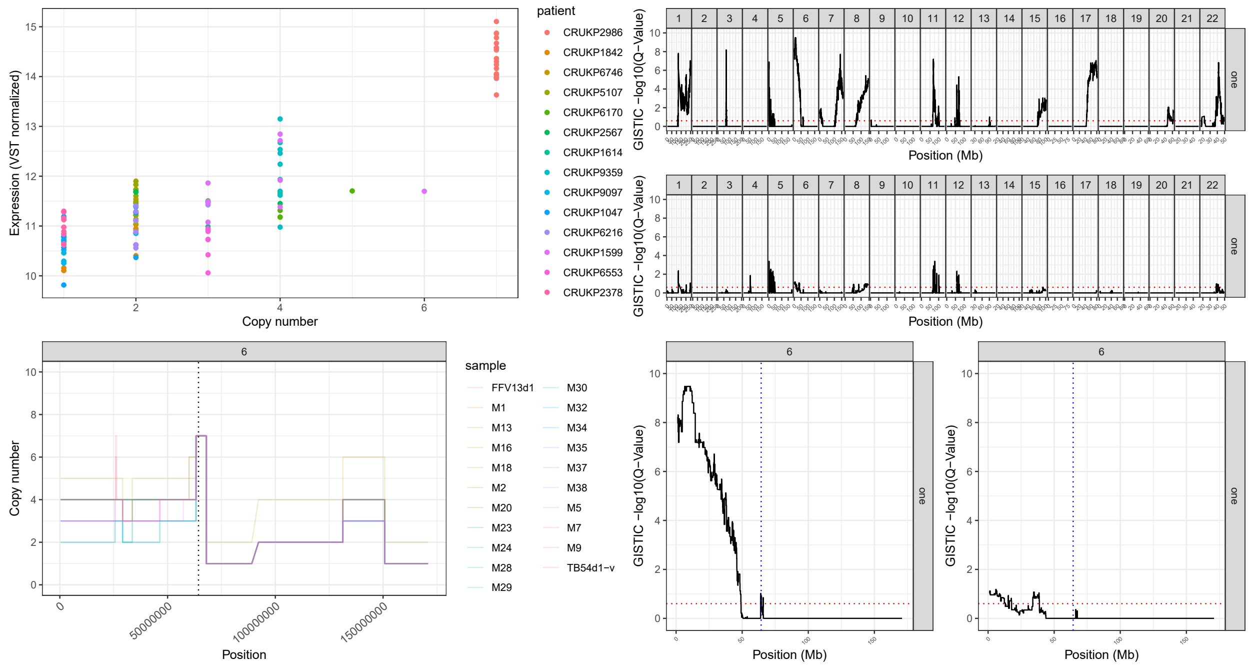

Supplementary figure 17: Association of PHF3 copy number with expression (left) as well as validation in TCGA data (right). PHF3 is significantly amplified in CSD melanomas but not non-CSD melanomas, although this might be explained by the smaller sample size of the latter.

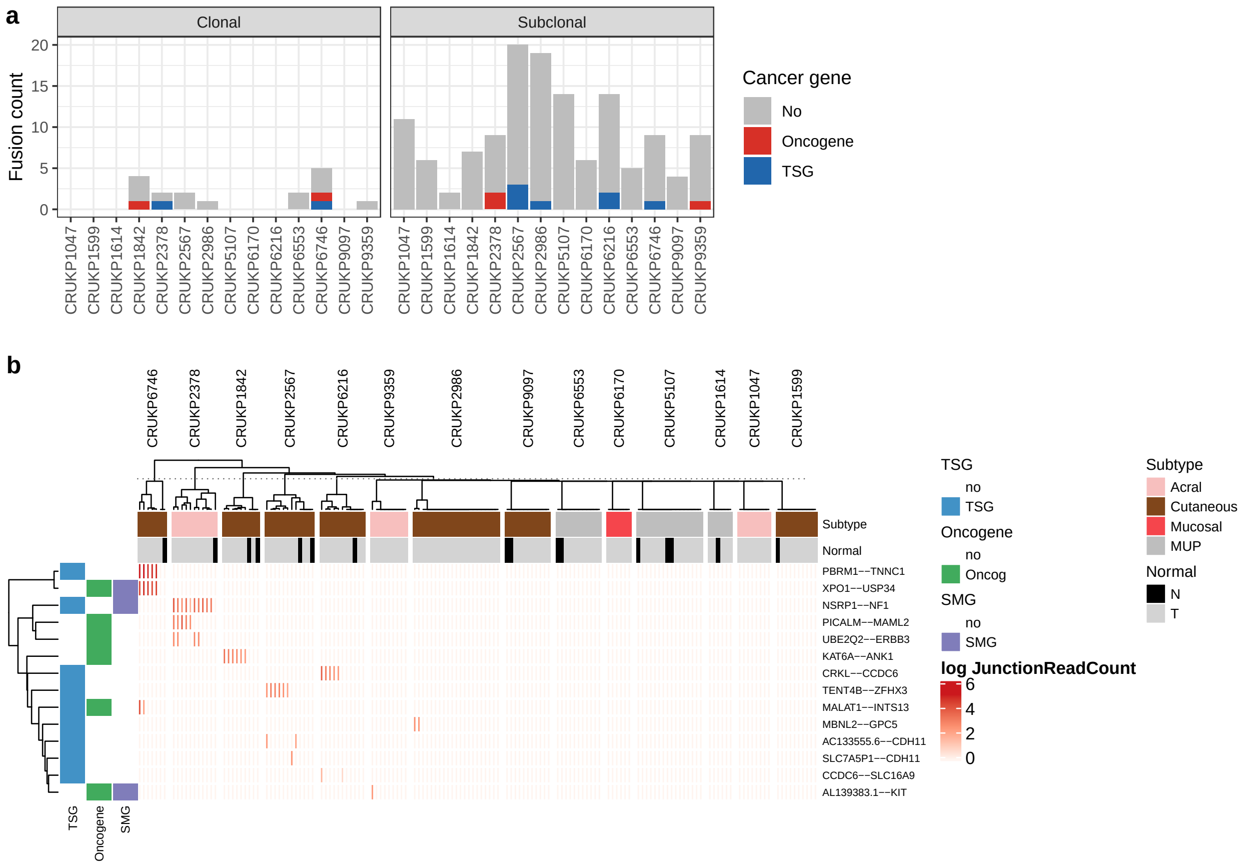

Supplementary figure 18: Overview of gene fusions identified in RNA-seq data. (a) Fusion counts by patient, stratified by clonality (clonal if in >80% of samples), and coloured by the type of gene affected. (b) Heatmap showing the presence of fusions of tumour suppressor genes, oncogenes or significantly mutated genes in melanoma (SMG) across samples (columns). Normal samples are annotated in black, while tumour samples are in grey.

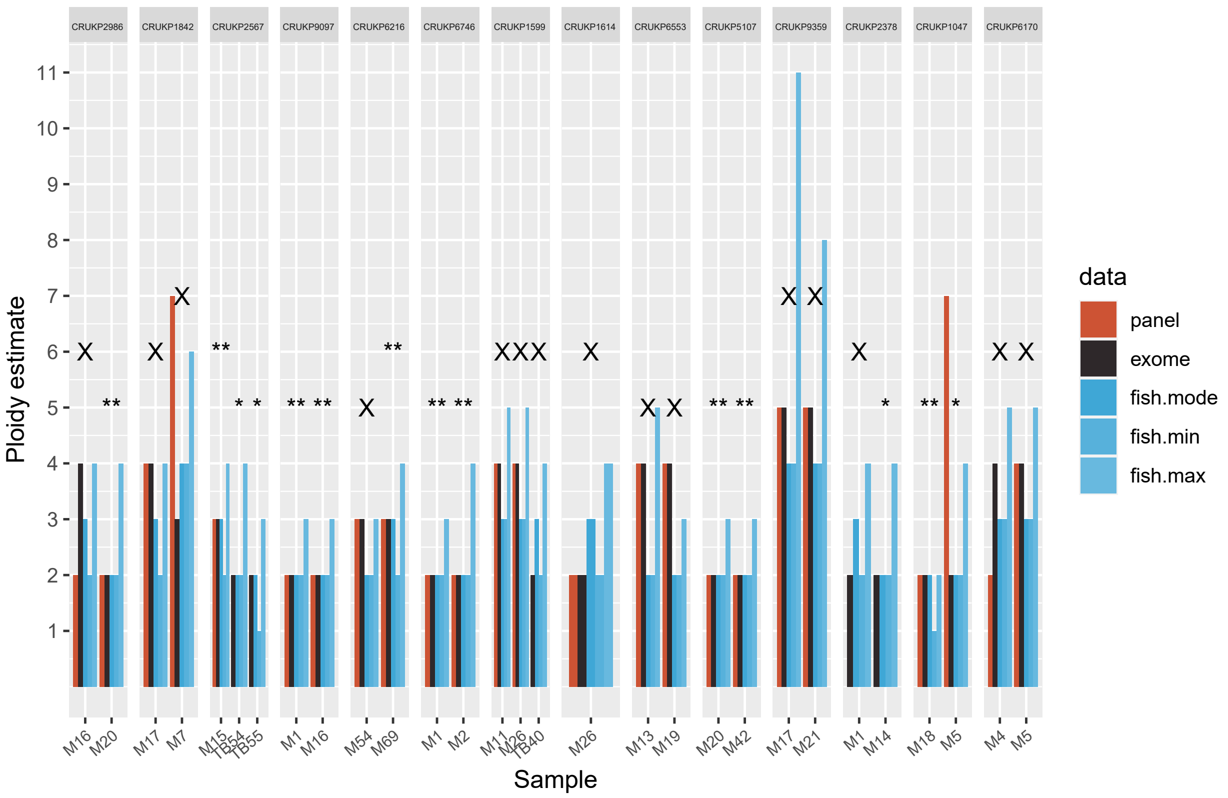

Supplementary figure 19: Comparison of ploidy estimates from panel sequencing data, exome data and FISH. ** indicates concordance between panel, exome and FISH modal ploidy. * indicates concordance between exome and FISH. X indicates lack of concordance between exome and FISH.

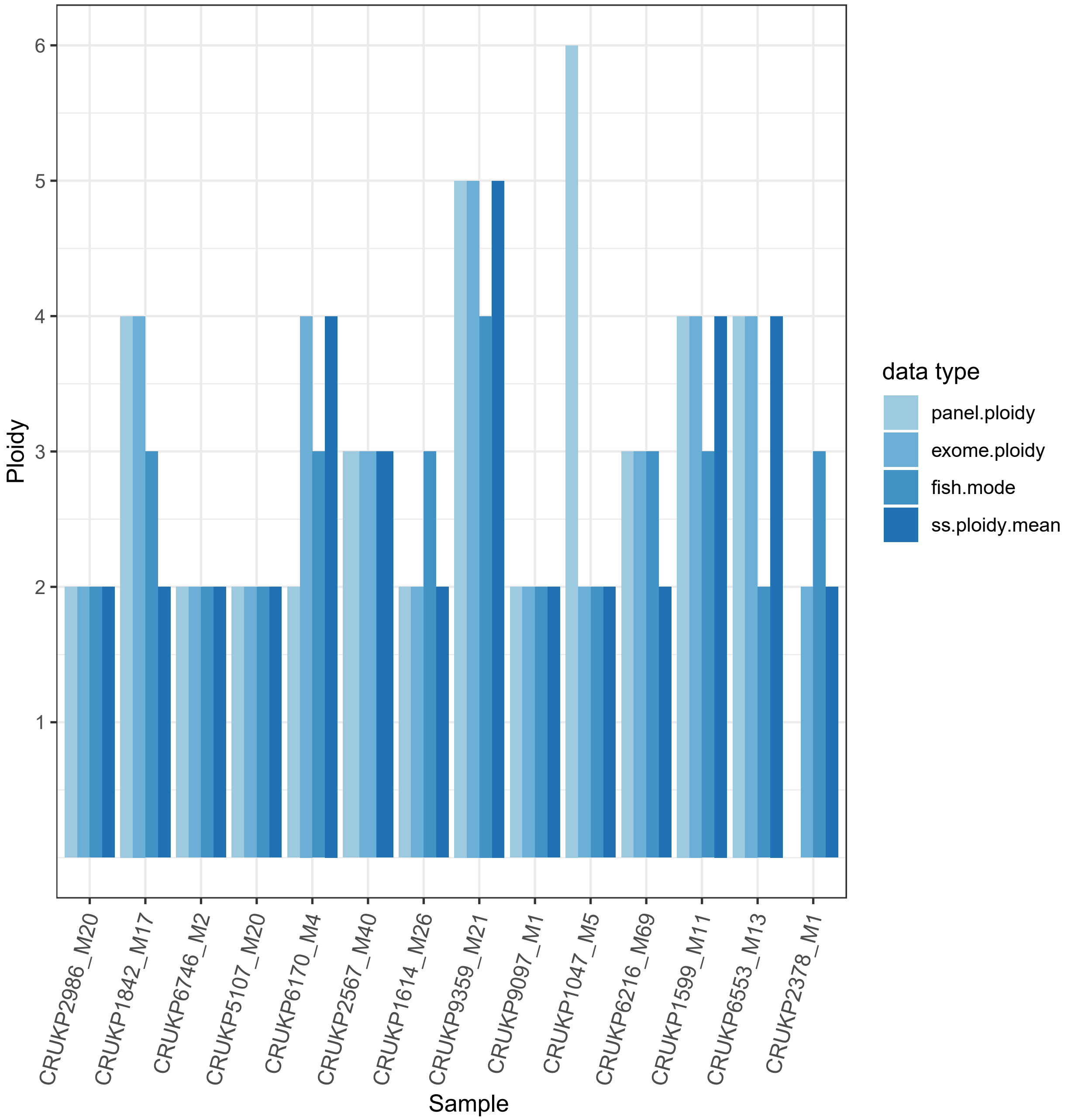

Supplementary figure 20: Comparison of ploidy estimates in panel, exome, FISH and single cell data.

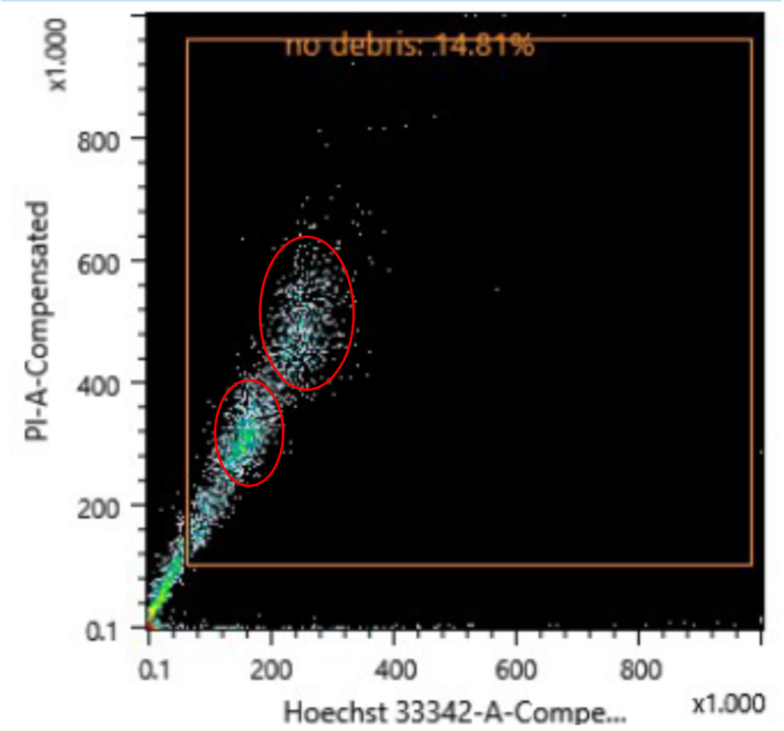

Supplementary figure 21: FACs sort plot for CRUKP2567 diaphragmatic metastasis.

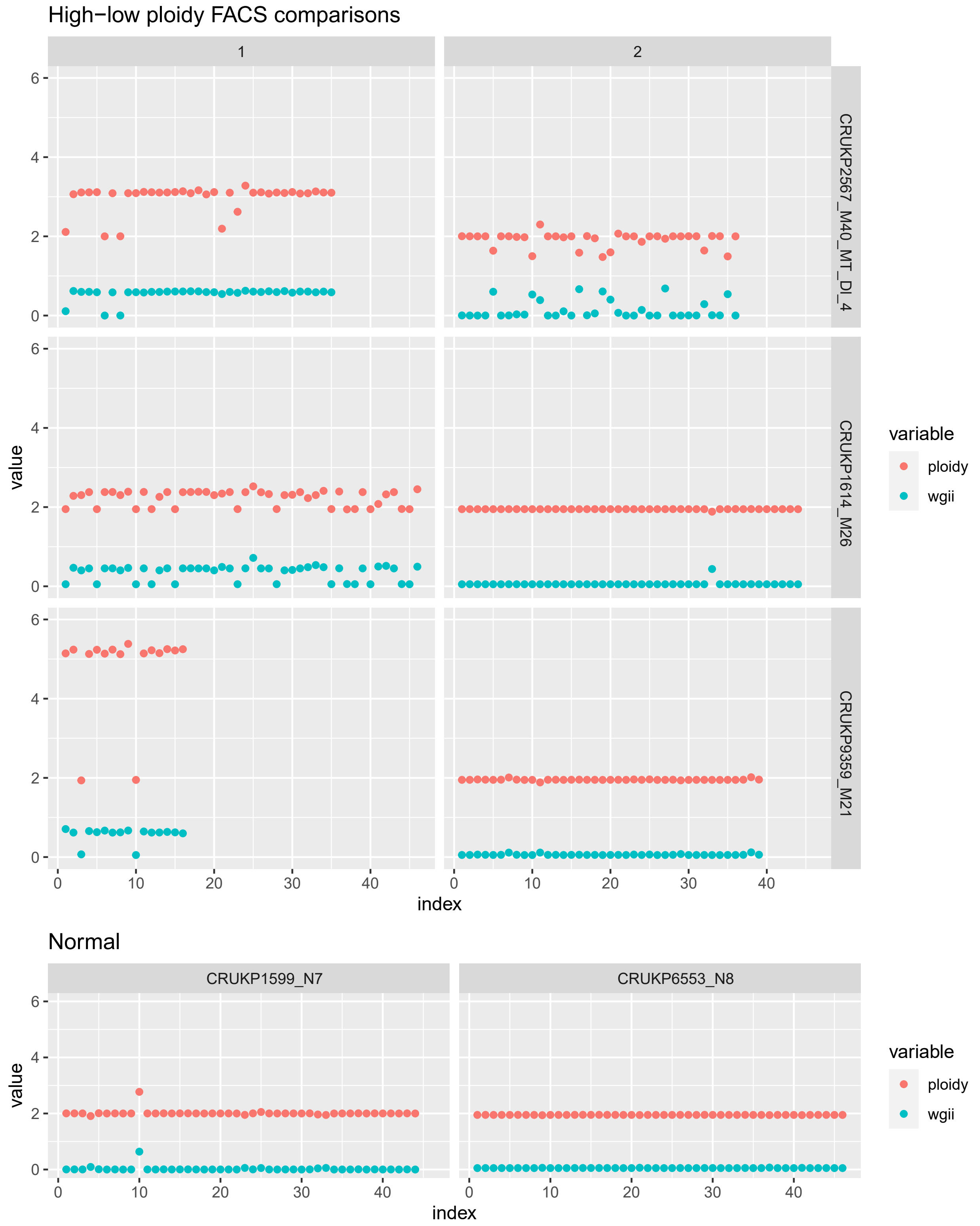

Supplementary figure 22: Ploidy and wGII values from single cell sequencing of FACS-sorted tumour cells. Left: high-ploidy cells from FACs; Right: low-ploidy cells from FACS. In samples CRUKP1614_M26 and CRUKP9359_M21, the low-ploidy FACs sorted cells have stable wGII, indicating that these are normal cells rather than tumour cells.

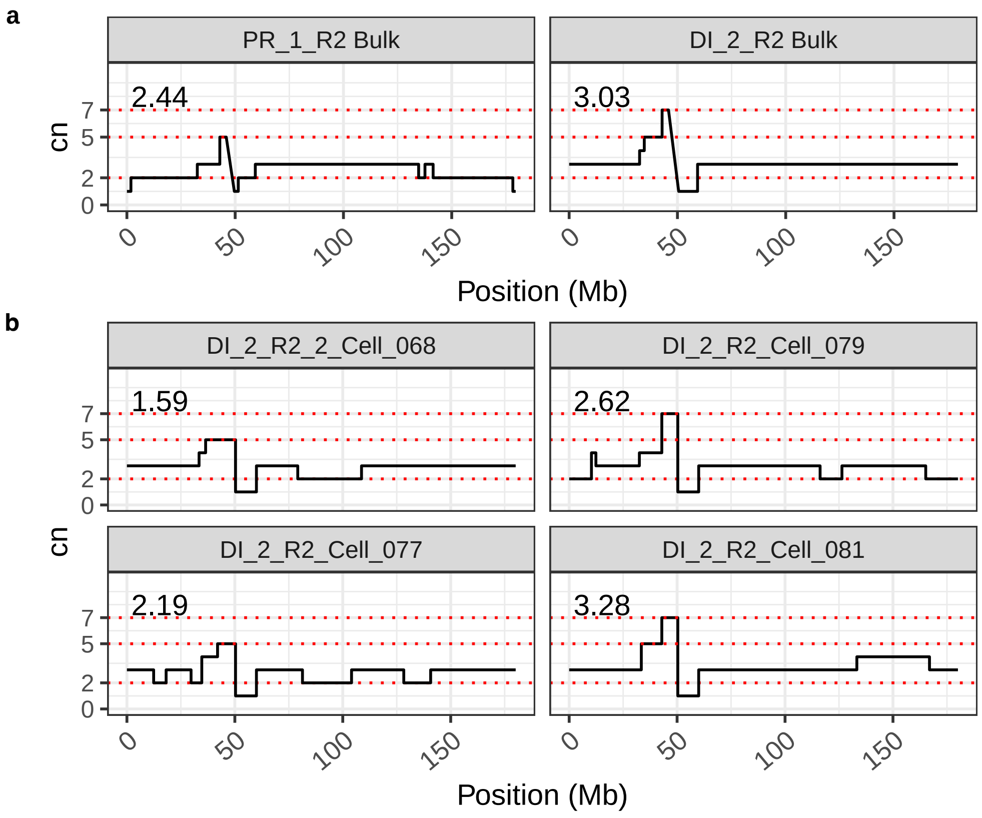

Supplementary figure 23: Copy number profiles on chromosome 5 for bulk samples from primary and DI_2_R2, a diaphragmatic metastasis. Ploidies for each sample are indicated in the top left. Dotted horizontal red lines indicate 2n, 5n and 7n respectively. (a) Bulk copy number profiles for chromosome 5 in a primary (PR) region and a diaphragm metastasis (DI). (b) Single-cell copy number profiles for chromosome 5 from DI_2_R2; the profiles on the left are more consistent with the primary, whilst the profiles on the right are more consistent with the bulk profile of the diaphragmatic metastasis DI_2_R2.

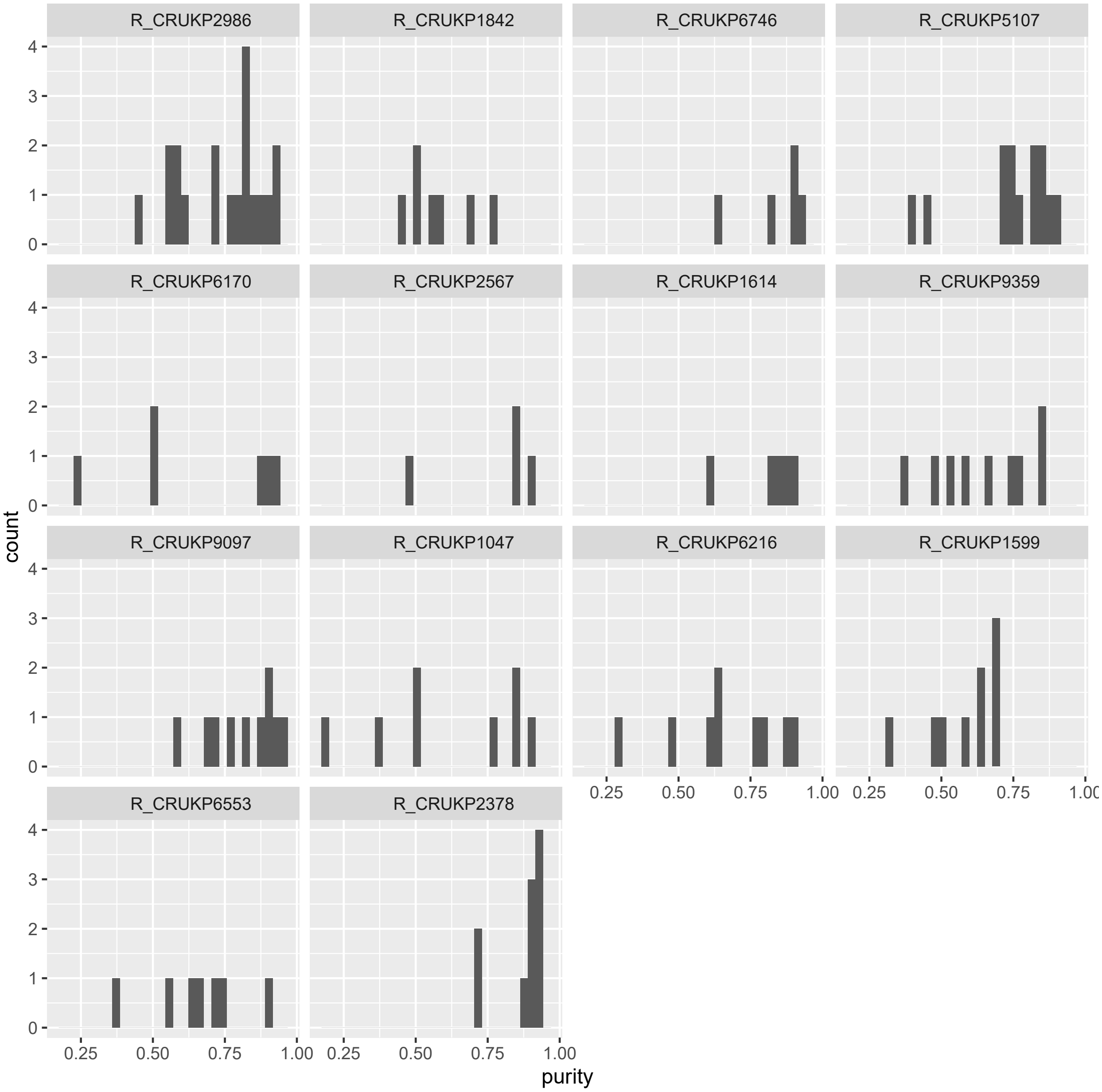

Supplementary figure 24: Histogram of purity of samples for which RNA-seq was performed faceted by patient

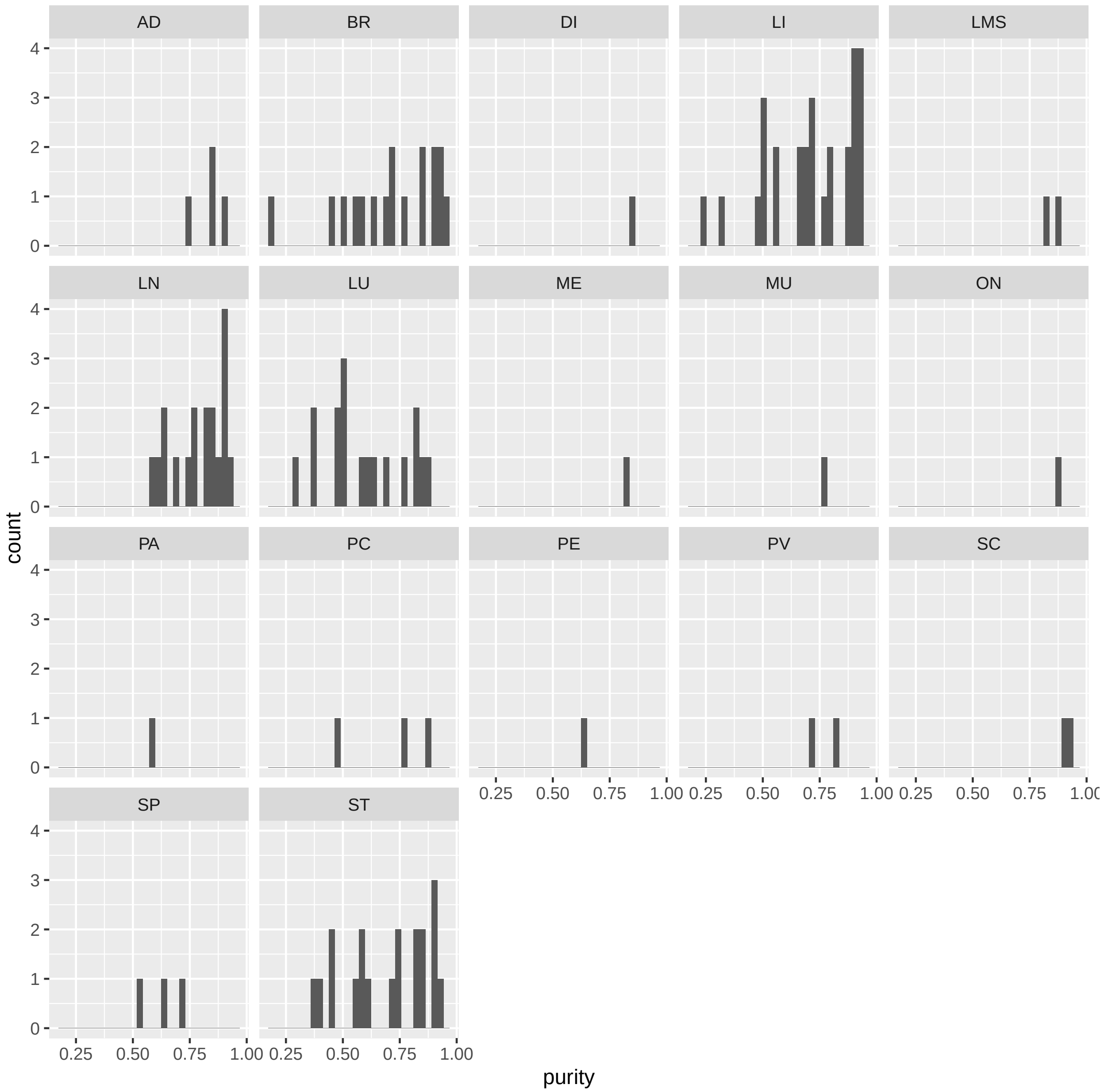

Supplementary figure 25: Histogram of purity of samples for which RNA-seq was performed faceted by tissue site
